# Binding mode analysis of ABCA7 for the prediction of novel Alzheimer's disease therapeutics

**DOI:** 10.1016/j.csbj.2021.11.035

**Published:** 2021-11-27

**Authors:** Vigneshwaran Namasivayam, Katja Stefan, Jens Pahnke, Sven Marcel Stefan

**Affiliations:** a Department of Pharmaceutical and Cellbiological Chemistry, Pharmaceutical Institute, University of Bonn, An der Immenburg 4, 53121 Bonn, Germany; b Department of Pathology, Section of Neuropathology, Translational Neurodegeneration Research and Neuropathology Lab (www.pahnkelab.eu), University of Oslo and Oslo University Hospital, Sognsvannsveien 20, 0372 Oslo, Norway; c LIED, University of Lübeck, Ratzeburger Allee 160, 23538 Lübeck, Germany; d Department of Pharmacology, Faculty of Medicine, University of Latvia, Jelgavas iela 1, 1004 Rīga, Latvia

**Keywords:** ABC, ATP-binding cassette, AD, Alzheimer’s disease, APP, amyloid precursor protein, ATP, Adenosine-triphosphate, BBB, blood-brain barrier, BODIPY-cholesterol, 4,4-difluoro-4-bora-3a,4a-diaza-s-indacene-cholesterol, cryo-EM, cryogenic-electron microscopy, EH, extracellular helix, ECD, extracellular domain, GSH, reduced glutathione, HTS, high-throughput screening, IC, intracellular helix, MOE, Molecular Operating Environment, MSD, membrane spanning domain, NBD, nucleotide binding domain, NBD-cholesterol, 7-nitro-2-1,3-benzoxadiazol-4-yl-cholesterol, PDB, protein data bank, PET, positron emission tomography, PLIF, protein ligand interaction, PSO, particle swarm optimization, R-domain/region, regulatory domain/region, RMSD, root mean square distance, SNP, single-nucleotide polymorphism, TM, transmembrane helix, ABC transporter (ABCA1, ABCA4, ABCA7), Alzheimer’s disease (AD), Multitarget modulation (PANABC), PET tracer (PETABC), Polypharmacology, Rational drug design and development

## Abstract

The adenosine-triphosphate-(ATP)-binding cassette (ABC) transporter ABCA7 is a genetic risk factor for Alzheimer’s disease (AD). Defective ABCA7 promotes AD development and/or progression. Unfortunately, ABCA7 belongs to the group of ‘under-studied’ ABC transporters that cannot be addressed by small-molecules. However, such small-molecules would allow for the exploration of ABCA7 as pharmacological target for the development of new AD diagnostics and therapeutics. Pan-ABC transporter modulators inherit the potential to explore under-studied ABC transporters as novel pharmacological targets by potentially binding to the proposed ‘multitarget binding site’. Using the recently reported cryogenic-electron microscopy (cryo-EM) structures of ABCA1 and ABCA4, a homology model of ABCA7 has been generated. A set of novel, diverse, and potent pan-ABC transporter inhibitors has been docked to this ABCA7 homology model for the discovery of the multitarget binding site. Subsequently, application of pharmacophore modelling identified the essential pharmacophore features of these compounds that may support the rational drug design of innovative diagnostics and therapeutics against AD.

## Graphical abstract

**Figure:**
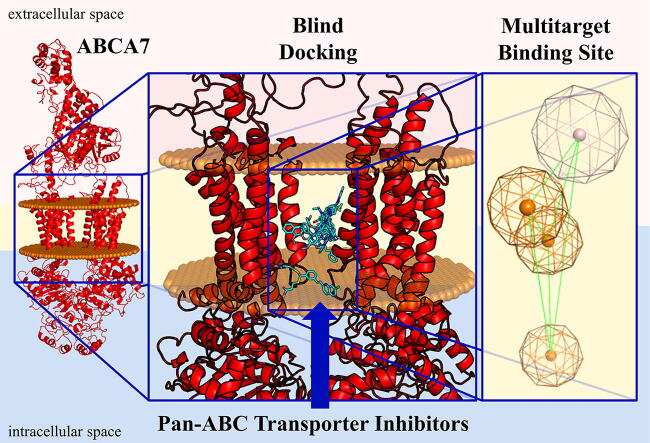

## 1 Introduction

ABC transporters form the backbone of systemic barrier formation [1], [2] and are key factors in inter- and intracellular compartmentalization, allowing for the existence of opposing cellular processes. ABC transporter function has not only been demonstrated in the cellular membrane but also intracellularly in vesicular bodies [3], [4], [5]. Several representatives of the ABC transporter superfamily stand in association with the constitution and composition of cellular as well as vesicular membranes and microparticles [4], [6], such as certain A and G subfamily members. Furthermore, a contribution of these transporters to the formation and organization of lipid rafts has been discussed [4], [6], [7]. These lipid rafts are the venue of very important membrane-associated cellular processes, for example, secretase-mediated amyloid precursor protein (APP) processing, a process critical for the initiation and progress of AD [8], [9], [10].

Most ABCA and ABCG transporters are cholesterol and/or phospholipid transporters that regulate cellular lipid homeostasis [11], [12], which is not only linked to membrane permeability and barrier function but also to human diseases, such as AD [6], [13], [14], [15], [16], [17], [18], [19], [20], [21]. The lipid transporters ABCA1–2, ABCA5, ABCA7, ABCG1, and ABCG4 have particularly been associated with AD development [6], [13], [19], [22], [23], [24], [25], [26], [27], [28]. Genetic variant association studies regarding ABCA1 have revealed controversial information related to certain single-nucleotide polymorphisms (SNPs) and AD [6], [25], [29], [30], [31], [32], [33]. However, genome-wide association studies provided statistical proof that ABCA7 is strongly associated with AD development and/or progression [13], [24], [25], [34], [35], [36], [37]. Certain polymorphisms were associated with plaque formation in patients, which was correlated to an increased expression of ABCA7, supposedly as compensation for the increased Aβ load, suggesting an inhibition of Aβ deposition [22]. In addition, *Abca7* knock-out led to Aβ load increase, while the overexpression of ABCA7 led to reduction of Aβ in mice [22]. ABCA7 has functionally be linked to cholesterol metabolism and phagocytosis, which may influence Aβ distribution and degradation [22].

**Fig. 1:**
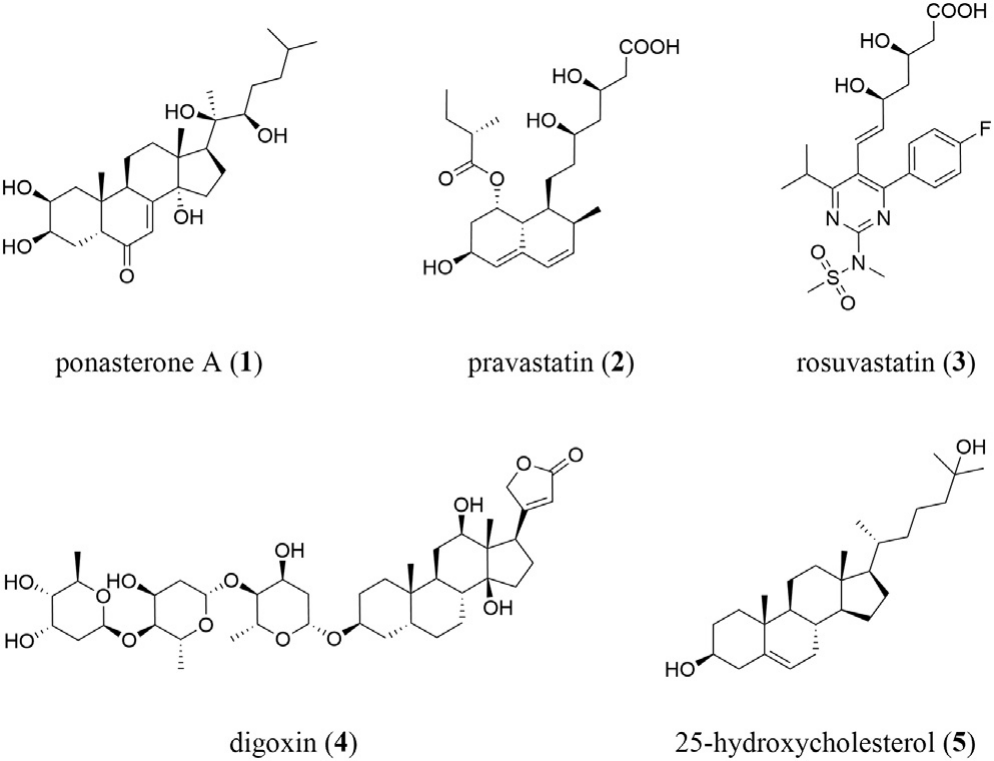
The only known small-molecule modulators of ABCA7.

Unfortunately, ABCA7 belongs to the so-called ‘under-studied’ ABC transporters that cannot be addressed by small-molecules [38]. Besides the substrates cholesterol and phospholipids [12], [13], [24], [34], the only small-molecules associated with ABCA7 are regulators of ABCA7 expression. These regulators include the inducers ponasterone A [39], pravastatin [40], [41], and rosuvastatin [40], as well as the downregulators digoxin [42] and 25-hydroxycholesterol [43] (all Fig. 1). These regulators are only of very limited use to monitor and/or influence the transport process, as they do not directly interact with ABCA7. However, deciphering this particular functional background would allow for the targeted development of novel AD diagnostics and therapeutics.

A recently proposed approach to promote the development of directly-interacting under-studied ABC transporter-targeting agents is the generation and application of multitarget ABC transporter (‘pan-ABC transporter’) inhibitors to obtain novel lead structures for future diagnostic and therapeutic development [4], [38], [44], [45], [46]. A common ‘multitarget binding site’ of these modulators amongst the different ABC transporter subfamilies has been proposed [4], [38], [44], [45], which may serve as a starting point for rational drug design, exploration, and exploitation of under-studied ABC transporters as (polypharmacological) drug targets.

Since ABCA7 activity is believed to be reduced in AD [13], [25], activators (increase transport function) or correctors (restore functional conformation) are needed, and the use of ABC transporter inhibitors seems counterintuitive. However, although activators (no separation of, for example, ABCB1 and [47], [48], ABCC1 [47], [49], [50], ABCC2 [51], ABCC3 [51], ABCC8 [52], [53], [54], [55], or ABCC9 [52], [53], [55]) and correctors (*e.g.*, for ABCA3 [56], ABCA4 [57], [58], ABCB4 [59], ABCB11 [60], or ABCC7 [61]) have been described in the literature, the knowledge of such agents is very scarce, undermining the concept of exploration of under-studied ABC transporters by a multitargeting approach. Luckily, the knowledge of pan-ABC transporter inhibitors is broader [45].

Additionally, it must be noted that several activators of ABC transporters share molecular-structural features of ABC transporter inhibitors. For example, tetrahydroisoquinolines (ABCB1) [48], [62], [63] and pyrrolopyrimidines (ABCC1) [49], [64] have been described for both activators [48], [49] and inhibitors [62], [63], [64] of ABCB1 and ABCC1, respectively. Other examples are the compound classes of phenothiazines [65], [66] and purines [49], [67], [68], which have been described to comprise of ABCC1 activators [49], [65], [66] and ABCB1 inhibitors [65], [66], [67], [68]. More examples can be found in the literature [47], [69]. Considering these findings, gained knowledge about the multitarget binding site by the use of multitargeting inhibitors may indeed provide necessary molecular-structural information of active scaffolds/substructures for the targeted design of ABCA7 activators (and/or correctors). In addition, imaging techniques using ABC transporter inhibitors have already been demonstrated to trace the expression of the respective ABC transporter *in vivo* [63], making ABC transporter inhibitors that are not substrates of the respective ABC transporter (*e.g.*, ABCA7) eligible for, *e.g.*, positron emission tomography (PET) as diagnostics in patients.

**Fig. 2:**
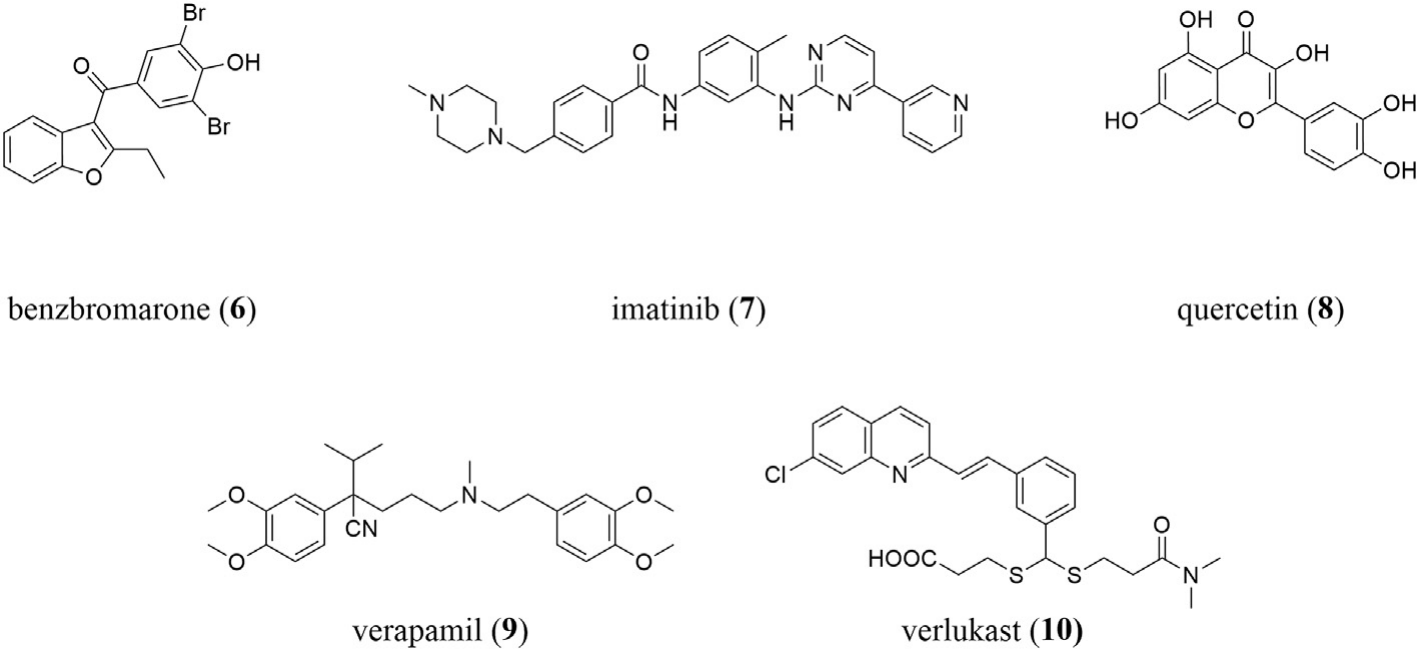
Drugs and drug-like compounds that were discovered as truly multitarget pan-ABC transporter inhibitors.

Several moderately potent pan-ABC transporter inhibitors have been discovered in recent years. Table 1 summarizes selected candidates (**6**–**28**) and their associated physicochemical properties. These pan-ABC transporter inhibitors can principally be divided into two groups: (i) truly multitarget pan-ABC transporter inhibitors and (ii) focused pan-ABC transporter inhibitors. Truly multitarget pan-ABC transporter inhibitors are mostly drugs and drug-like candidates that have been known for a long period of time to interfere with specific ABC transporters [45], [69], [70], [71], [72], [73] and were broadly evaluated toward several members. These compounds include benzbromarone (**6**; ABCB1 [74], ABCB11 [75], ABCC1–6 [69], [73], [76], [77], [78], ABCG2 [74]), imatinib (**7**; ABCA3 [79], ABCB1 [80], ABCB11 [75], ABCC1 [80], ABCC10 [80], ABCG2 [80]), quercetin (**8**; ABCB1 [76], ABCC1–2 [69], [76], ABCC4–5 [81], [82], ABCC11 [83], ABCG2 [69], ABCG6 [84]), verapamil (**9**; ABCA8 [85], ABCB1 [70], ABCB4–5 [86], [87], ABCB11 [88], ABCC1 [69], ABCC4 [89], ABCC10 [90], ABCG2 [76]), and verlukast (MK571, **10**; ABCA8 [85], ABCB4 [86], ABCB11 [75], ABCC1–5 [69], [76], [91], [92], [93], ABCC10–11 [73], [94], ABCG2 [76]), all Fig. 2.

**Table 1:** Selected pan-ABC transporter inhibitors that have been reported in the literature as well as associated physicochemical and molecular-structural properties determined by using InstandJChem version 20.15.0.

**Compd.** **No.**	**Original** **Name**	**Molecular** **Weight**	**Calc** **Log P**	**Rotatable** **Bonds**	**H-bond** **Acceptors**	**H-bond** **Donors**	**Targeted** **ABC Transporters**
*Truly Pan-ABC Transporter Inhibitors*							
**6**	benzbromarone	424.09	5.55	3	2	1	B1, B11, C1–6, G2
**7**	imatinib	493.62	4.38	7	7	2	A3, B1, B11, C1, C10, G2
**8**	quercetin	302.24	2.16	1	7	5	B1, C1–2, C4–5, C11, G2, G6
**9**	verapamil	454.61	5.04	13	6	0	A8, B1, B4–5, B11, C1, C4, C11, G2
**10**	verlukast	515.08	5.67	11	4	1	A8, B4, B11, C1–C5, C10–C11, G2
*Focused Pan-ABC Transporter Inhibitors*							
**11**	imidazole/pyrimidine 23	438.54	4.35	5	5	0	B1, C1, G2
**12**	quinoline/1,2,4-oxadiazole 15	421.45	4.11	7	7	0	B1, C1, G2
**13**	quinoline/1,3,4-thiadiazole 18	446.50	4.85	4	6	0	B1, C1, G2
**14**	quinazoline/1,2,4-oxadiazole 21	444.42	5.24	7	7	0	B1, C1, G2
**15**	quinoline/thieno[3,2-*c*]pyridine 22	424.54	3.31	2	5	0	B1, C1, G2
**16**	quinoline/1,2,4-oxadiazole 26	391.48	4.12	5	6	0	B1, C1, G2
**17**	pyrimidine 26	379.42	5.82	5	6	3	B1, C1, G2
**18**	tariquidar-related derivative 40	501.54	3.52	8	8	1	B1, C1, G2
**19**	pyrimdine 37	312.38	4.68	3	4	1	B1, C1, G2
**20**	amino aryl ester (*S*)-9	631.76	5.84	23	9	0	B1, C1, G2
**21**	thienopyrimidine 14	421.56	4.32	4	5	1	B1, C1, G2
**22**	pyrrolopyrimidine 55	489.63	5.73	8	5	1	B1, C1, G2
**23**	indolopyrimidine 69	357.46	4.12	4	4	1	B1, C1, G2
**24**	quinazoline 52	387.44	5.12	6	6	1	B1, C1, G2
**25**	quinoline 29	342.46	6.23	4	2	1	B1, C1, G2
**26**	thienopyridine 6r	566.63	4.52	12	9	1	B1, C1, G2
**27**	benzoflavone 16	332.36	3.64	3	4	0	B1, C1, G2
**28**	MC18	393.53	4.90	6	4	0	B1, C1, G2

**Fig. 3:**
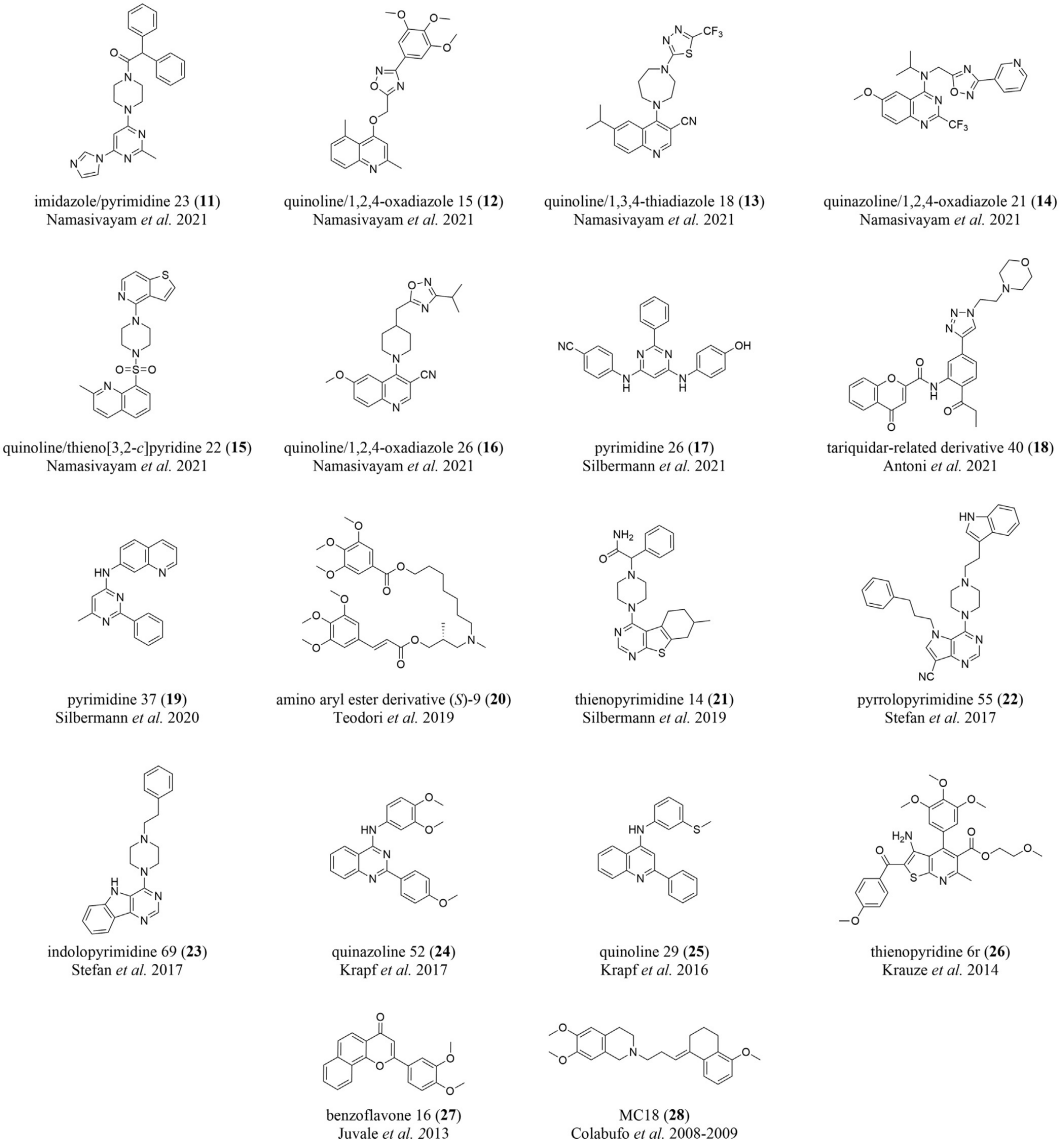
Selection of focused pan-ABC transporter inhibitors used in the present study.

Focused pan-ABC transporter inhibitors are high-throughput screening-(HTS)- and/or organic synthesis-derived small-molecules specifically designed to target the well-studied ABC transporters ABCB1, ABCC1, and ABCG2 [38], [45]. These are very modern molecules that were not evaluated at other transporters yet. The most potent representatives were designated as so-called ‘class 7′ molecules [38], [44], [45] – which exert their inhibitory power against ABCB1, ABCC1, and ABCG2 below 10 µM. A selection of candidates (**11**–**28)**, which were also used in the present study, are shown in Fig. 3.

Prospective computational approaches, such as molecular docking, support the search for potential binding sites of ligands, and thus, rational drug design in general [38]. However, molecular docking and binding pose analyses require structural information of the respective transporters or closely related siblings. Unfortunately, no X-ray structure of ABCA7 is available. Indeed, a cryo-EM structure of ABCA7 has already in 2020 been announced [95] on the protein data bank (https://www.rcsb.org; PDB ID: 7KQC; PDB status: unreleased deposition withdrawn) and bioRxiv (https://www.biorxiv.org), however, the respective structural information is at the time of writing of this manuscript not publicly available and cannot be used for subsequent computational operations. Luckily, cryo-EM structures of ABCA1 (4.1 Å) [96] and ABCA4 (3.3–3.6 Å) [97], [98], [99] have recently been reported, allowing for homology modelling and generation of new structural information. Homology models of other less- and under-studied ABC transporters in combination with molecular docking experiments led already to the discovery of novel bioactive molecules, as for example, for ABCC5 [100] or ABCG2 [101]. This poses a good perspective for the design and discovery of new interactors and putative binding sites of ABCA7.

The present study aimed for the development of an ABCA7 homology model and subsequent blind docking analysis of the membrane-spanning domains (MDDs) with the pan-ABC transporter inhibitors **6**–**28**, as the multitarget biding site that is supposed to be shared amongst several subfamily members of ABC transporters was postulated to be located in the proximity of the MSDs [4]. This postulation is supported by cryo-EM (ABCB1 [102], ABCB2 [103], ABCB4 [104], ABCC8 [105], and ABCG2 [106]) and homology model data (ABCB1 [107], ABCB2 [108], ABCB5 [109], ABCC1 [110], ABCC4 [111], ABCC5 [100], ABCC6 [112], ABCC7 [113], ABCC11 [114], and ABCG2 [101]) of other ABC transporters which had their primary substrate/inhibitor binding sites within the transmembrane regions.

The herein presented blind docking experiments identified a putative binding pocket of a majority of the 23 selected molecules that reflected a common pharmacophore. These discoveries have broad implications regarding the development of novel AD diagnostics and therapeutics.

## 2 Results and discussion

### 2.1 Sequence alignment

Supplementary Fig. S1 compares the canonical amino acid sequences of ABCA1 (UniProt ID: O95477), ABCA4 (UniProt ID: P78363), as well as ABCA7 (UniProt ID: Q8IZY2), and important structural elements have been marked. ABCA7 is from the structural perspective a ‘common’ ABCA transporter. It is large in size, consisting of 2,146 amino acids, comparable to the 2,261 and 2,273 amino acids of ABCA1 [96] and ABCA4 [97], [98], [99], respectively. ABCA7 shares 52.8% and 48.2% sequence homology with ABCA1 and ABCA4, respectively, which is in agreement with the literature data (54% and 49%, respectively) [24], [34], [115]. Functionally, ABCA1 and ABCA7 are closely related, recognizing and translocating cholesterol and phospholipids [12], [13], [24], [34], while ABCA4 is mainly a retinoid transporter [12]. Considering its greater similarity in terms of the amino acid sequence and its closer functional similarity, the generation of the ABCA7 homology model has been accomplished taking only the cryo-EM structure of ABCA1 into account [96]. However, for the sequential and structural interpretation, in particular in terms of phospholipid binding, specific structural aspects of the ABCA4 cryo-EM structures have been compared to the obtained homology model as model validation [97], [98], [99].

### 2.2 Homology model

**Fig. 4:**
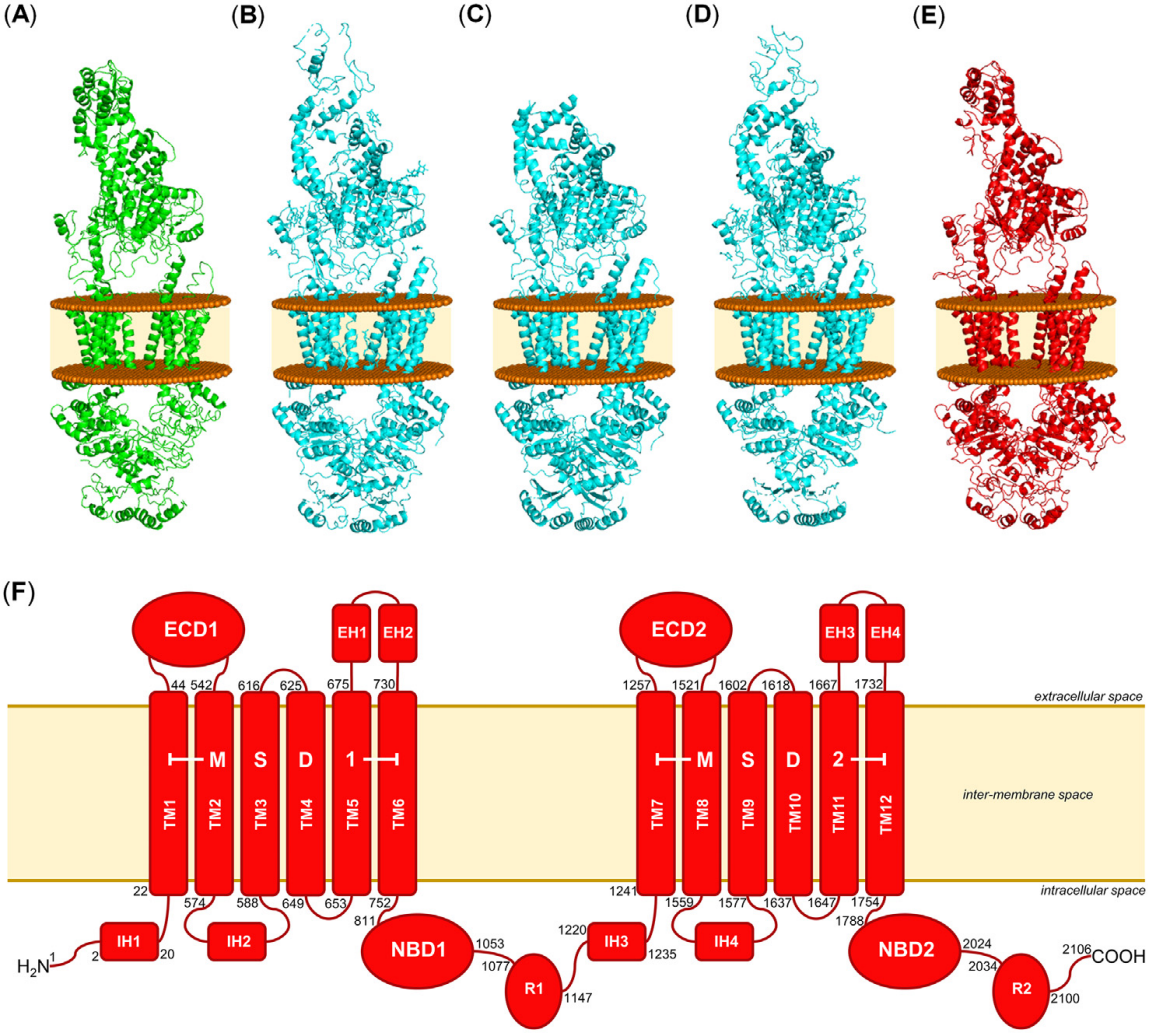
Structural information and conformational representation of ABCA transporters that can be found in the literature [96], [98], [99] using Molecular Operating Environment (MOE) version 2019.01 [118]. (A) Cryo-EM structure of human ABCA1 as reported by Qian *et al.* in 2017 [96]. (B) Cryo-EM structure of human ABCA4 as reported by Xie *et al.* in 2021 [98]. (C) Cryo-EM structure of human ABCA4 as reported by Liu *et al.* in 2021 [99]. (D) Cryo-EM structure of human ABCA4 as reported by Scortecci *et al.* in 2021 [97] (E) Homology model of human ABCA7 generated from the cryo-EM structure of human ABCA1 [96]. The membrane bilayers in (A)–(E) are indicated as brown balls and light brown areas. (F) simplified scheme of the organization of ABCA7 and its structural components. The inter-membrane space is indicated as light brown area, and the border to the cytosol and lumen is indicated by brown lines.

Fig. 4 (A)–(D) provides all structural information available on ABCA transporters, namely ABCA1 [(A), PDB ID: 5XJY [96]] and ABCA4 [(B), PDB ID: 7E7I [98]; (C), PDB ID: 7LKP [99]; (D), PDB ID: 7E7O [97]]. The underlying cryo-EM structures of ABCA1 and ABCA4 had resolutions of 4.1 Å [96] and 3.3–3.6 Å [97], [98], [99], respectively. Furthermore, Fig. 4 (E) shows the modelled ABCA7 structure according to the structural composition and orientation of ABCA1 [96], and Supplementary Fig. S2 provides the Ramachandran plot of the modelled ABCA7. The overall quality factor [116] of the model is 87%. This is a rather low value that stems from the fact that ABCA7 is a very large protein of 2146 amino acids. Structural templates with high resolutions of large proteins like ABCA7 have rarely been assessed in terms of model quality, and hence, these results cannot accurately be put into perspective. Nevertheless, the low value is exclusively based on the highly-flexible loop and ECD regions, which were particularly not focused in the present study.

In terms of its structural organization, ABCA7 is – like ABCA1 [96] and ABCA4 [97], [98], [99] as well – a so-called ‘full transporter’, consisting of two membrane spanning domains (MSD1 and MSD2), each composed of six transmembrane helices (TM1–6 and TM7–12). As already described for ABCA1 [96] and ABCA4 before [98], TM1–6 (MSD1) as well as TM7–12 (MSD2) represent separate entities without large intermolecular interactions between the two MSDs, which is in contrast to a swapped/twisted transmembrane structure, as this is the case, for example, for ABCB1 [102], [106]. This separation of the MSDs is a typical construct of so-called ‘type II’ transporters and has first been described for the model type II transporter, ABCG5/ABCG8 (PDB ID: 5DO7 [117]), which is also a lipid transporter [11]. Only TM5 and TM11 form a contact zone, as demonstrated for ABCA1 [96] and ABCA4 [97], [98], [99]. The other helices of ABCA7 are completely exposed to the hydrophobic inter-membrane space, potentially for attraction of and interaction with membrane-bound/solved cholesterol and phospholipids before these molecules are guided into the substrate/drug translocation cavity. This conformational orientation is called the ‘lateral-opening’ conformation [98]. The substrate/drug translocation cavity is formed by the two MSDs and stretches from the cytoplasmic to the luminal side through the whole membrane bilayer. Indeed, residues of TM1–2 and TM6 of ABCA1 have been proposed to form a ‘phospholipid binding pocket’ [96], and the structural data of ABCA4 revealed several binding sites for phospholipids and N-retinylidene-phosphatidylethanolamine [97], [98] within the cavity built by the two MSDs.

Structural similarity between ABCA1 [96], ABCA4 [97], [98], [99], ABCG5/ABCG8 [117], and ABCA7 can also be seen in terms of 4 intracellular helices (IHs) between TM1–2, TM3–4, TM7–8, and TM9–10. These IHs are typical in type II transporters and are believed to provide the necessary flexibility for the allosteric communication between the MSDs and NBDs in the substrate/drug translocation process [117]. The same accounts for 4 extracellular helices (EHs) between both TM5–6 and TM11–12.

MSD1 and MSD2 of ABCA7 are flanked by one nucleotide-binding domain (NBD1 and NBD2) each at the carboxy terminus of the respective MSD within the cytoplasm. These NBDs bind and cleave ATP to ADP and P_i_ and generate the necessary energy for active transport. Both NBDs of ABCA7 bear the Walker A (GXXGXGKS/T; X  = any amino acid) [119], [120] and Walker B (XXXXD; X  = hydrophobic amino acid) [119], [120] motifs as well as the ABC signature motif (NBD1: LSGGM; NBD2: YSGGN) [99], [119], which together form the most highly conserved features amongst all ABC transporters [119], [120]. Interestingly, the carboxy termini of both NBDs are occupied by one regulatory domain each (‘R-domain’/‘R-region’; R1 and R2), which are suggested to stabilize NBD1/NBD2 interaction [96], [98] and strongly interact with one another in the absence of ATP [99].

ABCA7 bears also two extracellular domains (ECD1 and ECD2), which represent special structural features of ABCA transporters [96], [97], [98], [99]. These domains are not present in other ABC transporter subfamilies. ECD1 and ECD2 elongate ABCA7 to a total height of ∼216 Å, comparable to ∼200 Å of ABCA1 [96] and ∼230–240 Å of ABCA4 [97], [98], [99]. ECD1 is located between TM1–2, while ECD2 is located between TM7–8. Both ECDs form a hydrophobic channel embedded in the intraluminal body of ∼105 Å height as already found particularly for ABCA1 (∼100 Å)[96] and ABCA4 (∼130 Å) [99] before. The ECDs are composed of 506 and 273 amino acids, respectively, compared to 583 and 270 amino acids of ABCA1 [96] as well as 600 and 290 amino acids of ABCA4 [99]. This channel is similar to the discovered ‘base-tunnel-lid’ flame-shaped body as described for ABCA1 [96] and ABCA4 [98] before, with strong interactions of the ‘base’ and the formed ‘tunnel’. The tunnel of ABCA7 is composed of – similar to ABCA1 [96] – mostly hydrophobic amino acids. The ECDs are suggested to bind the main acceptor of cholesterol from the reverse cholesterol transport mediated by ABCA1, APOA1 [121]. Specifically the tunnel was proposed as (temporary) storage of cholesterol and/or phospholipids [96], which is also likely in terms of the cholesterol and phospholipid transporter ABCA7 [12], [13], [24], [34]. In comparison to the ABCA1 template, ECD1 of ABCA7 is shorter. This reflects in only two disulfide bonds compared to ABCA1, which contains three. The third disulfide bond formed between Cys75 and Cys309 (ABCA1) is not present in the homology model of ABCA7. On the other hand, the forth disulfide bond in ECD2 is present in both ABCA1 and ABCA7. Interestingly, the interruption in sequence of ABCA7 between the putative transmembrane tunnel/binding cavity and the ECD-tunnel hints to necessary conformational changes for the substrate/drug translocation process, which was also suggested for its functional counterpart, ABCA1 [96]. Fig. 4 (F) gives the schematic representation of the structural components of ABCA7.

The transporter ABCA1 selected as template for the homology modelling approach has been resolved in an ATP-unbound state [96]. This is of major importance, as this state allows for substrate/drug/modulator recognition/binding before ATP-dependent translocation of the substrate. This was demonstrated for other ABC transporters as well, *e.g.*, ABCB1 [102], ABCB2 [103], ABCB6 [122], ABCB11 [123], ABCC7 [124], ABCC8 [105], ABCG2 [106], and ABCG5/G8 [125], forming either an inward-facing [105], [106], [122], [123], [124], [125] or (drug-induced) occluded [102], [103] conformation. In contrast, most ABC transporters in the ATP-bound state are not able to recognize substrates, as shown for ABCB4 [104], as these form an outward-facing conformation. Only in exceptional cases an ATP-bound state was observed with an inward-facing conformation, for example, the mitochondrial ABC transporters ABCB8 [126] and ABCB10 [127]. In the case of the lysosomal ABC transporter ABCD4, the outward-facing conformation (bound to two molecules of ATP) was suggested as the actual state of acceptance of the substrate cobalamin [128]. These findings could be peculiarities of vesicular transporters [3], [4] and are likely to have no implications for other ABC transporters, such as ABCA7.

Interestingly, ABCA1 was found in a ‘(pseudo-)outward-facing’ conformation, which means that – unlike other ABC transporters in the ATP-unbound state [102], [103], [106], [122] – the MSDs seem not to be exposed to the cytosol for substrate/drug/modulator attraction and translocation. This structural feature was distinct from the other known type II ABC transporter, ABCG5/ABCG8 [117]. However, the found state for ABCA1 [96] was supported by the cryo-EM data of ABCA4 [97], [98], [99]. In addition, as most TMs are oriented toward the inter-membrane space – potentially to attract and bind cholesterol and phospholipids present within the membrane – a classical ‘inward-facing’ conformation seems unnecessary. In conclusion, this (pseudo-)outward-facing conformation appears not to be a peculiarity of type II transporters in general but of ABCA transporters in particular, and we were confident with the generated homology model to be in the most relevant conformational state for ligand binding to continue with molecular modelling studies.

### 2.3 Molecular docking

**Fig. 5:**
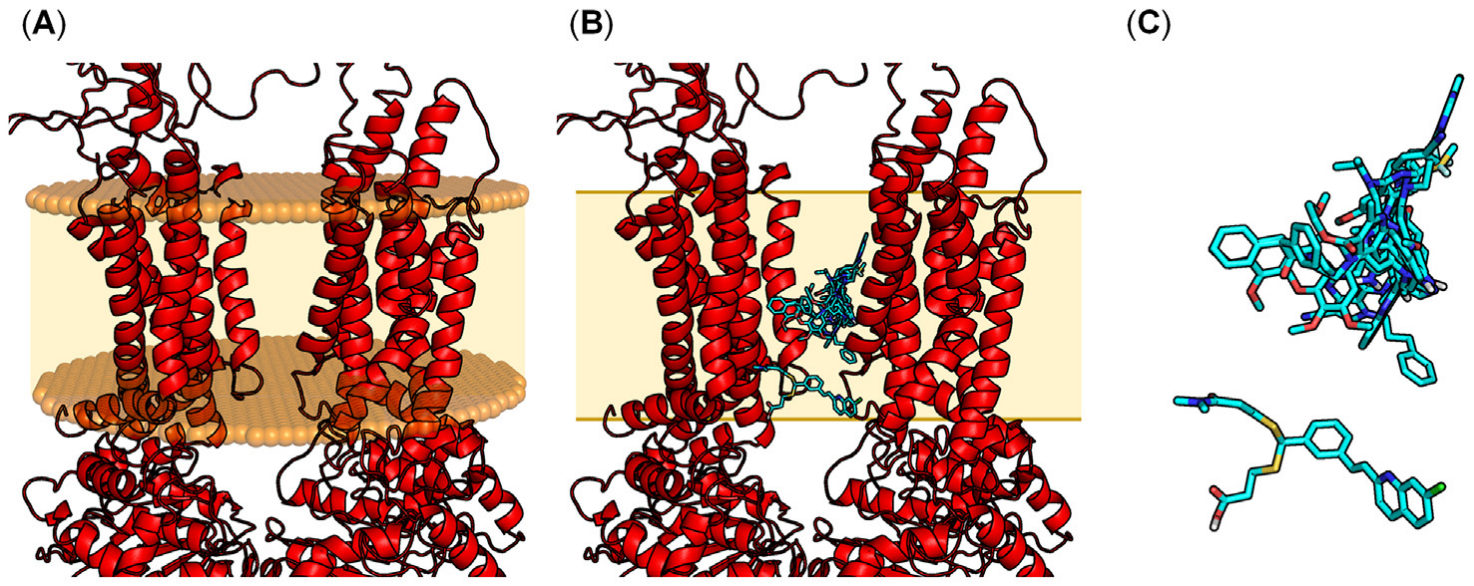
Blind docking using the herein described homology model of ABCA7 applying AutoDock [129]. (A) Space chosen for the blind docking experiments within the membrane bilayer indicated with brown balls and a light brown area. (B) Superimposed top ranking docking poses of the ten chosen pan-ABC transporter inhibitors **9**–**11**, **14**, **17**, and **22**–**26**[38], [45], [101], [134], [135], [136], [137] amongst the 50 docking poses generated by AutoDock [129] (colored cyan, stick representation) within the MSDs of the ABCA7 homology model. (C) Close-up of the superimposed top ranking poses of the docked molecules **9**–**11**, **14**, **17**, and **22**–**26**[38], [45], [101], [134], [135], [136], [137]. Nonpolar hydrogen atoms were omitted, and polar hydrogen, carbon, nitrogen, oxygen, as well as sulfur atoms were colored in silver white, cyan, blue, red, and dark yellow, respectively. (For interpretation of the references to colour in this figure legend, the reader is referred to the web version of this article.)

Directly interacting small-molecules of ABCA7 are unknown except for the substrates cholesterol and phospholipids [12], [13], [24], [34]. No genuine structural information of ABCA7 is available from which a potential binding site of its substrates could have been deduced. A lipid-filled gap could be identified in the recently announced cryo-EM structure of ABCA7 (PDB ID: 7KQC; PDB status: unreleased deposition withdrawn), however, neither the identity nor the binding region/site of these lipids could be determined [95]. A binding site for the most studied ABCA transporter ABCA1 has only been suggested [96], and only the very recently published ABCA4 cryo-EM structures outlined potential phospholipid and retinoid binding sites [97], [98]. However, a common or overlapping (multitarget) binding site amongst all ABC transporter superfamily members has recently been suggested acknowledging the multitarget affinity and potency of truly multitarget as well as focused pan-ABC transporter inhibitors (Table 1) [4], [38], [45]. These pan-ABC transporter inhibitors inhere the molecular-structural information for multitarget inhibition – and therefore the potential of multitarget exploration of under-studied ABC transporters [38], [45], such as ABCA7. Thus, we conducted blind docking studies considering the entire MSDs of the ABCA7 homology model using AutoDock [129]. The MSDs as docking space were particularly chosen because they cover the suggested/outlined binding sites in other ABCA transporters [96], [97], [98], which is generally supported by the structural information of other ABC transporters subfamilies [100], [101], [102], [103], [104], [105], [106], [107], [108], [109], [110], [111], [112], [113], [114]. Fig. 5 (A) outlines the focused space for the blind docking experiments.

In a first step, we assembled a list of 23 diverse, modern, and potent pan-ABC transporter inhibitors (**6**–**28**; Fig. 2; Table 1) covering the maximal structural diversity possible amongst known pan-ABC transporter inhibitors [38], [45], [101], [130], [131], [132], [133], [134], [135], [136], [137], [138], [139], [140]. From these 23 molecules, a first set of 10 diverse molecular representatives was chosen for the blind docking experiments (**9**–**11**, **14**, **17**, and **22**–**26**) [38], [45], [101], [134], [135], [136], [137] using AutoDock [129]. Compounds **9**–**10** were chosen because these two molecules are the most promiscuous known in terms of ABC transporter inhibition (9 [69], [70], [76], [85], [86], [87], [88], [89], [90] and 11 [69], [73], [75], [76], [85], [86], [91], [92], [93], [94] targets, respectively). Amongst the targets of compounds **9**–**10** is also another ABCA transporter, namely ABCA8 [85]. Compound **11** was taken due to its novelty and structural uniqueness [38]. Finally, compounds **14**, **17**, and **22**–**26** were taken due to their high multitarget potency, as these compounds are all class 7 molecules (IC_50 (ABCB1, ABCC1, and ABCG2)_<10 µM) [45], [101], [134], [135], [136], [137].

Fig. 5 (B) provides the top ranking docking poses of these 10 docked molecules **9**–**11**, **14**, **17**, and **22**–**26** [38], [45], [101], [134], [135], [136], [137] amongst the 50 docking poses generated for each molecule by AutoDock [129] in the ABCA7 homology model. The top ranking docking pose of each individual molecule can be found in Supplementary Fig. S3 (A)–(J), and Supplementary Table 1 provides the docking scores of the top ranking docking pose of each molecule obtained from AutoDock [129]. In addition, Supplementary Table 1 contains also the docking scores of the two phospholipids PL1 and PL2 that were found complexed in one of the ABCA4 cryo-EM structures [98]. These scores were better in comparison to the docked pan-ABC transporter inhibitors; however, this may be due to the very strong electrostatic interactions of the phosphate group and the amino acids, which did not occur in the case of pan-ABC transporter inhibitors. As can be seen from the docking poses, the molecular-structural orientation of the majority of the pan-ABC transporter modulators resembled or partially overlapped each other [Fig. 5 (C)]. The docking poses of the molecules largely bind between the two MSDs interacting with residues of TM1–2, TM5, TM7–TM8, and TM11, majorly through hydrophobic interactions, which is in agreement with the binding pocket identified for phospholipids in ABCA4 [97], [98]. The identified molecular-structural orientations of compounds **9**–**10**, **14**, **17**, and **22**–**26** [38], [45], [101], [134], [135], [136], [137] of the top ranking docking poses suggest common or partially overlapping pharmacophore features amongst these compounds, which was subsequently explored by applying pharmacophore modelling.

### 2.4 Pharmacophore modelling

**Fig. 6:**
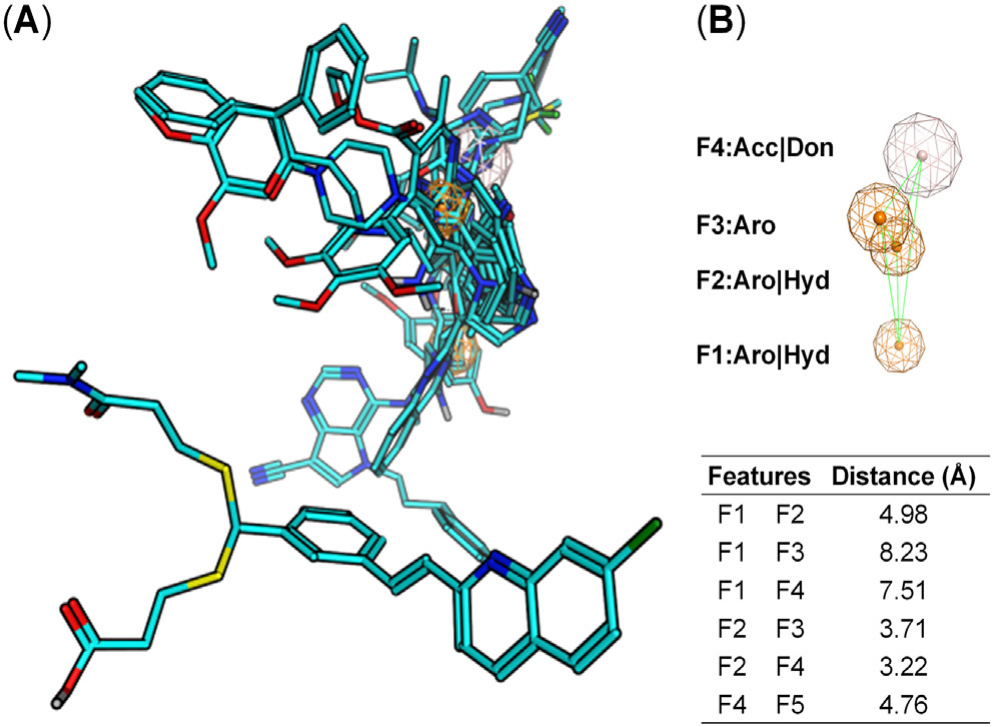
Pharmacophore model using the top ranking docking poses of compounds **9**–**11**, **14**, **17**, and **22**–**26**[38], [45], [101], [134], [135], [136], [137] obtained from AutoDock [129]. (A) Superimposed top ranking poses of the docked compounds **9**–**11**, **14**, **17**, and **22**–**26**[38], [45], [101], [134], [135], [136], [137] from which the four pharmacophore features F1–F4 could be deduced (colored cyan, stick representation). Nonpolar hydrogen atoms were omitted, and polar hydrogen, carbon, nitrogen, oxygen, as well as sulfur atoms were colored in silver white, cyan, blue, red, and dark yellow, respectively. (B) The four pharmacophore features F1–F2 (aromatic/hydrophobic), F3 (aromatic), and F4 (acceptor/donor) are depicted in orange (F1–F3) as well as silver (F4). The distances between the individual features are indicated as light green lines and are outlined in the table. (For interpretation of the references to colour in this figure legend, the reader is referred to the web version of this article.)

In order to explore common or overlapping pharmacophore features between pan-ABC transporter modulators, pharmacophore modelling was applied using MOE 2019.01 [118]. The model was generated by utilizing the top ranking docking poses of compounds **9**–**11**, **14**, **17**, and **22**–**26** [38], [45], [101], [134], [135], [136], [137] obtained from the molecular docking studies with AutoDock [129] [Fig. 5 (B)–(C)]. Fig. 6 (A)–(B) outlines the four identified pharmacophore features F1–F2 (aromatic/hydrophobic), F3 (aromatic), and F4 (acceptor/donor), as well as the distances between the individual features.

As the top ranking binding pose of a molecule is not necessarily the most relevant one, the other 49 binding poses generated from AutoDock [129] needed to be explored. This was accomplished by a pharmacophore search amongst the 50 docked poses of compounds **9**–**11**, **14**, **17**, and **22**–**26** [38], [45], [101], [134], [135], [136], [137] obtained from AutoDock [129] by screening against the four identified pharmacophore features F1–F4. Very interestingly, at least one of the 50 AutoDock-generated [129] conformations of each of the 10 molecules matched in full with the four identified pharmacophore features. The best fitting conformations of compounds **9**–**11**, **14**, **17**, and **22**–**26** [38], [45], [101], [134], [135], [136], [137] in terms of the pharmacophore features F1–F4 are shown in Supplementary Fig. S4 (A)–(J), and the root mean square distance (RMSD) of the best fitting docking poses of the 10 molecules in terms of the pharmacophore features F1–F4 with the respective docking scores are given in Supplementary Table 1.

### 2.5 Model validation

**Fig. 7:**
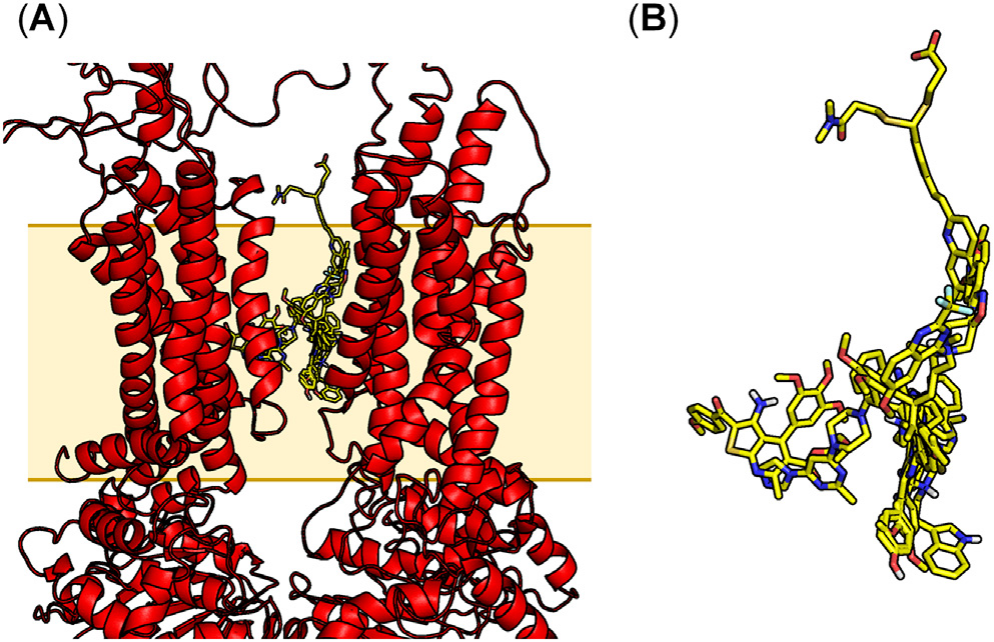
Blind docking using the herein described homology model of ABCA7 applying Glide [141], [142]. (A) Superimposed top ranking docking poses of the pan-ABC transporter inhibitors **9**–**11**, **14**, **17**, and **22**–**26**[38], [45], [101], [134], [135], [136], [137] amongst the 10 docking poses generated by Glide [141], [142] (colored yellow, stick representation) within the MSDs of the ABCA7 homology model. (B) Close-up of the superimposed top ranking poses of the docked molecules **9**–**11**, **14**, **17**, and **22**–**26**[38], [45], [101], [134], [135], [136], [137]. Nonpolar hydrogen atoms were omitted, and polar hydrogen, carbon, nitrogen, oxygen, as well as sulfur atoms were colored in silver white, yellow, red, blue, and dark yellow, respectively. (For interpretation of the references to colour in this figure legend, the reader is referred to the web version of this article.)

To further validate the generated pharmacophore model, we applied another docking tool, Glide [141], [142], implemented in Schrödinger 2020–3 [143], and the very same compounds **9**–**11**, **14**, **17**, and **22**–**26** [38], [45], [101], [134], [135], [136], [137] were docked. Fig. 7 demonstrates the top ranking poses of the docked molecules amongst the 10 docking poses generated by Glide [141], [142]. Supplementary Fig. S5 (A)–(J) provides each individual top ranking docking pose of the compounds, and Supplementary Table 2 provides the respective docking scores as well as the docking scores of the two phospholipids that were found complexed in one of the ABCA4 cryo-EM structures [98]. With respect to Glide [141], [142], the docking scores of the phospholipids were comparable to the docking scores of the pan-ABC transporter inhibitors.

Similar to AutoDock [129], the top ranking docking poses generated from Glide [141], [142] do not need to be the most relevant ones in terms of the binding of the molecules. Thus, in our next step, the 10 resultant docking poses of each of the compounds **9**–**11**, **14**, **17**, and **22**–**26** [38], [45], [101], [134], [135], [136], [137] obtained from Glide [141], [142] were screened against the established pharmacophore model [Fig. 6 (A)–(B)]. The best fitting conformations of compounds **9**–**11**, **14**, **17**, and **22**–**26** [38], [45], [101], [134], [135], [136], [137] in terms of the pharmacophore features F1–F4 are shown in Supplementary Fig. S6 (A)–(J), and the RMSD of the best fitting docking poses of the 10 molecules in terms of the pharmacophore features F1–F4 with the respective docking scores are given in Supplementary Table 2.

Strikingly, only compound **10** did not fully match the searched pharmacophore features F1–F4, while at least one of the 10 generated docking poses from Glide [141], [142] of each of the compounds **9**, **11**, **14**, **17**, and **22**–**26** [38], [45], [101], [134], [135], [136], [137] were identified as hit in terms of the four pharmacophore features F1–F4 [Fig. 6 (A)–(B)]. The reason for this may lie in the presence of an acid function, which is unique amongst the docked compounds **9**, **11**, **14**, **17**, and **22**–**26** [38], [45], [101], [134], [135], [136], [137] and forms different possible interactions.

Strikingly, pharmacophore modelling taking the top ranking docking poses generated from Glide [141], [142] into account resulted in similar four pharmacophore features [F1–F3 (aromatic/hydrophobic) and F4 (acceptor/donor); Supplementary Fig. S7], which were reflected in full in both the generated poses from Autodock [129] (Supplementary Fig. S8) and Glide [141], [142] (Supplementary Fig. S9) of compounds **9**, **11**, **14**, **17**, and **22**–**26** [38], [45], [101], [134], [135], [136], [137]. The elucidated pharmacophore features identified from Glide [141], [142] are closer to each other than the features from AutoDock [129]. Although no direct overlap could be observed between the two pharmacophore models, the identified features (particularly aromatic/hydrophobic) seem to complement each other. These results suggest that the specified pharmacophore model and the respective binding site could be explored as common or overlapping binding site of pan-ABC transporter inhibitors.

**Fig. 8:**
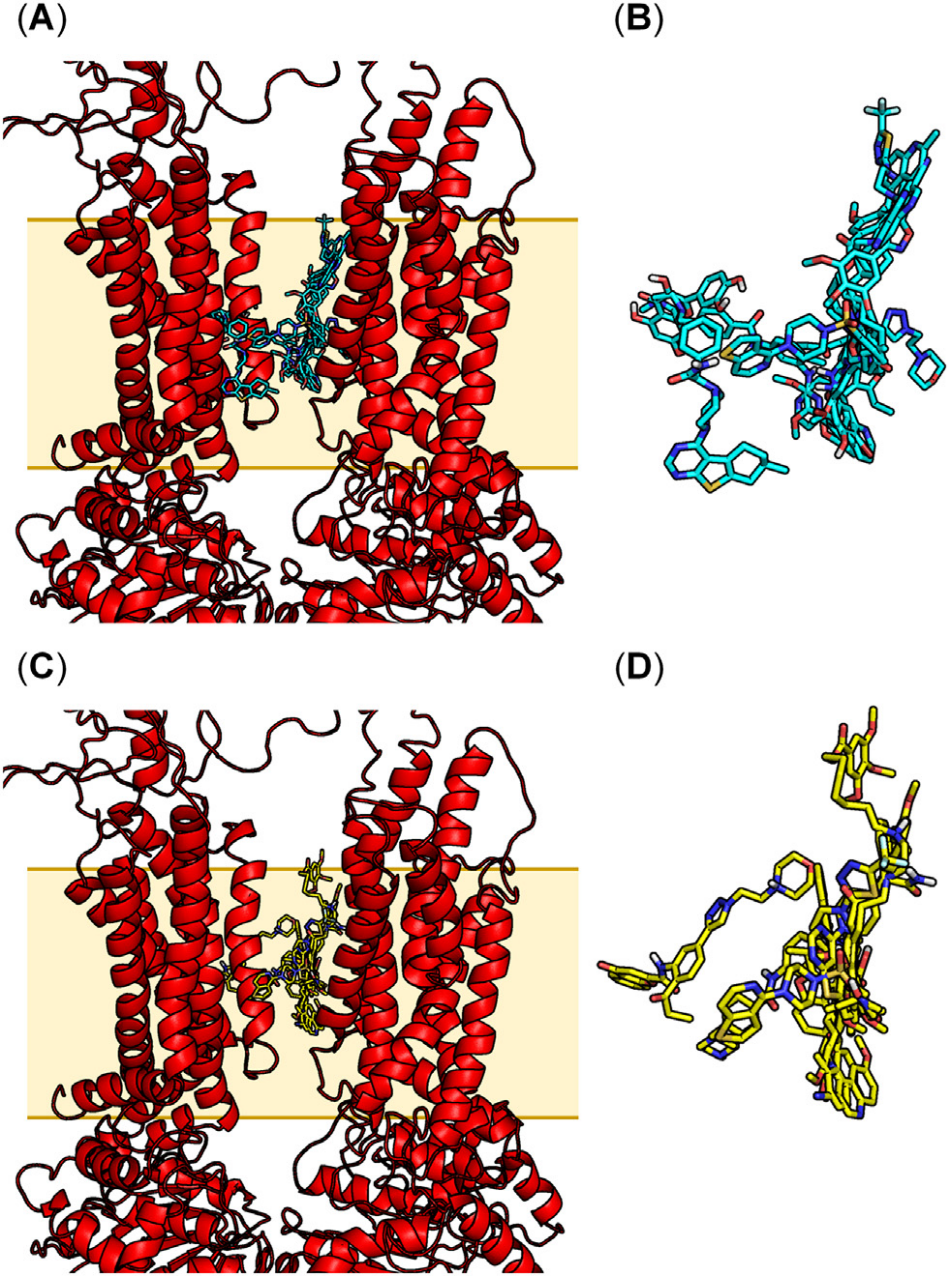
Extended docking experiments with compounds **6**–**8**, **12**–**13**, **15**–**16**, **18**–**21**, and **27**–**28**[45], [130], [131], [132], [133], [138], [139], [140] for model validation purposes. (A) Superimposed top ranking docking poses of the compounds obtained from AutoDock [129]. (B) Close-up of the superimposed top ranking poses of the docked molecules. (C) Superimposed top ranking docking poses of the compounds obtained from Glide [141], [142]. (D) Close-up of the superimposed top ranking poses of the docked molecules. The compounds are colored in cyan (A)–(B) as well as yellow (C)–(D) and are shown in stick representation. Nonpolar hydrogen atoms were omitted, and polar hydrogen, carbon, nitrogen, oxygen, as well as sulfur atoms were colored in silver white, cyan/yellow, blue, red, and dark yellow, respectively. (For interpretation of the references to colour in this figure legend, the reader is referred to the web version of this article.)

In the next step of model validation, a second set of 13 pan-ABC transporter inhibitors, compounds **6**–**8**, **12**–**13**, **15**–**16**, **18**–**21**, and **27**–**28** [45], [130], [131], [132], [133], [138], [139], [140] (Fig. 2; Table 1), was docked with both AutoDock [129] and Glide [141], [142]. The resultant top ranking docking poses are shown in Fig. 8 (A)–(D). Supplementary Figs. S10 (A)–(M) and S11 (A)–(M) provide the individual top ranking docking poses of the compounds applying either AutoDock [129] or Glide [141], [142], and the docking scores are provided in Supplementary Tables 3 (AutoDock [129]) and 4 (Glide [141], [142]). Furthermore, conformers of these 13 molecules were generated using the conformer generation tool implemented in MOE 2019.01 [118].

The 50 and 10 docked poses generated from AutoDock [129] and Glide [141], [142], respectively, as well as the 2,445 conformers generated with the conformer generation tool (MOE 2019.01) [118] for each molecule were screened against the developed pharmacophore model generated from the top ranking docking poses as obtained from Autodock [149] as described above [Fig. 6 (A)–(B)]. From these 13 candidates, 9 (AutoDock [129]), 7 (Glide [141], [142]), and 10 [conformer generation tool (MOE 2019.01) [118]] molecules were identified as hit [118], [129], [141], [142]. Supplementary Figs. S12 (A)–(I), S13 (A)–(G), and S14 (A)–(J) provide the best fitting conformations of compounds **6**–**8**, **12**–**13**, **15**–**16**, **18**–**21**, and **27**–**28** [45], [130], [131], [132], [133], [138], [139], [140] in terms of the pharmacophore features F1–F4 [Fig. 6 (A)–(B)], and the RMSD of the best fitting docking poses of the molecules in terms of the pharmacophore features F1–F4 with the respective docking scores are given in Supplementary Tables 3 (AutoDock [129]) and 4 (Glide [141], [142]). In essence, 7 compounds (**12**–**13, 15**–**16, 18, 21, 27** [45], [130], [133], [138]) identified from these three approaches reflected the four pharmacophore features F1–F4 in full. On the other hand, compounds **6**–**8**, **19**–**20**, and **27** matched only in part with the four pharmacophore features F1–F4. As different binding sites and (sub)pockets of ABC transporter ligands generally exist [47], [98], [101], the proposed binding site of multitarget inhibitors is potentially favorable for some of the pan-ABC transporter inhibitors and results in strong binding affinities. Alternatively, weaker affinities may result for other molecules that mutually and/or additionally bind to (a) distinct binding site(s) as they bear only a part of the ‘necessary’ features.

### 2.6 Putative binding site

**Fig. 9:**
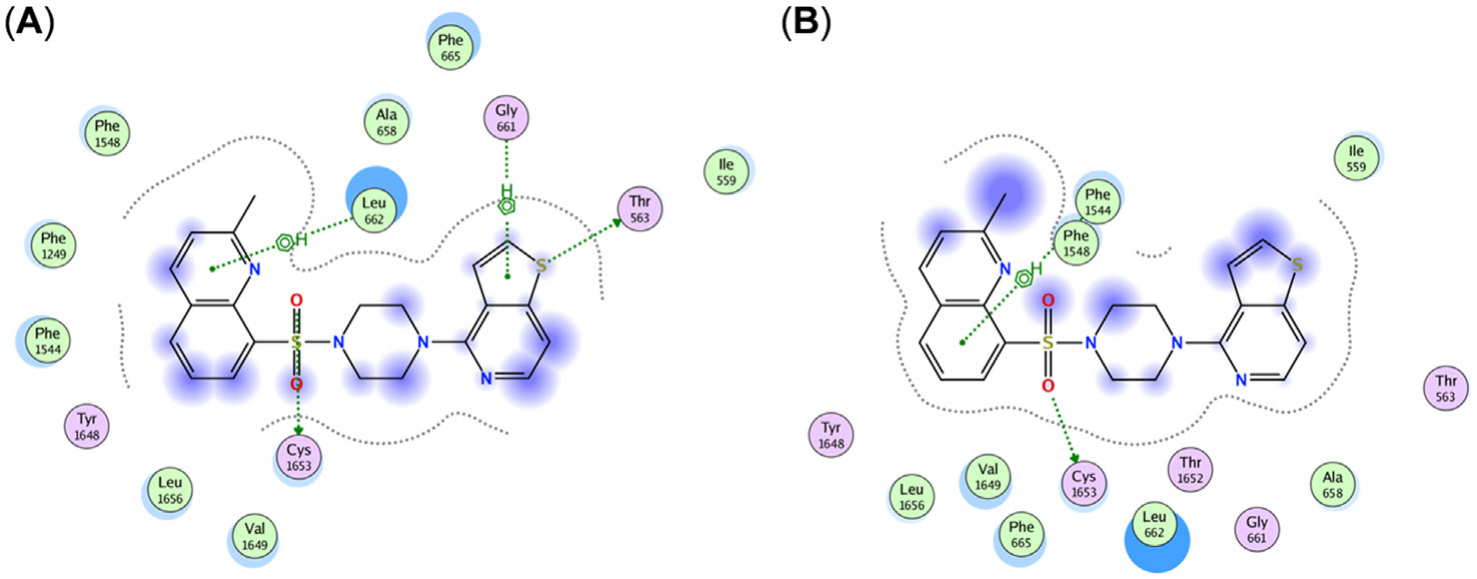
2D interaction diagram of the top ranking docking poses of compound **15** and possible strong interactions with amino acids of the multitarget binding site generated by PLIF implemented in MOE 2019.01 [118]. (A) Possible interactions obtained from AutoDock [129] formed with phenylalanine 1544 and cysteine 1653. (B) Possible interactions obtained from Glide [141], [142] formed with leucine 662 and cysteine 1653. Hydrophobic and polar amino acids are shown in green circles and purple circles, respectively. Interactions with the amino acids are depicted as green dotted arrows (side chain acceptor) or green dotted lines (aromatic). (For interpretation of the references to colour in this figure legend, the reader is referred to the web version of this article.)

In the last step, we analyzed in detail the putative interactions of the 23 compounds with the amino acids in the binding pocket of the multitarget binding site. For this, we used the protein–ligand interaction fingerprint (PLIF) analysis tool implemented in MOE 2019.01 [118]. Fig. 9 provides the 2D interaction diagram of a representative compound, **15**, in its top ranking docking pose as obtained from docking with AutoDock [129] (A) and Glide [141], [142] (B). Supplementary Figs. S15 (A)–(J), S16 (A)–(J), S17 (A)–(M), and S18 (A)–(M) show the putative interactions of all evaluated 23 molecules in their top ranking docking poses as obtained from AutoDock [129] and Glide [141], [142].

**Fig. 10:**
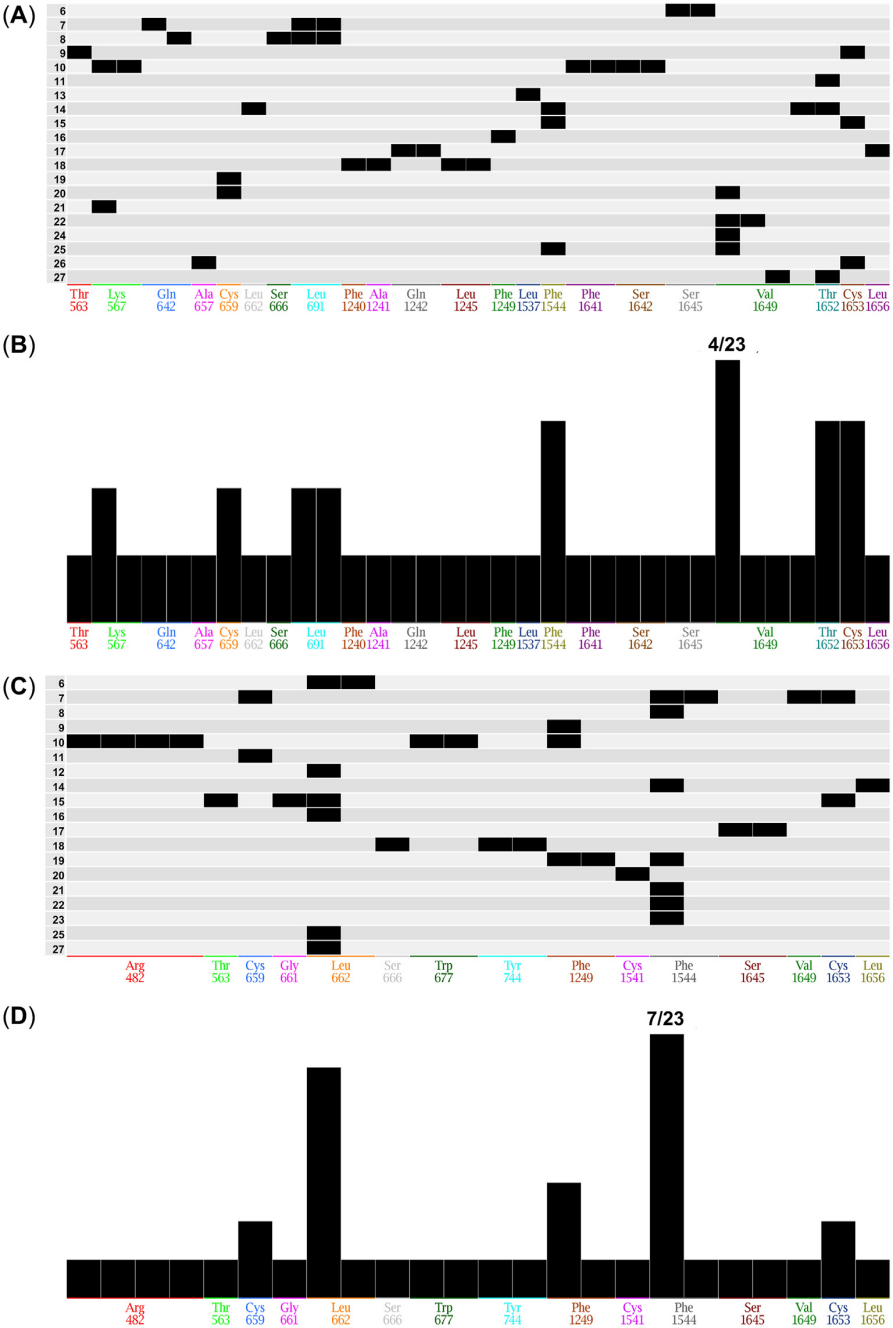
Distribution of the 23 docked compounds that may form possible interactions with amino acids in the MSDs of the generated homology model of ABCA7. (A) Bar code diagram and (B) population of the 23 docked compounds as obtained from AutoDock [129]). (C) Bar code diagram and (D) population of the 23 docked compounds as obtained from Glide [141], [142]. Single or multiple interactions are indicated with single and multiple columns, respectively. The respective engaged compound is indicated in the bar code diagram.

Compound **15** possibly formed strong hydrophobic interactions with leucine 662 and phenylalanine 1544 with its quinoline basic scaffold as demonstrated in AutoDock [129] and Glide [141], [142], respectively. In addition, both docking tools revealed cysteine 1653 as an important amino acid that possibly interacts with the sulfone of compound **15**. Both the hydrophobic and acceptor interaction of the quinoline and sulfone substructures, respectively, are two major parts of the developed pharmacophore models [Fig. 6 (B) and Supplementary Fig. S7]. These three amino acids are of importance as they are shared as interacting amino acids amongst several selected compounds and are highlighted in yellow in Supplementary Fig. S1. The bar code diagram in Fig. 10 provides insights into the most frequently occurring interactions with amino acids and the 23 docked compounds. Besides the mentioned amino acids, AudoDock [129] revealed also cysteine 659, valine 1649, and threonine 1652 as putatively important. These amino acids are located in TM5, TM8, and TM11 and are also highlighted in yellow in Supplementary Fig. S1. None of these six amino acids appeared in a critical region as outlined in Supplementary Fig. S2. Interestingly, cysteine 659, phenylalanine 1544, and valine 1649 are highly conserved amongst ABCA1 [96], ABCA4 [98], [99], and ABCA7 and may form the backbone of the multitarget binding site. A further detailed analysis of these amino acids with different methodologies is in need to validate the found results.

## 3 Conclusions

Multitarget agents have generally received increased interest over the last years, not only in terms of polypharmacology [46], [144], [145], [146] but also in terms of exploration of under-studied pharmacological targets [38], [44], [45]. Most members of the ABC transporter superfamily are considered as under-studied and cannot be addressed by small-molecules [38], [44], [45]. Except for ABCA1, which is a so-called ‘less-studied’ ABC transporter [38] with only 14 known inhibitors of this transport protein [147], [148], [149], [150], all members of the ABCA subfamily are under-studied. Half of the ABCA transporter sub-family is associated with AD [13], [19], [24], [25], [26], [27], [28], [34], and defective ABCA7 in particular has statistically been proven to contribute to AD development and/or progression [13], [24], [25], [34], [35], [37]. The lack of directly interfering small-molecules hinders research in developing molecular tools to functionally study this particularly important ABC transporter, hampering the development of novel and innovative AD diagnostics and therapeutics.

This lack of small-molecule interactors – of under-studied ABC transporters in general, and ABCA7 in particular – can be explained by three aspects: (i) the lack of multitargeting agents that share affinities to several ABC transporters of different sub-families, in particular ABCA transporters. The development of multitarget inhibitors has only very recently been focused [38], [44], [45], which limits the number of molecules. To this date, only 140 focused pan-ABC transporter inhibitors exist [38], [44], [45], [130], [151], [152], and non-focused pan-ABC transporter inhibitors (independent of targeted transporters) with at least 3 targeted transporters have only been described for roughly 120 compounds. Generally, modulators of ABCA transporters are less known, only ABCA1 (14 inhibitors [147], [148], [149], [150]) and ABCA8 (7 inhibitors [85], [153]) can be addressed by inhibitors, and small-molecule activators are unknown; (ii) the lack of structural information of ABCA7 in particular and ABCA transporters in general from which information can be deduced to design novel directly-interacting agents. The four cryo-EM structures of ABCA1 [96] and ABCA4 [97], [98], [99] have been reported rather recently in 2017 and 2021, and hence, have not been used for the design of novel agents yet. The already in 2020 announced [95] cryo-EM structure of ABCA7 (PDB ID: 7KQC; PDB status: unreleased deposition withdrawn) is to this date not publicly available, and thus, cannot be used for structural analyses; and (iii) the lack of standardized HTS assays to monitor the function of ABCA7 and to determine the inhibiting (or activating) property of compounds, as this is the case, for example, for the well-studied ABC transporters [38] ABCB1, ABCC1, and ABCG2 [154]. Assays using labelled tracers of ABCA transporter (particularly ABCA1) function, mostly NBD- [155] or BODIPY-labeled [156] cholesterol, indeed exist, however, have to this day not led to small-molecule inhibitors or activators. One reason is that these assays have primarily been developed to evaluate inducers of ABCA1 [155]. Another reason may be that the used tracers (NBD- [155] or BODIPY-cholesterol [156]) are derivatives of the rather unspecific and ubiquitously present intrinsic substrate cholesterol. Taken together, these three obstacles hinder the targeted development of selective and potent agents for under-studied ABC transporters involved in human diseases, such as ABCA7. However, the present study in combination with recent advances provides an initial hypothesis and further evaluation of these three stated problems.

First of all, computer-aided pattern analysis (‘C@PA’) has very recently been invented to rationally discover and design novel multitargeting pan-ABC transporter inhibitors to obtain potential ligands for under-studied ABC transporters [38], [44], [45]. Hereby, compounds **11**–**16** [38], [45] have been discovered as moderately potent class 7 [38], [44], [45] and semi class 7 (IC_50_ ≤ 15 µM) [56] molecules. These molecules represent a novel generation of broad-spectrum ligands of the well-studied ABC transporters [38] ABCB1, ABCC1, and ABCG2. These pan-ABC transporter inhibitors have not been evaluated toward other transporters yet, however, might be the key as transition molecules for the generation of selective and potent agents for under-studied ABC transporters. A common binding site of pan-ABC transporter inhibitors has been proposed [4], [38], [44], [45], which was focused in the present study and is subject to current research.

Second, to explore this potential multitarget binding site, the complete available structural information of human ABCA1 [96] and ABCA4 [98], [99] has been used and extended for the first time to the target of interest of the present study, ABCA7. The generated homology model of ABCA7 could be applied for blind docking experiments using diverse truly multitarget [69], [70], [73], [74], [75], [76], [77], [78], [79], [80], [81], [82], [83], [84], [85], [86], [87], [88], [89], [90], [91], [92], [93], [94] as well as focused [38], [45], [101], [130], [131], [132], [133], [134], [135], [136], [137], [138], [139], [140] pan-ABC transporter inhibitors with two different docking tools, Autodock [129] and Glide [141], [142]. Supported by pharmacophore modelling, the present report suggests that 16 (**9**, **11**–**18, 21**–**27**) [38], [45], [101], [130], [133], [134], [135], [136], [137], [138]) of the 23 compounds had consensus features that could be attributed to a common or partially overlapping multitarget binding site. These features F1–F4 (Fig. 6) may be essential to interact with the amino acids in the binding site, specifically cysteine 659, leucin 662, phenylalanine 1544, valine 1649, threonine 1652, and cysteine 1653 (Figs. 10 and 11 as well as Supplementary Fig. S1). The discovery of the consensus features amongst these structurally diverse pan-ABC transporter inhibitors **9**, **11**–**18**, **21**–**27** [38], [45], [101], [130], [133], [134], [135], [136], [137], [138]) in two different *in silico* tools and systematic cross-validation strongly supports the postulated multitarget binding site amongst ABC transporters of different sub-families, which may generally overlap with the binding site of ‘regular’ substrates of the respective transporter (Supplementary Fig. S19).

Amongst these 16 molecules is the truly-multitarget inhibitor **9**, which targets 9 different ABC transporters of 4 different sub-families. [69], [70], [76], [85], [86], [87], [88], [89], [90]. Compound **9** is of particular interest, not only due to its truly multitargeting nature in terms of ABC transporters in general, but also because it was demonstrated to activate ABCC1-mediated transport of the ABCC1 substrate glutathione (GSH) [69]. Defective ABCA7 calls for modulators that increase its transport capability. Generally, activators of ABC transporters have been found before [47], [48], [49], [51], [52], [53], [54], [55]. However, the particular association of an ABC transporter activator with pan-ABC transporter inhibition opens a new perspective on multitargeting in general. Besides inhibitory activities and physicochemical properties, the mode of modulation, in particular the activating nature of compounds, can supplement the newly invented C@PA [38], [44], [45] methodology, expanding bioactivity space of small-molecules and associating their molecular-structural patterns with increase of functional ABC transporter activity. Amongst the known focused pan-ABC transporter inhibitors [38], [45], [130], [151], [152], molecular-structural patterns can be found that have already been associated with activation of ABC transporters, such as tetrahydroisoquinoline [48], pyrrolopyrimidine [49], and purine [49].

While these aspects above were mostly relevant for therapeutic drug development, the discovery of inhibitors of ABCA7 by addressing the multitarget binding site and their simultaneous derivatization to selective agents would allow for *in vivo* imaging of ABCA7. Imaging of ABC transporters of the BBB has already been accomplished before [63], and the results of the present study found the basis for PET tracer development of other, yet under-studied ABC transporters involved in neurological disorders like AD, as for example, ABCA7.

The results presented in this report have broad implications for addressing other under-studied ABC transporters [38] as well as their exploration and exploitation as potential pharmacological drug targets to tackle major human diseases. Specifically the combination of both ligand-based pattern analysis and structure-based docking studies may provide novel, innovative, and effective drugs for the curation of human diseases in general.

## 4 Experimental section

### 4.1 Sequence alignment and homology model

The sequences of the human ABC transporters ABCA1 (UniProt ID: O95477), ABCA4 (UniProt ID: P78363), and ABCA7 (Uniprot ID: Q8IZY2) were downloaded from Uniprot Knowledgebase [157]. The sequence identity and similarity between the three ABCA transporter subtypes were analyzed using Align/Superpose module implemented in Molecular Operating Environment (MOE) 2019.01 [118]. The sequence similarity of ABCA7 was 52.8% and 48.2% compared to ABCA1 and ABCA4, respectively. The cryo-EM structures of ABCA1 (PDB ID: 5XJY [96]) and ABCA4 (PDB ID: 7E7I [98]; PDB ID: 7LKP [99]; PDB ID: 7M1Q [97]) were downloaded from the RCSB protein data bank (PDB) [158]. For homology modelling purposes, the ABCA1 cryo-EM structure was selected due to its functionally and structurally closer similarity to ABCA7. As an initial step, the co-crystallized sugar molecules of ABCA1 have been removed. Ionization and hydrogen atoms were added using the Protonate-3D tool implemented in MOE 2019.01 [118], and the structures were subsequently energy-minimized by keeping the heavy atoms fixed at their crystallographic positions applying the Amber99 force field [159] until the root-mean-square of the conjugate gradient was less than 0.05 kcal·mol^−1^ · Å^−1^. A total of 250 models were generated using the GB/VI scoring function with a gradient limit of 0.5.

### 4.2 Molecule preparation and conformer generation

All 2D representation of the molecules were drawn using ChemDraw version 20.1.1. and optimized using the builder module implemented in MOE 2019.01. The hydrogen atoms were added, energy minimized by applying the AMBER10:EHT forcefield. The conformers of these molecules were generated using the conformer generation tool by selecting the stochastic search method implemented in MOE 2019.01 [118]. The default parameters were applied for the conformational search with a maximum limit of 10,000. The lowest energy conformer obtained were selected for further preparation in the respective docking tools. Finally for the 13 pan-ABC transporter inhibitors **6**–**8**, **12**–**13**, **15**–**16**, **18**–**21**, and **27**–**28** [45], [130], [131], [132], [133], [138], [139], [140] selected for validation the conformers generated were utilized for the analysis. In total, 2,445 conformers of the 13 molecules were generated.

### 4.3 Molecular docking

#### 4.3.1 PSO@AutoDock

The generated homology model of the human ABCA7 was used for the docking procedure applying the particle swarm optimization (PSO) tool, PSO@AutoDock [129], implemented in AutoDock 4.2 [160]. The AutoDockTools package was employed to generate the docking input files and to analyze the docking results. The search algorithm, *var*CPSO-ls [129], from PSO@AutoDock [129] implemented in AutoDock4.2 [160] was employed for docking calculations. Three-dimensional energy scoring grids of 0.375 Å resolution and a dimension of 120 Å × 120 Å × 120 Å were computed. The grids were centered based on the two MSDs of the ABCA7 homology model. The parameters of the *var*CPSO-ls algorithm [129], cognitive coefficient (c1) and social coefficient (c2), were set to 6.05 and the swarm size as 60 individual particles. The default values were applied for the remaining parameters of the algorithm. A total of 50 independent docking calculations were performed by setting the termination criteria as 50,000 evaluation steps. The docked poses obtained from docking studies for the selected compounds were explored by visual inspection and the putative binding poses were selected.

#### 4.3.2 Glide

As a in initial step, the homology model of human ABCA7 was prepared using the Protein Preparation Wizard module implemented in Schrödinger 2020–3 [143]. The preparation of the protein included three steps: (i) assigning bond orders; (ii) adding the missing hydrogen atoms; (iii) and generating het states with the support of Epik module, a tool which can predict the protonation state of the protein structure at the physiological pH of 7.4. Then in the next step, assignment of H-bond and optimization by using the PROPKA module implement in Schrödinger 2020–3. Finally, the protein is minimized by setrestraining the heavy atoms with a maximum RMSD value of 0.30 Å using the Liquid Simulations Version 3 (OPLS3) force field [161].

The selected ligands were prepared using LigPrep module implemented in Schrödinger 2020–3 [143] and were optimized with the OPLS force field [161]. The grid files required for the docking procedure were prepared using the Receptor Grid Generation panel of Glide [141], [142] with grid points calculated enclosing a box covering the entire transmembrane region similar to PSO@AutoDock [129]. After grid preparation, the selected molecules were docked into the generated transporter grids using Glide XP (extra precision) docking. For the docking procedure, the default parameter settings were applied. A total of 10 independent docking calculations were performed.

### 4.4 Pharmacophore modelling

The top ranking docking poses of the ten selected molecules from PSO@AutoDock [129] were considered for generating the pharmacophore model. The pharmacophore model was established using the pharmacophore elucidator module implemented in MOE 2019.01 [118]. In this module, the pharmacophore features were automatically calculated using the consensus pharmacophore function. This function clusters the features into potential pharmacophore features which are more conserved than a tolerance and threshold distance of 1.5 Å and the threshold of 30%, respectively.

## CRediT authorship contribution statement

**Vigneshwaran Namasivayam:** Conceptualization, Methodology, Validation, Formal analysis, Investigation, Data curation, Writing – original draft, Writing – review & editing, Visualization, Project administration. **Katja Stefan:** Writing – review & editing, Funding acquisition. **Jens Pahnke:** Resources, Writing – review & editing, Project administration, Funding acquisition. **Sven Marcel Stefan:** Conceptualization, Data curation, Writing – original draft, Writing – review & editing, Visualization, Project administration, Funding acquisition.

## Declaration of Competing Interest

The authors declare that they have no known competing financial interests or personal relationships that could have appeared to influence the work reported in this paper.
