# Supplementary material for: Binding mode analysis of ABCA7 for the prediction of novel Alzheimer's disease therapeutics
Source: Comput Struct Biotechnol J. 2021 Nov 27;19:6490–504. doi: 10.1016/j.csbj.2021.11.035 (PMC8666613; doi:10.1016/j.csbj.2021.11.035)
Supplement: Supplementary data 1 [file mmc1.pdf]

Binding mode analysis of ABCA7 for the prediction of novel Alzheimer's disease therapeutics

Vigneshwaran Namasivayam[a], Katja Stefan,[b] Jens Pahnke*[,b,c,d], Sven Marcel Stefan*[,b]

[a] Department of Pharmaceutical and Cellbiological Chemistry, Pharmaceutical Institute, University of Bonn, An der Immenburg 4, 53121 Bonn, Germany

[b] Department of Pathology, Section of Neuropathology, Translational Neurodegeneration Research and Neuropathology Lab, University of Oslo and Oslo University Hospital, Sognsvannsveien 20, 0372 Oslo, Norway

[c] LIED, University of Lübeck, Ratzeburger Allee 160, 23538 Lübeck, Germany

[d] Department of Pharmacology, Faculty of Medicine, University of Latvia, Jelgavas iela 1, 1004 Rīga, Latvia

* Corresponding Authors: Sven Marcel Stefan (s.m.stefan@medisin.uio.no)
Phone: +47 230 71468
Jens Pahnke (jens.pahnke@medisin.uio.no);
www.pahnkelab.eu; Phone: +47 230 71466

Supplementary Material

Supplementary Table 1. Docking scores of the top ranking poses of the 10 docked pan-ABC transporter inhibitors **9**–**10**, **11**, **14**, **17**, and **22**–**26** [38, 45, 101, 134-137] as well as the two phospholipids (PL1 and PL2) discovered in a complex with ABCA4 [98] using the homology model of ABCA7 applying the homology model of ABCA7 using AutoDock [129] as well as the RMSD values and the docking scores of the best fitting conformation of compounds **9**–**10**, **11**, **14**, **17**, and **22**–**26** [38, 45, 101, 134-137] out of the 50 generated docking poses obtained from AutoDock [129] in terms of the four pharmacophore features F1–F4 obtained from the top ranking docking poses as generated from Autodock [129].

Compd. No.	Docking Score Top Ranking Pose	RMSD Pharmacophore Model	Docking Score Best Fitting Pose
**9**	-4.52	0.44	-1.84
**10**	-5.97	0.66	-4.41
**11**	-5.89	0.40	-5.89
**14**	-4.69	0.68	-3.56
**17**	-6.50	0.54	-5.77
**22**	-6.71	0.52	-3.84
**23**	-6.93	0.81	-6.05
**24**	-5.89	0.78	-4.78
**25**	-6.67	0.64	-5.63
**26**	-3.84	0.38	-3.44
**PL1**	-15.0	-	-
**PL2**	-16.7	-	-

Supplementary Table 2. Docking scores of the top ranking poses of the 10 docked pan-ABC transporter inhibitors **9**–**10**, **11**, **14**, **17**, and **22**–**26** [38, 45, 101, 134-137] as well as the two phospholipids (PL1 and PL2) discovered in a complex with ABCA4 [98] using the homology model of ABCA7 applying the homology model of ABCA7 using Glide [141, 142], as well as the RMSD values and the docking scores of the best fitting conformation of compounds **9**, **11**, **14**, **17**, and **22**–**26** [38, 45, 101, 134-137] out of the 10 generated docking poses obtained from Glide [141, 142] in terms of the four pharmacophore features F1–F4 obtained from the top ranking docking poses as generated from Autodock [129].

Compd. No.	Docking Score Top Ranking Pose	RMSD Pharmacophore Model	Docking Score Best Fitting Pose
**9**	-6.18	0.85	-6.17
**10**	-7.49	-	-
**11**	-4.47	0.83	-4.47
**14**	-4.22	0.78	-4.22
**17**	-6.99	0.65	-6.99
**22**	-6.84	0.59	-6.12
**23**	-4.66	0.84	-4.04
**24**	-6.14	0.77	-6.14
**25**	-6.00	0.68	-5.60
**26**	-3.62	0.65	-3.62
**PL1**	-7.18	-	-
**PL2**	-4.16	-	-

Supplementary Table 3. Docking scores of the top ranking poses of the 13 docked pan-ABC transporter inhibitors **6–8**, **12–13**, **15–16**, **18–21**, **27**, and **28** [45, 130-133, 138-140] in the homology model of ABCA7 using AutoDock [129], the RMSD values and the docking scores of the best fitting conformation of compounds **12–13**, **15–16**, **18–21**, and **27** [45, 130-133, 138] out of the 50 generated docking poses obtained from AutoDock [129] in terms of the four pharmacophore features F1–F4 obtained from the top ranking docking poses as generated from Autodock [129], as well as the RMSD values of the conformers of compounds **7**, **12**, **13**, **15–16**, **18**, **20–21**, **27**, and **28** [57, 125, 127-128, 133-135] obtained from the conformer generation tool implemented in MOE 2019.01 [118] in terms of the four pharmacophore features F1–F4 obtained from the top ranking docking poses as generated from Autodock [129].

Compd. No.	Docking Score Top Ranking Pose	RMSD Pharmacophore Model	Docking Score Best Fitting Pose	RMSD Conformer
**6**	-6.07	-	-	-
**7**	-5.36	-	-	0.61
**8**	-4.90	-	-	-
**12**	-6.10	0.44	-5.20	0.77
**13**	-6.50	0.64	-6.50	0.55
**15**	-6.96	0.44	-6.75	0.39
**16**	-6.66	0.69	-4.98	0.62
**18**	-5.99	0.48	-3.15	0.45
**19**	-6.79	0.82	-5.91	-
**20**	-2.94	0.44	-1.54	0.30
**21**	-5.77	0.66	-5.69	0.44
**27**	-6.07	0.60	-5.90	0.62
**28**	-6.63	-	-	0.58

Supplementary Table 4. Docking scores of the top ranking poses of the 13 docked pan-ABC transporter inhibitors **6**–**8**, **12**–**13**, **15**–**16**, **18**–**21**, **27**, and **28** [45, 130-133, 138-140] in the homology model of ABCA7 using Glide [141, 142], as well as the RMSD values and the docking scores of the best fitting conformation of compounds **12**–**13**, **15**–**16**, **18**, **21**, and **27** [45, 130, 133, 138] out of the 10 generated docking poses obtained from Glide [141, 142] in terms of the four pharmacophore features F1–F4 obtained from the top ranking docking poses as generated from Autodock [129].

Compd. No.	Docking Score Top Ranking Pose	RMSD Pharmacophore Model	Docking Score Best Fitting Pose
**6**	-4.33	-	-
**7**	-4.98	-	-
**8**	-6.93	-	-
**12**	-5.07	0.75	-5.07
**13**	-5.83	0.75	-2.93
**15**	-5.38	0.56	-0.92
**16**	-5.43	0.76	-5.43
**18**	-3.90	0.51	-3.90
**19**	-5.01	-	-
**20**	-1.77	-	-
**21**	-5.87	0.68	-4.76
**27**	-5.66	0.81	-5.66

```
                                      IH1                  TM1                 ECD1
SP|O95477|ABCA1_HUMAN  MACWPQLRLLLWKNLTFRRRQTCQLLLEVAWPLFIFLILISVRLSYPPYEQHECHFPNKA 60
SP|P78363|ABCA4_HUMAN  MGFVRQIQLLLWKNWTLRKRQKIRFVVELVWPLSLFLVLIWLRNANPLYSHHECHFPNKA 60
SP|Q8IZY2|ABCA7_HUMAN  MAFWTQLMLLLWKNFMYRRRQPVQLLVELLWPLFLFFILVAVRHSHPPLEHHECHFPNKP 60
                       *.   *: ******   *:**  ::::*: *** :*::*: :* : *  .:********

                                                   ECD1
SP|O95477|ABCA1_HUMAN  MPSAGTLPWVQGIICNANNPCFRYPTPGEAPGVVGNFNKSIVARLFSDARRLLLYSQKDT 120
SP|P78363|ABCA4_HUMAN  MPSAGMLPWLQGIFCNVNNPCFQSPTPGESPGIVSNYNNSILARVYRDFQELLMNAPESQ 120
SP|Q8IZY2|ABCA7_HUMAN  LPSAGTVPWLQGLICNVNNTCFPQLTPGEEPGRLSNFNDSLVSRLLADARTVLGGASAHR 120
                       :**** :**:**::**.** **    **** ** :.*:*.*:::*:   * : :*   :

                                                   ECD1
SP|O95477|ABCA1_HUMAN  SMKDMRKVLRTLQQI---------KKSSSNLKLQDFLVDNETFSGFLYHNLSLPKSTVDK 171
SP|P78363|ABCA4_HUMAN  HLGRIWTELHILSQFMDTLRTHPERIAGRGIRIRDILKDEETLTLFLIKNIGLSDSVVYL 180
SP|Q8IZY2|ABCA7_HUMAN  TLAGLGKLIATLRAARSTAQPQPTKQSPLE------------------------------ 150
                        :  : . :  *               : :

                                                   ECD1
SP|O95477|ABCA1_HUMAN  MLRADVILHKVFLQGY-QLHLT-SLCNGSKSEEMIQLGD-----QEVSELCGLPREKLAA 224
SP|P78363|ABCA4_HUMAN  LINSQVRPE-QFAHGVPDLALKDIACSEALLERFIIFSQRRGAKTVRYALCSLSQGTLQW 239
SP|Q8IZY2|ABCA7_HUMAN  ---------------PPMLDVAELLTSLL-----------------RTESLGLALG---- 174
                                         * :      .                        .*

                                                   ECD1
SP|O95477|ABCA1_HUMAN  AERVLRSNMDILKPILRTLNSTSPFPSKELAEATKTLLHSLGTLAQELFSMRSWSDMRQ- 283
SP|P78363|ABCA4_HUMAN  IEDTLYANVDFFKLFR-VLP---------------TLL----DSRSQGINLRSWGGILSD 279
SP|Q8IZY2|ABCA7_HUMAN  ----------------------------QAQEPLHSLLEAAEDLAQELLALRSLVELRAL 206
                                                          :**       .: : :**    :

                                                   ECD1
SP|O95477|ABCA1_HUMAN  ---------------EVMFLT----NVNSSSSSTQIYQAVSRIVCGHPEGGGLKIKSLNW 324
SP|P78363|ABCA4_HUMAN  MSPRIQEFIHRPSMQDLLWVTRPLMQNGGPETFTKLMGILSDLLCGYPEGGGSRVLSFNW 339
SP|Q8IZY2|ABCA7_HUMAN  --------------------------LQRPRGTSGPLELLSEALCSVRGPSSTVGPSLNW 240
                                                         :     :*   :*.     ..    *:**

                                                   ECD1
SP|O95477|ABCA1_HUMAN  YEDNNYKALFGGNGTEEDAETFYDNSTTPYCNDLMKNLESSPLSRIIWKALKPLLVGKIL 384
SP|P78363|ABCA4_HUMAN  YEDNNYKAFLGIDSTRKDPIYSYDRRTTSFCNALIQSLESNPLTKIAWRAAKPLLMGKIL 399
SP|Q8IZY2|ABCA7_HUMAN  YEASDLMELVGQEPES----ALPDSSLSPACSELIGALDSHPLSRLLWRRLKPLILGKLL 296
                       **  .:    :.* :          *   :  *. *:  *:* **::: *:   ***::**:*

                                                   ECD1
SP|O95477|ABCA1_HUMAN  YTPDTPATRQVMAEVNKTFQELAVFHDLEGMWEELSPKIWTFMENSQEMDLVRMLLDSRD 444
SP|P78363|ABCA4_HUMAN  YTPDSPAARRILKNANSTFEELEHVRKLVKAWEEVGPQIWYFFDNSTQMNMIRDTLGNPT 459
SP|Q8IZY2|ABCA7_HUMAN  FAPDTPFTRKLMAQVNRTFEELTLLRDVREVWEMLGPRIFTFMNDSSNVAMLQRLLQMQD 356
                       ::**:* :*::: :.* **:**  .:.:    ** :.*:*: *:::* :: :::   *

                                                   ECD1
SP|O95477|ABCA1_HUMAN  NDHFWEQQLDGLDWTAQDIVAFLAKHPEDVQSSNGSVYTWREAFNETNQAIRTISRFMEC 504
SP|P78363|ABCA4_HUMAN  VKDFLNRQLGEEGITAEAILNFLYKGPRESQADDMANFDWRDIFNITDRTLRLVNQYLEC 519
SP|Q8IZY2|ABCA7_HUMAN  EGRRQPRPGGRD--HMEALRSFL--------DPGSGGYSWQDAHADVGHLVGTLGRVTEC 406
                             :  .     : :  **           .  . : *::  .   ..: :   :.:   **

                                                   ECD1
SP|O95477|ABCA1_HUMAN  VNLNKLEPIATEVWLINKSMELLDERKFWAGIVFTGITP--------GSIELPHHVKYKI 556
SP|P78363|ABCA4_HUMAN  LVLDKFESYNDETQLTQRALSLLEENMFWAGVVFPDMYP--------WTSSLPPHVKYKI 571
SP|Q8IZY2|ABCA7_HUMAN  LSLDKLEAAPSEAALVSRALQLLAEHRFWAGVVFLGPEDSSDPTEHPTPDLGPGHVRIKI 466
                       : *:*:*    *. *  .:::.** *.  ****:** .                 * **: **

                                                   ECD1
SP|O95477|ABCA1_HUMAN  RMDIDNVERTNKIKDGYWDPGPRADPFEDMRYVWGGFAYLQDVVEQAIIRVLTGTEKKTG 616
SP|P78363|ABCA4_HUMAN  RMDIDVVEKTNKIKDRYWDSGPRADPVEDFRYIWGGFAYLQDMVEQGITRSQVQAEAPVG 631
SP|Q8IZY2|ABCA7_HUMAN  RMDIDVVTRTNKIRDRFWDPGPAADPLTDLRYVWGGFVYLQDLVERAAVRVLSGANPRAG 526
                       ***** * :****:* :** ** ***. *:**:****.****:**:.  *    ::  .*
```

```
                                   ECD1                            TM2                    IH2
SP|O95477|ABCA1_HUMAN VYMQQMPYPCYVDDIFLRVMSRSMPLFMTLAWIYSVAVIIKGIVYEKEARLKETMRIMGL 676
SP|P78363|ABCA4_HUMAN IYLQQMPYPCFVDDSFMIILNRCFPIFMVLAWIYSVSMTVKSIVLEKELRLKETLKNQGV 691
SP|Q8IZY2|ABCA7_HUMAN LYLQQMPYPCYVDDVFLRVLSRSLPLFLTLAWIYSVTLTVKAVVREKETRLRDTMRAMGL 586
                      :*:*******:*** *: ::.*.:*:*:.*******:: :*.:* *** **::*::  *:

                                    TM3                               TM4
SP|O95477|ABCA1_HUMAN DNSILWFSWFISSLIPLLVSAGLLVVILKLGNLLPYSDPSVVFVFLSVFAVVTILQCFLI 736
SP|P78363|ABCA4_HUMAN SNAVIWCTWFLDSFSIMSMSIFLLTIFIMHGRILHYSDPFILFLFLLAFSTATIMLCFLL 751
SP|Q8IZY2|ABCA7_HUMAN SRAVLWLGWFLSCLGPFLLSAALLVLVLKLGDILPYSHPGVVFLFLAAFAVATVTQSFLL 646
                      ..:::*  **:..:  : :*  **.:.:  * :* **.* ::*:** .*:..*:  .**:

                      TM4           TM5                   EH1          EH2
SP|O95477|ABCA1_HUMAN STLFSRANLAAACGGIIYFTLYLPYVLCVAWQDYVGFTLKIFASLLSPVAFGFGCEYFAL 796
SP|P78363|ABCA4_HUMAN STFFSKASLAAACSGVIYFTLYLPHILCFAWQDRMTAELKKAVSLLSPVAFGFGTEYLVR 811
SP|Q8IZY2|ABCA7_HUMAN SAFFSRANLAAACGGLAYFSLYLPYVLCVAWRDRLPAGGRVAASLLSPVAFGFGCESLAL 706
                      *::**:*.*****.*: **:****::**.**:* :     :  .*********** * :.

                      EH2                             TM6
SP|O95477|ABCA1_HUMAN FEEQGIGVQWDNLFESPVEEDGFNLTTSVSMMLFDTFLYGVMTWYIEAVFPGQYGIPRPW 856
SP|P78363|ABCA4_HUMAN FEEQGLGLQWSNIGNSPTEGDEFSFLLSMQMMLLDAAVYGLLAWYLDQVFPGDYGTPLPW 871
SP|Q8IZY2|ABCA7_HUMAN LEEQGEGAQWHNVGTRPTA-DVFSLAQVSGLLLLDAALYGLATWYLEAVCPGQYGIPEPW 765
                      :**** * ** *:    *.   * *.:     ::*:*: :**: :**:: * **:** * **

                                                                          NBD1
SP|O95477|ABCA1_HUMAN YFPCTKSYWFGEESDEKSHPGS---------------NQKRISEICMEEEPTHLKLGVSI 901
SP|P78363|ABCA4_HUMAN YFLLQESYWLGGEGCSTREERALEKTEPLTEETEDPEHPEGIHDSFFEREHPGWVPGVCV 931
SP|Q8IZY2|ABCA7_HUMAN NFPFRRSYWCGPRPPKSPAPCP----------------TPLDPKVLVEEAPPGLSPGVSV 809
                       *   .*** *  .  ..                        .  .*.        **.:

                                   NBD1              Walker A motif
SP|O95477|ABCA1_HUMAN QNLVKVYRDGMKVAVDGLALNFYEGQITSFLGHNGAGKTTTMSILTGLFPPTSGTAYILG 961
SP|P78363|ABCA4_HUMAN KNLVKIFEPCGRPAVDRLNITFYENQITAFLGHNGAGKTTTLSILTGLLPPTSGTVLVGG 991
SP|Q8IZY2|ABCA7_HUMAN RSLEKRFPGSPQPALRGLSLDFYQGHITAFLGHNGAGKTTTLSILSGLFPPSGGSAFILG 869
                      :.* * :     : *:   * : **:.:**:***********:***:**:**:.*:. : *

                                                 NBD1
SP|O95477|ABCA1_HUMAN KDIRSEMSTIRQNLGVCPQHNVLFDMLTVEEHIWFYARLKGLSEKHVKAEMEQMALDVGL 1021
SP|P78363|ABCA4_HUMAN RDIETSLDAVRQSLGMCPQHNILFHHLTVAEHMLFYAQLKGKSQEEAQLEMEAMLEDTGL 1051
SP|Q8IZY2|ABCA7_HUMAN HDVRSSMAAIRPHLGVCPQYNVLFDMLTVDEHVWFYGRLKGLSAAVVGPEQDRLLQDVGL 929
                      :*:.:.: ::*  **:***:*:**. *** **: **.:*** *    .  *  :  :  *.**

                             ABC signature motif       Walker B motif       NBD1
SP|O95477|ABCA1_HUMAN PSSKLKSKTSQLSGGMQRKLSVALAFVGGSKVVILDEPTAGVDPYSRRGIWELLLKYRQG 1081
SP|P78363|ABCA4_HUMAN HHKR-NEEAQDLSGGMQRKLSVAIAFVGDAKVVILDEPTSGVDPYSRRSIWDLLLKYRSG 1110
SP|Q8IZY2|ABCA7_HUMAN VSKQ-SVQTRHLSGGMQRKLSVAIAFVGGSQVVILDEPTAGVDPASRRGIWELLLKYREG 988
                        .:  .  ::  .************:****.::*********:**** ***.**:******.*

                                                 NBD1                   NBD1 / R1
SP|O95477|ABCA1_HUMAN RTIILSTHHMDEADVLGDRIAIISHGKLCCVGSSLFLKNQLGTGYYLTLVKKDVESSL-- 1139
SP|P78363|ABCA4_HUMAN RTIIMSTHHMDEADLLGDRIAIIAQGRLYCSGTPLFLKNCFGTGLYLTLVRKMKNIQSQR 1170
SP|Q8IZY2|ABCA7_HUMAN RTLILSTHHLDEAELLGDRVAVVAGGRLCCCGSPLFLRRHLGSGYYLTLVKARLPLTTNE 1048
                      **:*:****:***::****:*::: *:* * *: ***:. :*:* *****:

                         NBD1 / R1                                         R1
SP|O95477|ABCA1_HUMAN ----SSCRNSSSTVSYLKKEDSVSQSSSDAGLGSDHESDTLTIDVSAISNLIRKHVSEAR 1195
SP|P78363|ABCA4_HUMAN KGSEGTCSCSSKGFST----------TCPAHVDDLTPEQVLDGDVNELMDVVLHHVPEAK 1220
SP|Q8IZY2|ABCA7_HUMAN KADTDME------------------GSVDTRQEKKNGSQGSRVGTPQLLALVQHWVPGAR 1090
                          .                    :  :   .   .:    ..  :  :: : *  *:

                                                 R1
SP|O95477|ABCA1_HUMAN LVEDIGHELTYVLPYEAAKEGAFVELFHEIDDRLSDLGISSYGISETTLEEIFLKVAEES 1255
SP|P78363|ABCA4_HUMAN LVECIGQELIFLLPNKNFKHRAYASLFRELEETLADLGLSSFGISDTPLEEIFLKVTEDS 1280
SP|Q8IZY2|ABCA7_HUMAN LVEELPHELVLVLPYTGAHDGSFATLFRELDTRLAELRLTGYGISDTSLEEIFLKVVEEC 1150
                      *** : :**  :**     :. ::. **:*::  *::* ::.:***:* ********.*:.
```

```
SP|O95477|ABCA1_HUMAN GVDAETSDGTLPARRNRRAFGDKQSCLRPFTEDD----AADPNDSDI--------DPESR 1303
SP|P78363|ABCA4_HUMAN DSGPLFAGGAQQ---KRENVNPRHPCLGPREKAG----Q-TPQDSNVCSPGAPAAHPEGQ 1332
SP|Q8IZY2|ABCA7_HUMAN AADTDMEDGSCGQ----------HLCTGIAGLDVTLRLKMPPQETAL-ENGEPAGS--AP 1197
                        .    .*:             : *               *::: :           .

                                                   IH3                    TM7
SP|O95477|ABCA1_HUMAN ETDLLSGMDGKGSYQVKGWKLTQQQFVALLWKRLLIARRSRKGFFAQIVLPAVFVCIALV 1363
SP|P78363|ABCA4_HUMAN P---PPEPECPGPQLNTGTQLVLQHVQALLVKRFQHTIRSHKDFLAQIVLPATFVFLALM 1389
SP|Q8IZY2|ABCA7_HUMAN ETDQGSGPDAV--GRVQGWALTRQQLQALLLKRFLLARRSRRGLFAQIVLPALFVGLALV 1255
                              :        *  *. *:. *** **:  : **::.::******* ** :**:

                     TM7                          ECD2
SP|O95477|ABCA1_HUMAN FSLIVPPFGKYPSLELQPWMYNEQYTFVSNDAPEDTGTLELLNALTKDPGFGTRCMEGNP 1423
SP|P78363|ABCA4_HUMAN LSIVIPPFGEYPALTLHPWIYGQQYTFFSMDEPGSEQFTVLADVLLNKPGFGNRCLKEGW 1449
SP|Q8IZY2|ABCA7_HUMAN FSLIVPPFGHYPALRLSPTMYGAQVSFFSEDAPGDPGRARLLEALLQEAGL--------- 1306
                      :*:::****.**:* * * :*. * :*.* * * .     * :.* :. *:

                                                  ECD2
SP|O95477|ABCA1_HUMAN IPDTPCQAGEEEWTTAPVPQTIMDLFQNGNWTMQNPSPACQCSSDKIKKMLPVCPPGAGG 1483
SP|P78363|ABCA4_HUMAN LPEYPCGN-STPWKTPSVSPNITQLFQKQKWTQVNPSPSCRCSTREKLTMLPECPEGAGG 1508
SP|Q8IZY2|ABCA7_HUMAN -EEPPVQHSSHRFSAPEVPAEVAKVLASGNWTPESPSPACQCSRPGARRLLPDCPAAAGG 1365
                        : *    .  :.:  *   : .:: . :**  .***:*:**      :** ** .***

                                                  ECD2
SP|O95477|ABCA1_HUMAN LPPPQRKQNTADILQDLTGRNISDYLVKTYVQIIAKSLKNKIWVNEFRYGGFSLGVSNTQ 1543
SP|P78363|ABCA4_HUMAN LPPPQRTQRSTEILQDLTDRNISDFLVKTYPALIRSSLKSKFWVNEQRYGGISIGGKLPV 1568
SP|Q8IZY2|ABCA7_HUMAN PPPPQAVTGSGEVVQNLTGRNLSDFLVKTYPRLVRQGLKTKKWVNEVRYGGFSLGGRDPG 1425
                       ****    : :::*:**.**:**:*****  :: ..**.* **** ****:*:*

                                                  ECD2
SP|O95477|ABCA1_HUMAN ALPPSQEVNDAIKQMKKHLKLAKDSSADRFLNSLGRFMTGLDTKNNVKVWFNNKGWHAIS 1603
SP|P78363|ABCA4_HUMAN VPITGEALVGFLSDLGRIMNVSGGPITREASKEIPDFLKHLETEDNIKVWFNNKGWHALV 1628
SP|Q8IZY2|ABCA7_HUMAN L-PSGQELGRSVEELWALLSPLPGGALDRVLKNLTAWAHSLDAQDSLKIWFNNKGWHSMV 1484
                          .: :   :.::   :.   .    .  :.:  :   *::::.:*:*********::

                                ECD2                       TM8
SP|O95477|ABCA1_HUMAN SFLNVINNAILRANLQKGENPSHYGITAFNHPLNLTKQQLSEVALMTTSVDVLVSICVIF 1663
SP|P78363|ABCA4_HUMAN SFLNVAHNAILRASLPKDRSPEEYGITVISQPLNLTKEQLSEITVLTTSVDAVVAICVIF 1688
SP|Q8IZY2|ABCA7_HUMAN AFVNRASNAILRAHLPPGPARHAHSITTLNHPLNLTKEQLSEGALMASSVDVLVSICVVF 1544
                      :*:*   ****** *  .     :.**.:.:******:**** :::::***.:*:***:*

                             TM8             IH4                   TM9
SP|O95477|ABCA1_HUMAN AMSFVPASFVVFLIQERVSKAKHLQFISGVKPVIYWLSNFVWDMCNYVVPATLVIIIFIC 1723
SP|P78363|ABCA4_HUMAN SMSFVPASFVLYLIQERVNKSKHLQFISGVSPTTYWVTNFLWDIMNYSVSAGLVVGIFIG 1748
SP|Q8IZY2|ABCA7_HUMAN AMSFVPASFTLVLIEERVTRAKHLQLMGGLSPTLYWLGNFLWDMCNYLVPACIVVLIFLA 1604
                      :********.: **:***.::****::.*:.*. **: **:**: ** * * :*: **:

                      TM9                 TM10                          TM11
SP|O95477|ABCA1_HUMAN FQQKSYVSSTNLPVLALLLLLYGWSITPLMYPASFVFKIPSTAYVVLTSVNLFIGINGSV 1783
SP|P78363|ABCA4_HUMAN FQKKAYTSPENLPALVALLLLYGWAVIPMMYPASFLFDVPSTAYVALSCANLFIGINSSA 1808
SP|Q8IZY2|ABCA7_HUMAN FQQRAYVAPANLPALLLLLLLYGWSITPLMYPASFFFSVPSTAYVVLTCINLFIGINGSM 1664
                      **:::*.:  ***.*  *******:: *:******.*.:******.*:. *******.*

                       TM11              EH3                  EH4
SP|O95477|ABCA1_HUMAN ATFVLELFTDNK-LNNINDILKSVFLIFPHFCLGRGLIDMVKNQAMADALERFGENRFVS 1842
SP|P78363|ABCA4_HUMAN ITFILELFENNRTLLRFNAVLRKLLIVFPHFCLGRGLIDLALSQAVTDVYARFGEEHSAN 1868
SP|Q8IZY2|ABCA7_HUMAN ATFVLELFSDQK-LQEVSRILKQVFLIFPHFCLGRGLIDMVRNQAMADAFERLGDRQFQS 1723
                       **:**** ::: * ... :*:.::::************:. .**::*.  *:*:.:  .

                                         TM12                             NBD2
SP|O95477|ABCA1_HUMAN PLSWDLVGRNLFAMAVEGVVFFLITVLIQYRFFIRPRPVNAKLSPLNDEDEDVRRERQRI 1902
SP|P78363|ABCA4_HUMAN PFHWDLIGKNLFAMVVEGVVYFLLTLLVQRHFFLSQWIAEPTKEPIVDEDDDVAEERQRI 1928
SP|Q8IZY2|ABCA7_HUMAN PLRWEVVGKNLLAMVIQGPLFLLFTLLLQHRSQLLPQPRVRSLPLLGEEDEDVARERERV 1783
                      *: *:::*:**:**.::* :::*:*:*:* :  :       .   : :**:** .**:*:
```

```
                                            NBD2                 Walker A motif
SP|O95477|ABCA1_HUMAN LDGGGQNDILEIKELTKIYRRKRKPAVDRICVGIPPGECFGLLGVNGAGKSSTFKMLTGD 1962
SP|P78363|ABCA4_HUMAN ITGGNKTDILRLHELTKIYPGTSSPAVDRLCVGVRPGECFGLLGVNGAGKTTTFKMLTGD 1988
SP|Q8IZY2|ABCA7_HUMAN VQGATQGDVLVLRNLTKVYRGQRMPAVDRLCLGIPPGECFGLLGVNGAGKTSTFRMVTGD 1843
                      : *. : *:* :::***:*     *****:*:*: ***************::**:*:***
                                                  NBD2
SP|O95477|ABCA1_HUMAN TTVTRGDAFLNKNSILSNIHEVHQNMGYCPQFDAITELLTGREHVEFFALLRGVPEKEVG 2022
SP|P78363|ABCA4_HUMAN TTVTSGDATVAGKSILTNISEVHQNMGYCPQFDAIDELLTGREHLYLYARLRGVPAEEIE 2048
SP|Q8IZY2|ABCA7_HUMAN TLASRGEAVLAGHSVAREPSAAHLSMGYCPQSDAIFELLTGREHLELLARLRGVPEAQVA 1903
                      * .: *:* :  :*:  :   .* .****** *** ********: : * *****  ::
                              NBD2     ABC signature motif        Walker B motif
SP|O95477|ABCA1_HUMAN KVGEWAIRKLGLVKYGEKYAGNYSGGNKRKLSTAMALIGGPPVVFLDEPTTGMDPKARRF 2082
SP|P78363|ABCA4_HUMAN KVANWSIKSLGLTVYADCLAGTYSGGNKRKLSTAIALIGCPPLVLLDEPTTGMDPQARRM 2108
SP|Q8IZY2|ABCA7_HUMAN QTAGSGLARLGLSWYADRPAGTYSGGNKRKLATALALVGDPAVVFLDEPTTGMDPSARRF 1963
                      :..  .:  ***  *.:  **.*********:**:**:* * :*:**********.***:
                                                  NBD2                   NBD2 / R2
SP|O95477|ABCA1_HUMAN LWNCALSVVKEGRSVVLTSHSMEECEALCTRMAIMVNGRFRCLGSVQHLKNRFGDGYTIV 2142
SP|P78363|ABCA4_HUMAN LWNVIVSIIREGRAVVLTSHSMEECEALCTRLAIMVKGAFRCMGTIQHLKSKFGDGYIVT 2168
SP|Q8IZY2|ABCA7_HUMAN LWNSLLAVVREGRSVMLTSHSMEECEALCSRLAIMVNGRFRCLGSPQHLKGRFAAGHTLT 2023
                      ***  :::::***:*:*************:*:****:* ***:*: ****.:*. *: :.
                  NBD2 / R2                                  R2
SP|O95477|ABCA1_HUMAN VRIAGS----NPDLKPVQDFFGLAFPGSVLKEKHRNMLQYQLPSS-LSSLARIFSILSQS 2197
SP|P78363|ABCA4_HUMAN MKIKSPKDDLLPDLNPVEQFFQGNFPGSVQRERHYNMLQFQVSS---SSLARIFQLLLSH 2225
SP|Q8IZY2|ABCA7_HUMAN LRVPAAR------SQPAAAFVAAEFPGAELREAHGGRLRFQLPPGGRCALARVFGELAVH 2077
                      ::: .         :*.  *.   ***:  :* * . *::*:     .:***:*  *
                                    R2
SP|O95477|ABCA1_HUMAN KKRLHIEDYSVSQTTLDQVFVNFAKDQSDDDHLKDLSL-----HKNQTVVDVAVLTSFLQ 2252
SP|P78363|ABCA4_HUMAN KDSLLIEEYSVTQTTLDQVFVNFAKQQTESHDLP---L-----HPRAAGASRQ-----AQ 2272
SP|Q8IZY2|ABCA7_HUMAN GAEHGVEDFSVSQTMLEEVFLYFSKDQGKDEDTEEQKEAGVGVDPAPGLQHPKRVSQFLD 2137
                           :*::**:** *::**: *:*:* ....           .               :

SP|O95477|ABCA1_HUMAN DEKVKESYV 2261
SP|P78363|ABCA4_HUMAN D-------- 2273
SP|Q8IZY2|ABCA7_HUMAN DPSTAETVL 2146
```

Supplementary Figure S1. Sequential information and sequence alignment of the human ABC transporters ABCA1 (UniProt ID: O95477), ABCA4 (UniProt ID: P78363), and ABCA7 (UniProt ID: Q8IZY2); * = identity; : = high similarity; .= moderate similarity; - = gap; the structural features of ABCA1, ABCA4, and ABCA7 have been highlighted in **green**, **blue**, and **red**, respectively. Since most HIs as well as the EHs and ECDs could not clearly be defined in the cryo-EM structures of ABCA1 [96] and ABCA4 [98, 163], these regions have been marked in light grey, dark grey, and black for ABCA1, ABCA4, and ABCA7, respectively. Amino acids for the binding of pan-ABC transporter inhibitors that are proposed to constitute the multitarget binding site in ABCA7 are highlighted in **blue/yellow**.

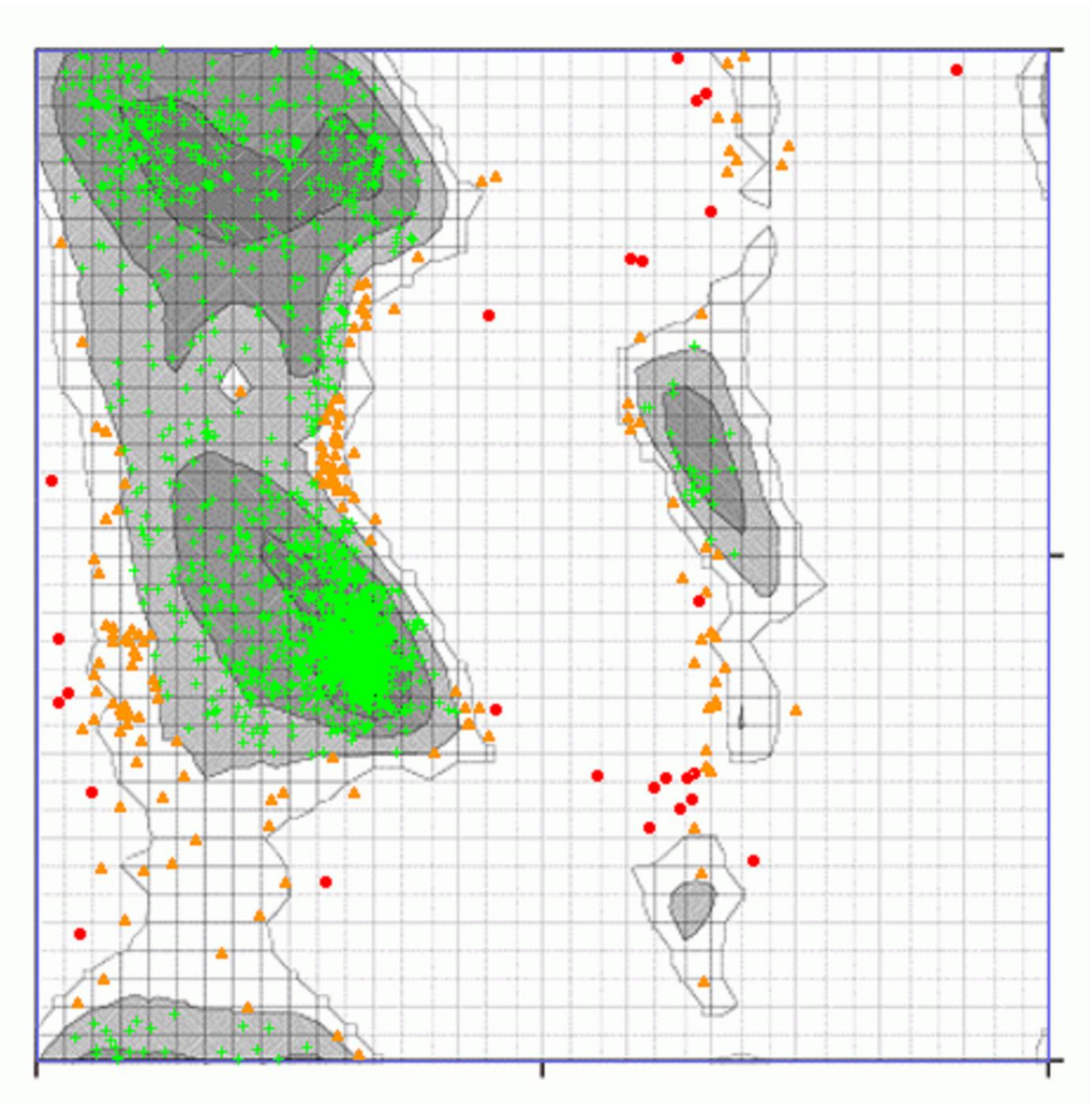

Supplementary Figure S2: Ramachandran plot of the human ABCA7 model: 89.9% of all residues map to the most favored regions (green crosses), 8.7% in the allowed regions (yellow triangles), and 1.4% in the critical regions (red dots).

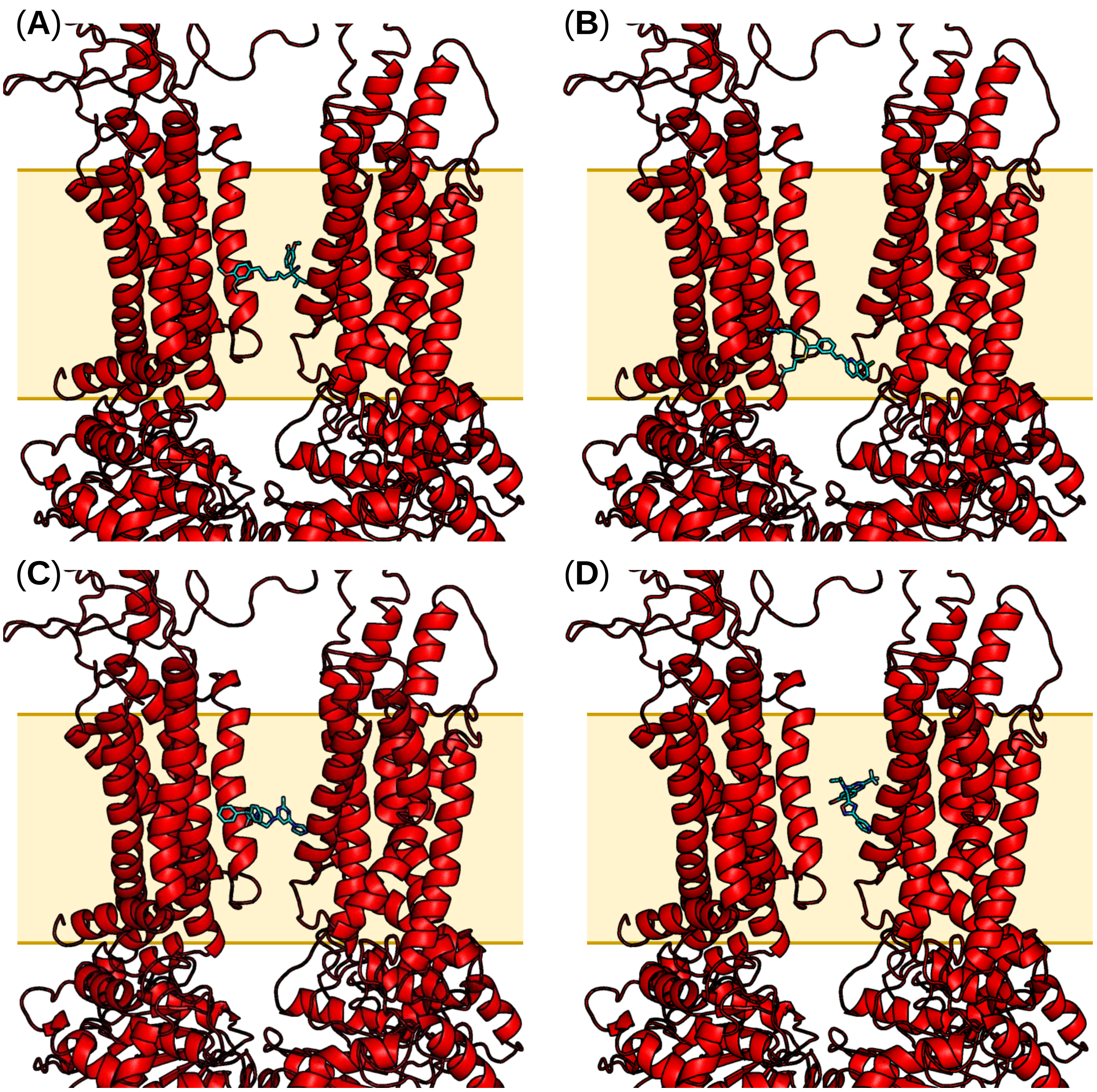
(A)
(B)
(C)
(D)

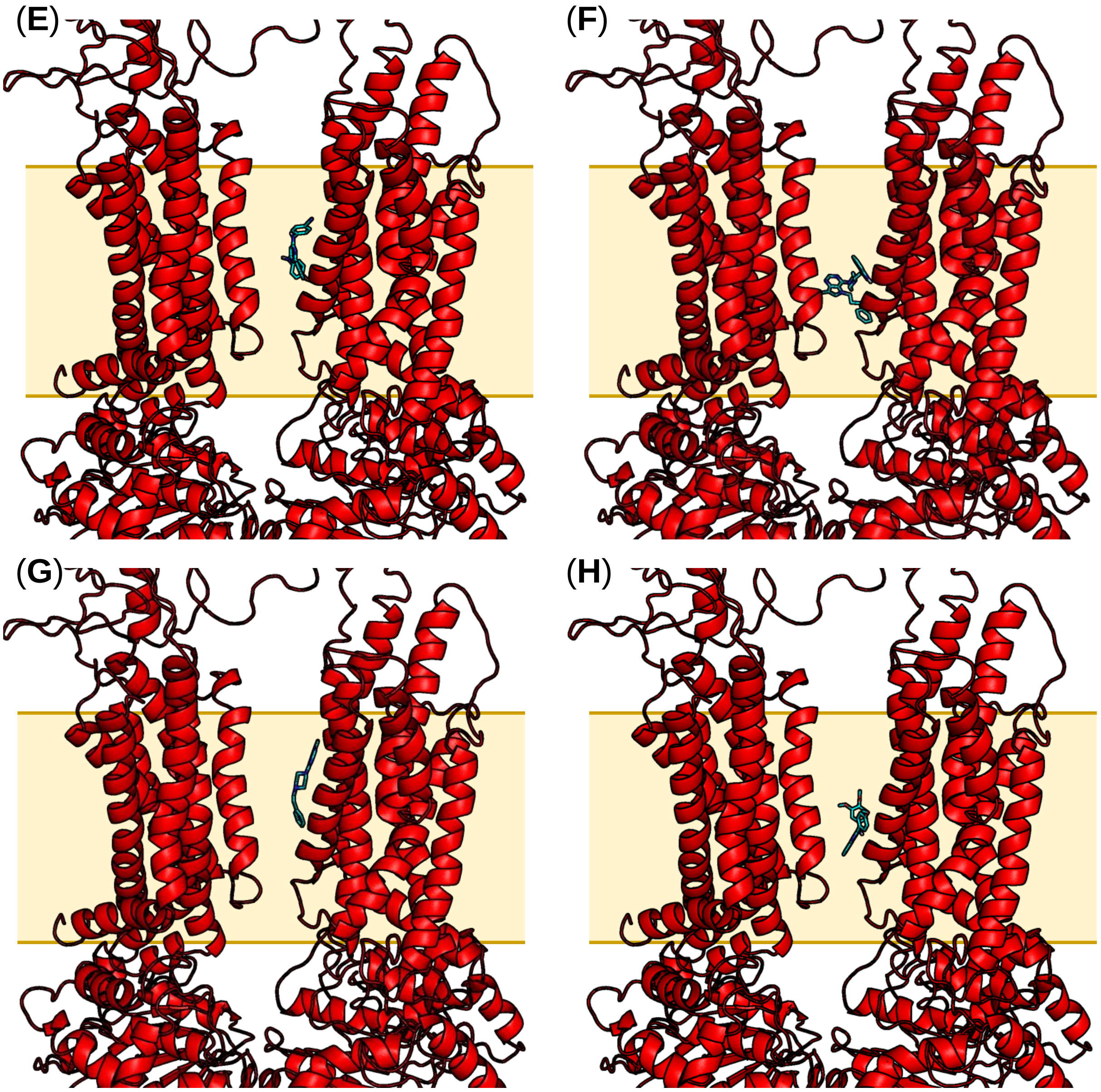
(E)
(F)
(G)
(H)

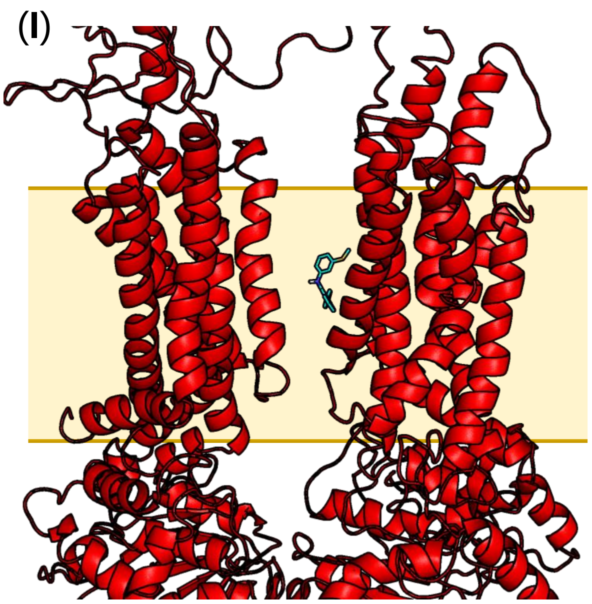

Supplementary Figure S3. The top ranking poses of the 10 docked pan-ABC transporter inhibitors **9** (**A**), **10** (**B**), **11** (**C**), **14** (**D**), **17** (**E**), **22** (**F**), **23** (**G**), **24** (**H**), **25** (**I**), and **26** (**J**) [38, 45, 101, 134-137] (colored cyan, stick representation) in the homology model of ABCA7 (colored red, cartoon representation; the inter-membrane space is indicated as light brown area, and the border to the cytosol and lumen is indicated by brown lines) using AutoDock [129]. Nonpolar hydrogen atoms were omitted, and polar hydrogen, carbon, nitrogen, oxygen, as well as sulfur atoms were colored in silver, cyan, blue, red, and dark yellow, respectively.

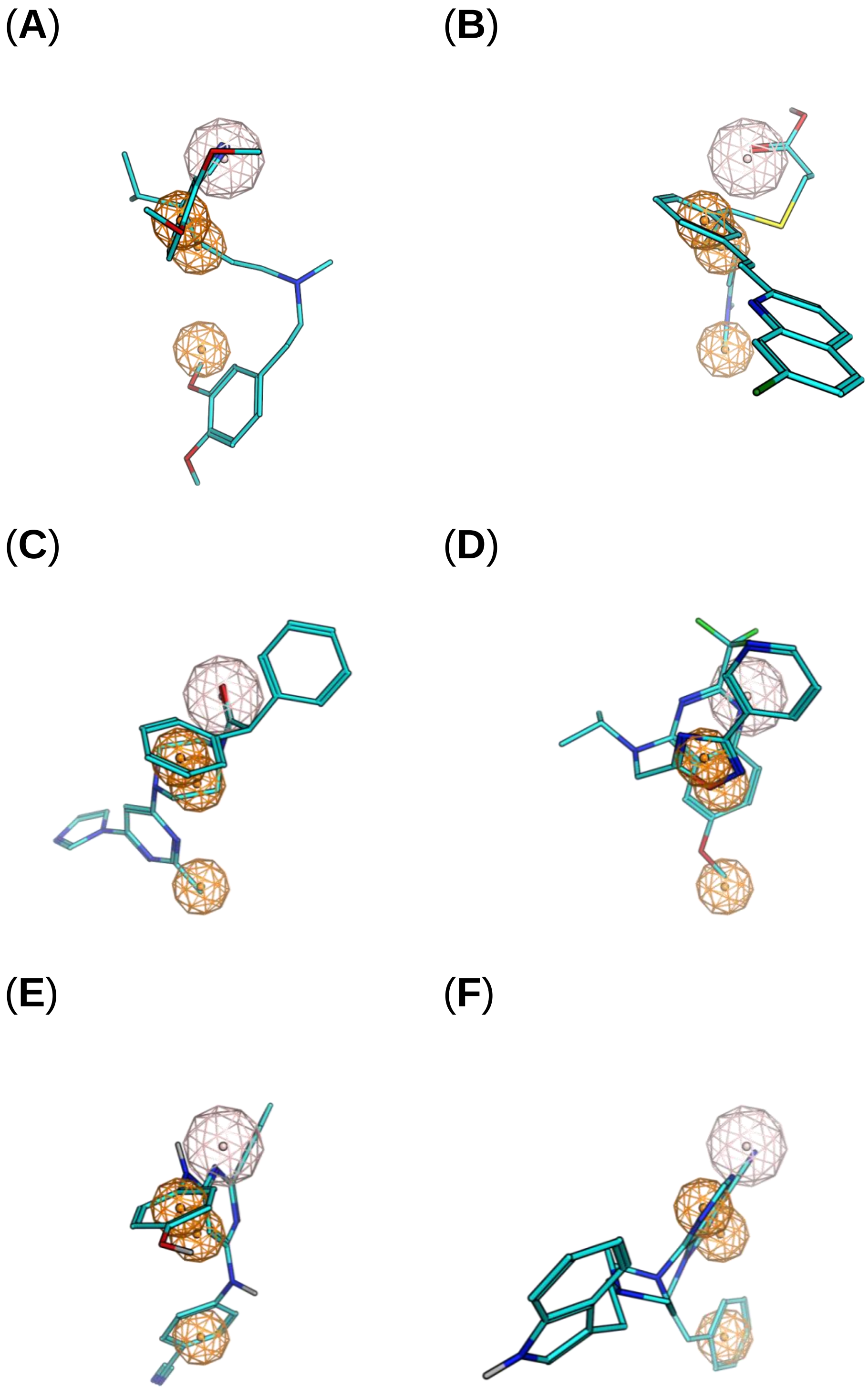
(A)
(B)
(C)
(D)
(E)
(F)

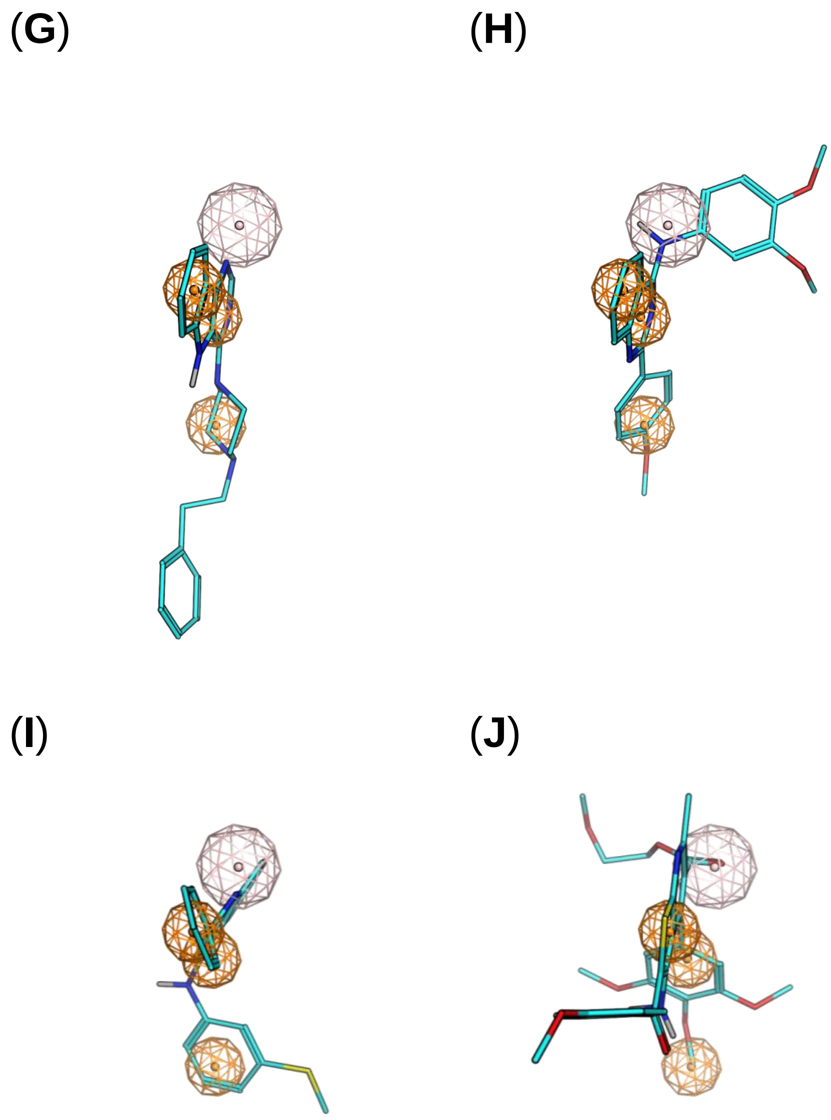

Supplementary Figure S4. The binding poses of the 10 selected pan-ABC transporter inhibitors **9** (**A**), **10** (**B**), **11** (**C**), **14** (**D**), **17** (**E**), **22** (**F**), **23** (**G**), **24** (**H**), **25** (**I**), and **26** (**J**) [38, 45, 101, 134-137] (colored cyan, stick representation) obtained from AutoDock [129] screened against the developed pharmacophore model generated from the top ranking docking poses as obtained from Autodock [129]. The best fitting individual molecule is shown superimposed with the four pharmacophore features F1–F2 (aromatic/hydrophobic; orange), F3 (aromatic, orange), and F4 (acceptor/donor; silver)**.** Nonpolar hydrogen atoms were omitted, and polar hydrogen, carbon, nitrogen, oxygen, as well as sulfur atoms were colored in silver, cyan, blue, red, and dark yellow, respectively.

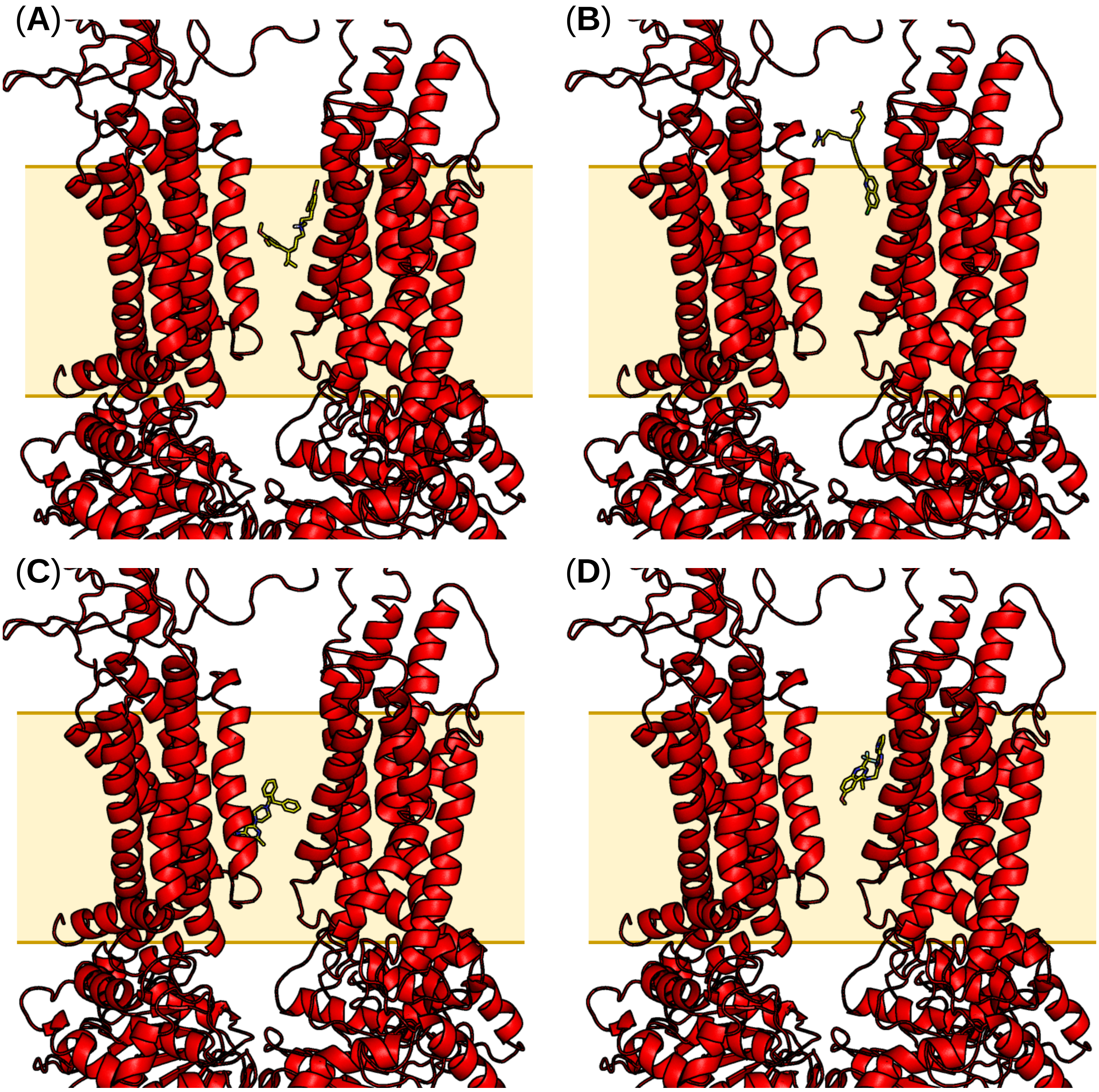
(A)
(B)
(C)
(D)

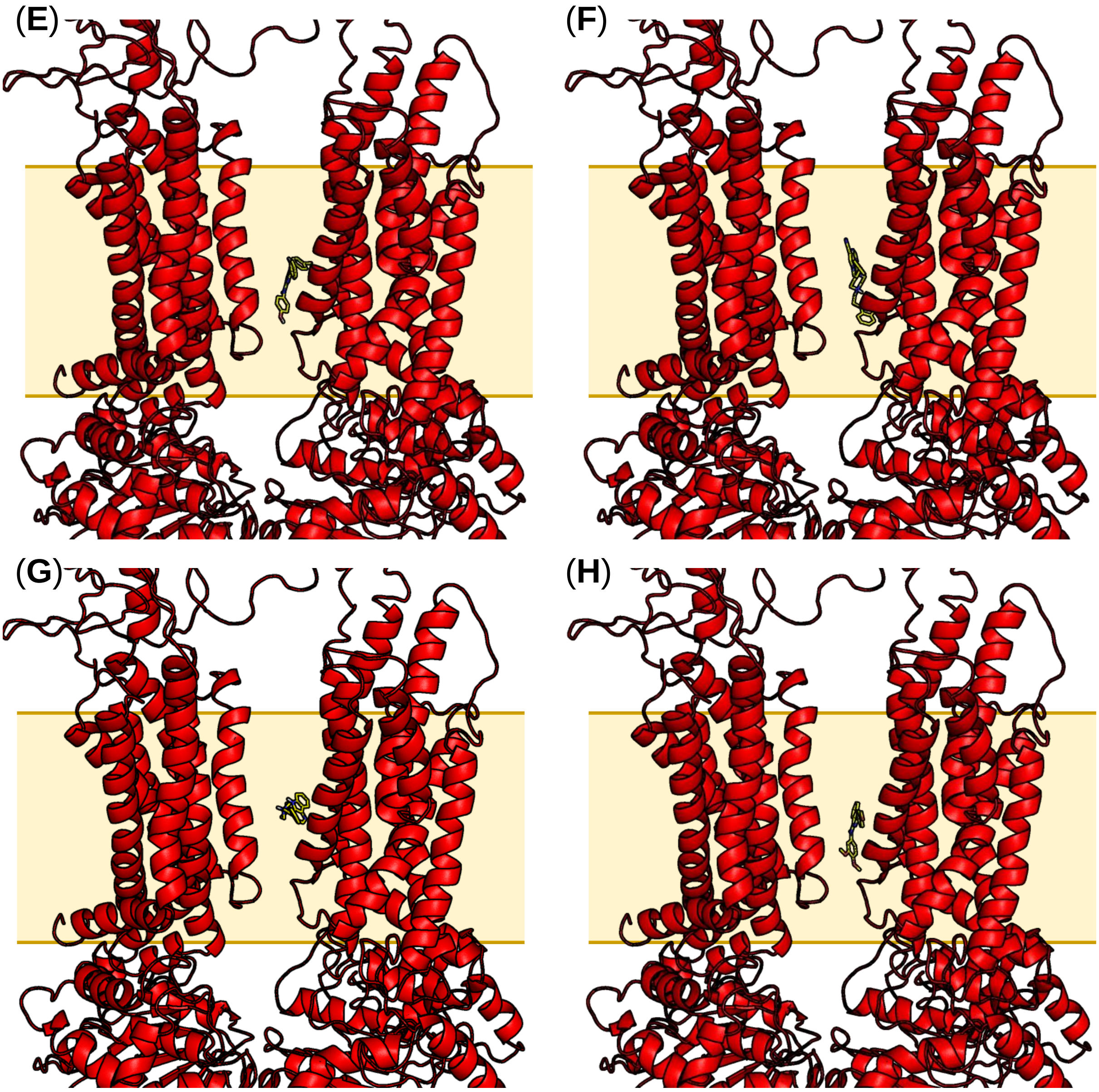
(E)
(F)
(G)
(H)

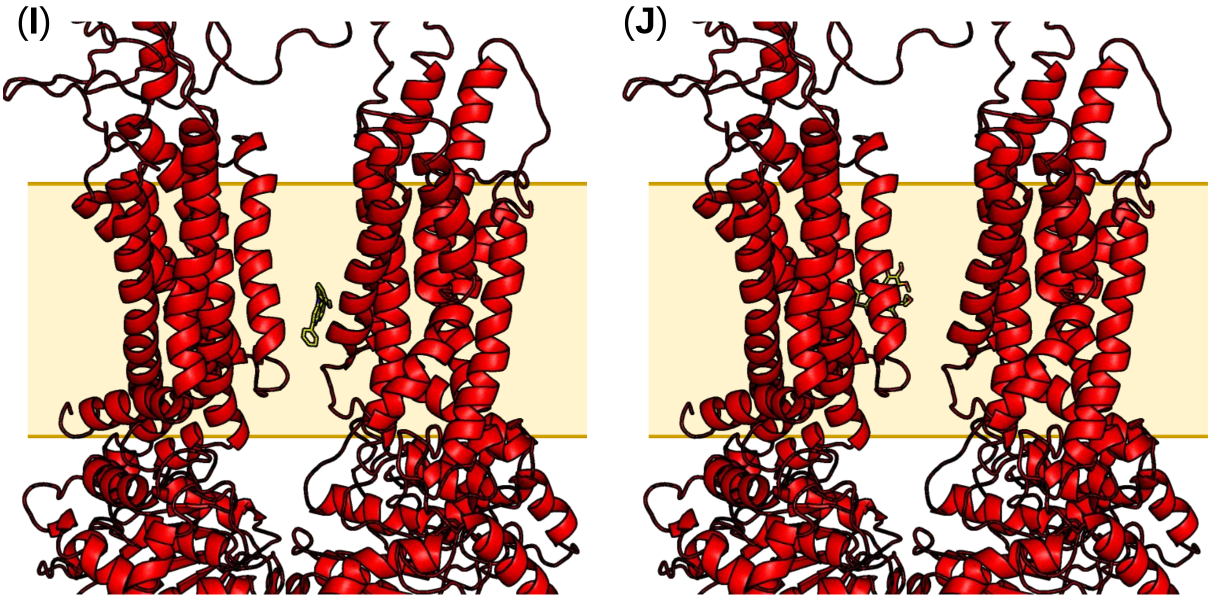

Supplementary Figure S5. The top ranking poses of the 10 docked pan-ABC transporter inhibitors **9** (**A**), **10** (**B**), **11** (**C**), **14** (**D**), **17** (**E**), **22** (**F**), **23** (**G**), **24** (**H**), **25** (**I**), and **26** (**J**) [38, 45, 101, 134-137] (colored yellow, stick representation) in the homology model of ABCA7 (colored red, cartoon representation; the inter-membrane space is indicated as light brown area, and the border to the cytosol and lumen is indicated by brown lines) using Glide [141, 142]. Nonpolar hydrogen atoms were omitted, and polar hydrogen, carbon, nitrogen, oxygen, as well as sulfur atoms were colored in silver, dark yellow, blue, red, and yellow, respectively.

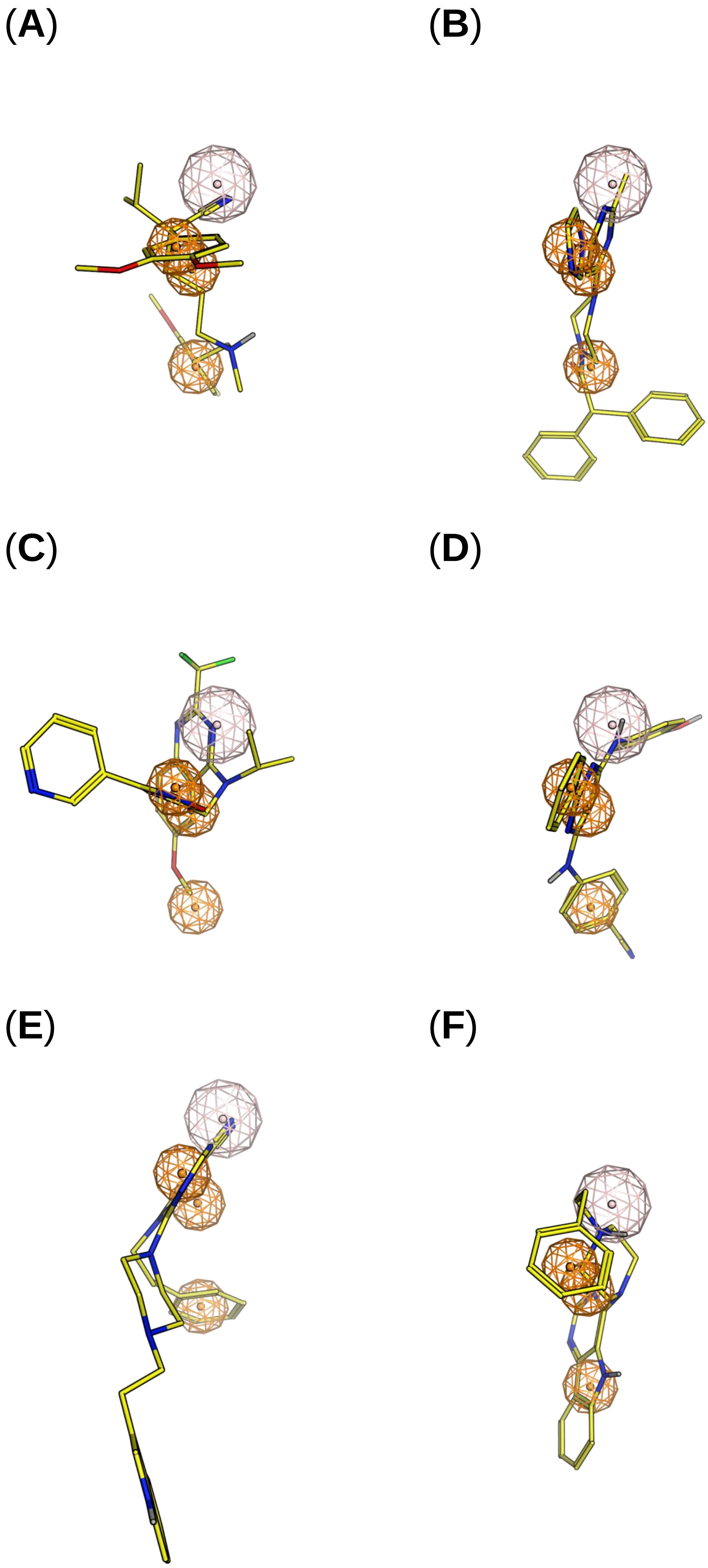
(A)
(B)
(C)
(D)
(E)
(F)

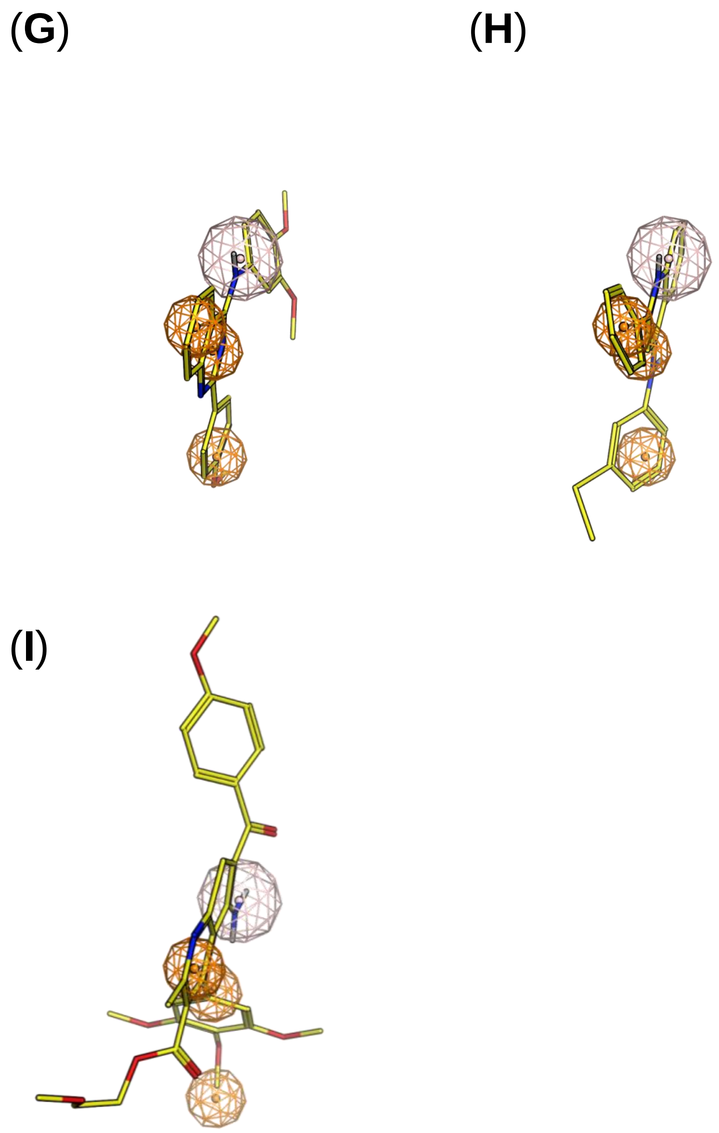

Supplementary Figure S6. The binding poses of 9 of the 10 selected pan-ABC transporter inhibitors [**9** (**A**), **11** (**B**), **14** (**C**), **17** (**D**), **22** (**E**), **23** (**F**), **24** (**G**), **25** (**H**), and **26** (**I**)] [38, 45, 101, 134-137] (colored yellow, stick representation) obtained from Glide [141, 142] screened against the developed pharmacophore model generated from the top ranking docking poses as obtained from Autodock [129]. The best fitting individual molecule is shown superimposed with the four pharmacophore features F1–F2 (aromatic/hydrophobic; orange), F3 (aromatic, orange), and F4 (acceptor/donor; silver). Nonpolar hydrogen atoms were omitted, and polar hydrogen, carbon, nitrogen, oxygen, as well as sulfur atoms were colored in silver, yellow, blue, red, and dark yellow, respectively.

(A)

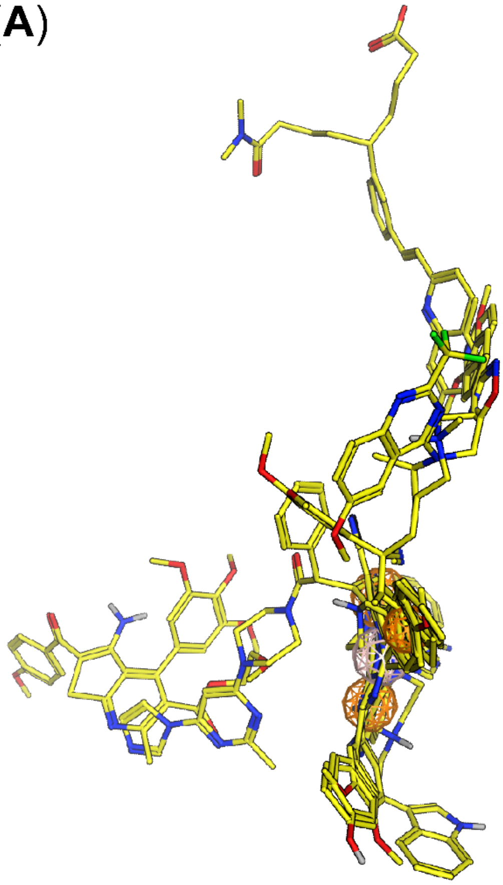

(B)

Features		Distance (Å)
F1	F2	5.12
F1	F3	6.07
F1	F4	3.82
F2	F3	4.39
F2	F4	2.68
F4	F5	2.56

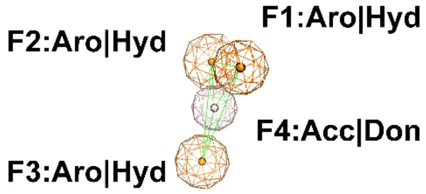

Supplementary Figure S7. Pharmacophore model using the top ranking docking poses of compounds **9–11**, **14**, **17**, and **22–26** [38, 45, 101, 134-137] obtained from Glide [150-151]. (**A**) Superimposed top ranking poses of the docked compounds **9–11**, **14**, **17**, and **22–26** [38, 45, 101, 134-137] from which the four pharmacophore features F1–F4 could be deduced (colored yellow, stick representation). Nonpolar hydrogen atoms were omitted, and polar hydrogen, carbon, nitrogen, oxygen, as well as sulfur atoms were colored in silver white, yellow, blue, red, and dark yellow, respectively. (**B**) The four pharmacophore features F1–F3 (aromatic/hydrophobic) and F4 (acceptor/donor) are depicted in orange (F1–F3) as well as silver (F4). The distances between the individual features are indicated as light green lines and are outlined in the table.

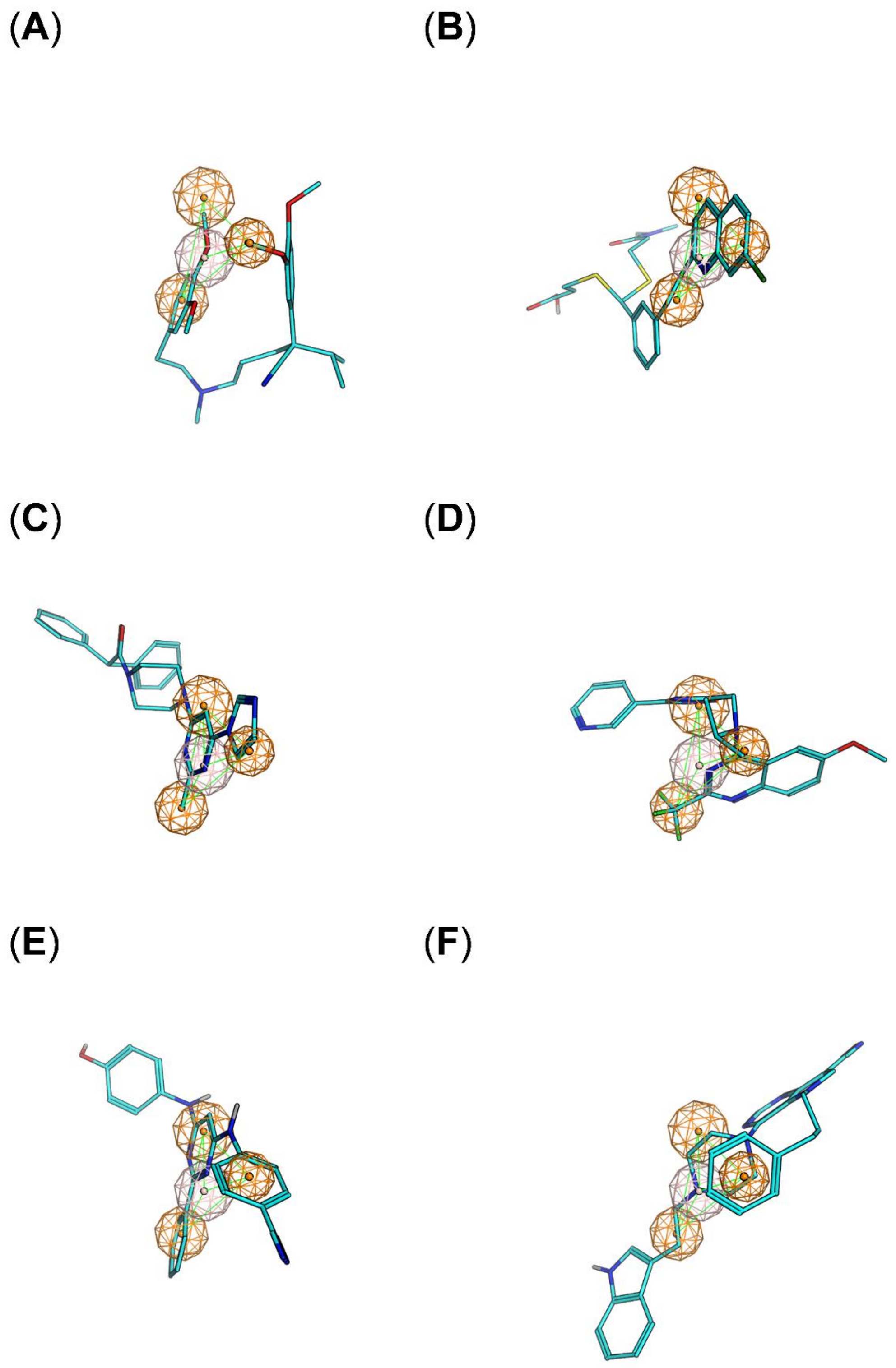
(A)
(B)
(C)
(D)
(E)
(F)

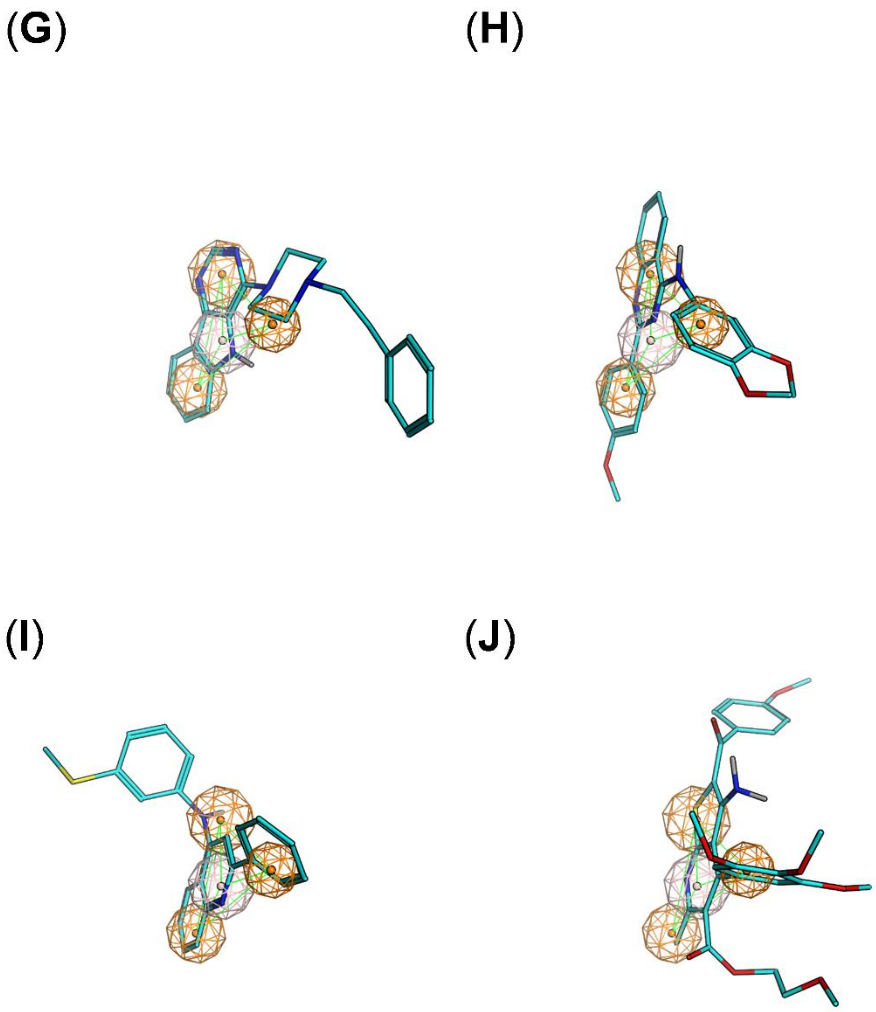

Supplementary Figure S8. The binding poses of the 10 selected pan-ABC transporter inhibitors **9** (**A**), **10** (**B**), **11** (**C**), **14** (**D**), **17** (**E**), **22** (**F**), **23** (**G**), **24** (**H**), **25** (**I**), and **26** (**J**) [38, 45, 101, 134-137] (colored cyan, stick representation) obtained from AutoDock [129] screened against the developed pharmacophore model generated from the top ranking binding poses as obtained from Glide [150-151]. The best fitting individual molecule is shown superimposed with the four pharmacophore features F1–F3 (aromatic/hydrophobic; orange) and F4 (acceptor/donor; silver). Nonpolar hydrogen atoms were omitted, and polar hydrogen, carbon, nitrogen, oxygen, as well as sulfur atoms were colored in silver, cyan, blue, red, and dark yellow, respectively.

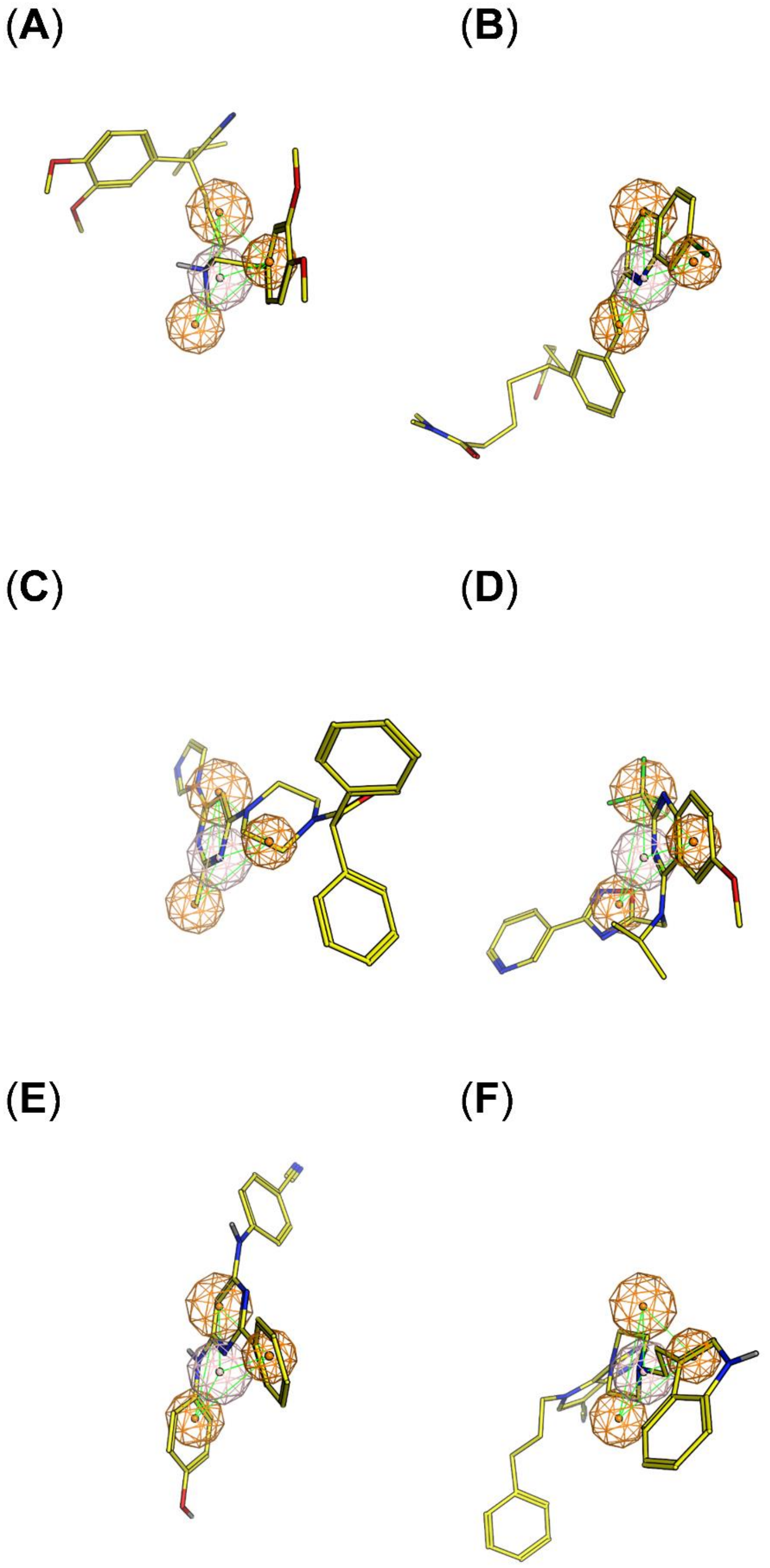

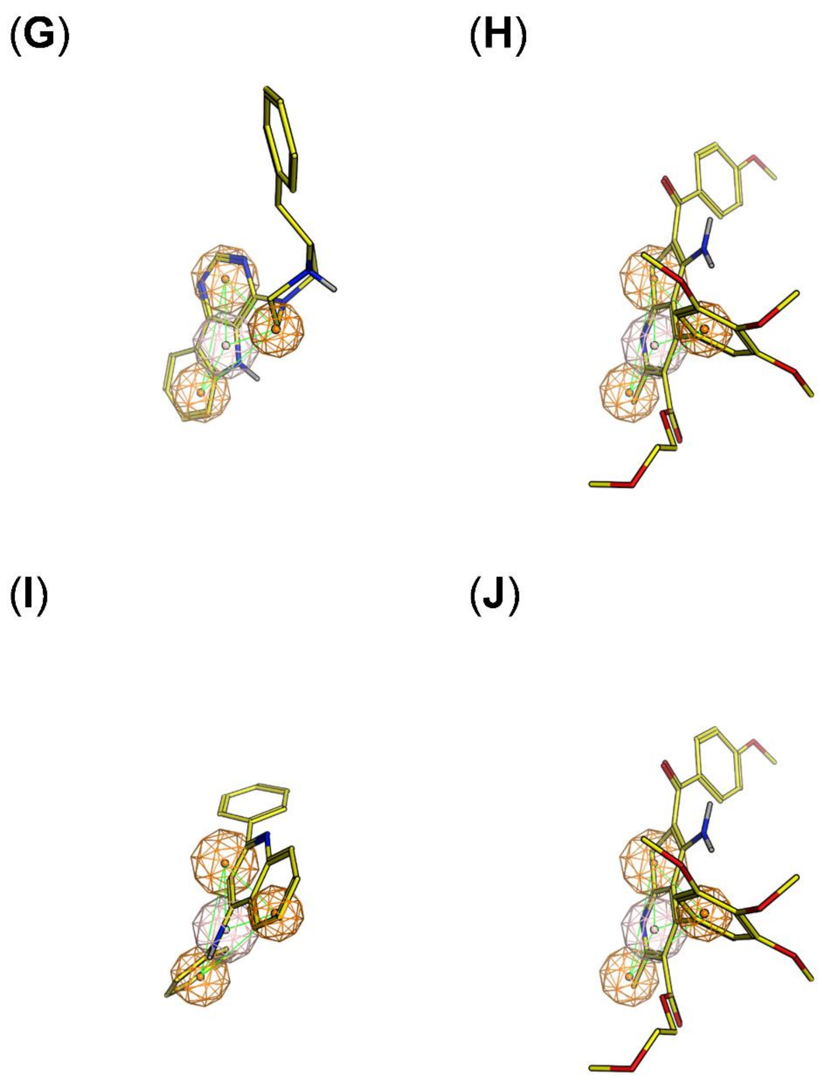

Supplementary Figure S9. The binding poses of the 10 selected pan-ABC transporter inhibitors **9** (**A**), **10** (**B**), **11** (**C**), **14** (**D**), **17** (**E**), **22** (**F**), **23** (**G**), **24** (**H**), **25** (**I**), and **26** (**J**) [38, 45, 101, 134-137] (colored yellow, stick representation) obtained from Glide [150-151] screened against the developed pharmacophore model generated from the top ranking binding poses as obtained from Glide [150-151]. The best fitting individual molecule is shown superimposed with the four pharmacophore features F1–F3 (aromatic/hydrophobic; orange) and F4 (acceptor/donor; silver). Nonpolar hydrogen atoms were omitted, and polar hydrogen, carbon, nitrogen, oxygen, as well as sulfur atoms were colored in silver, yellow, blue, red, and dark yellow, respectively.

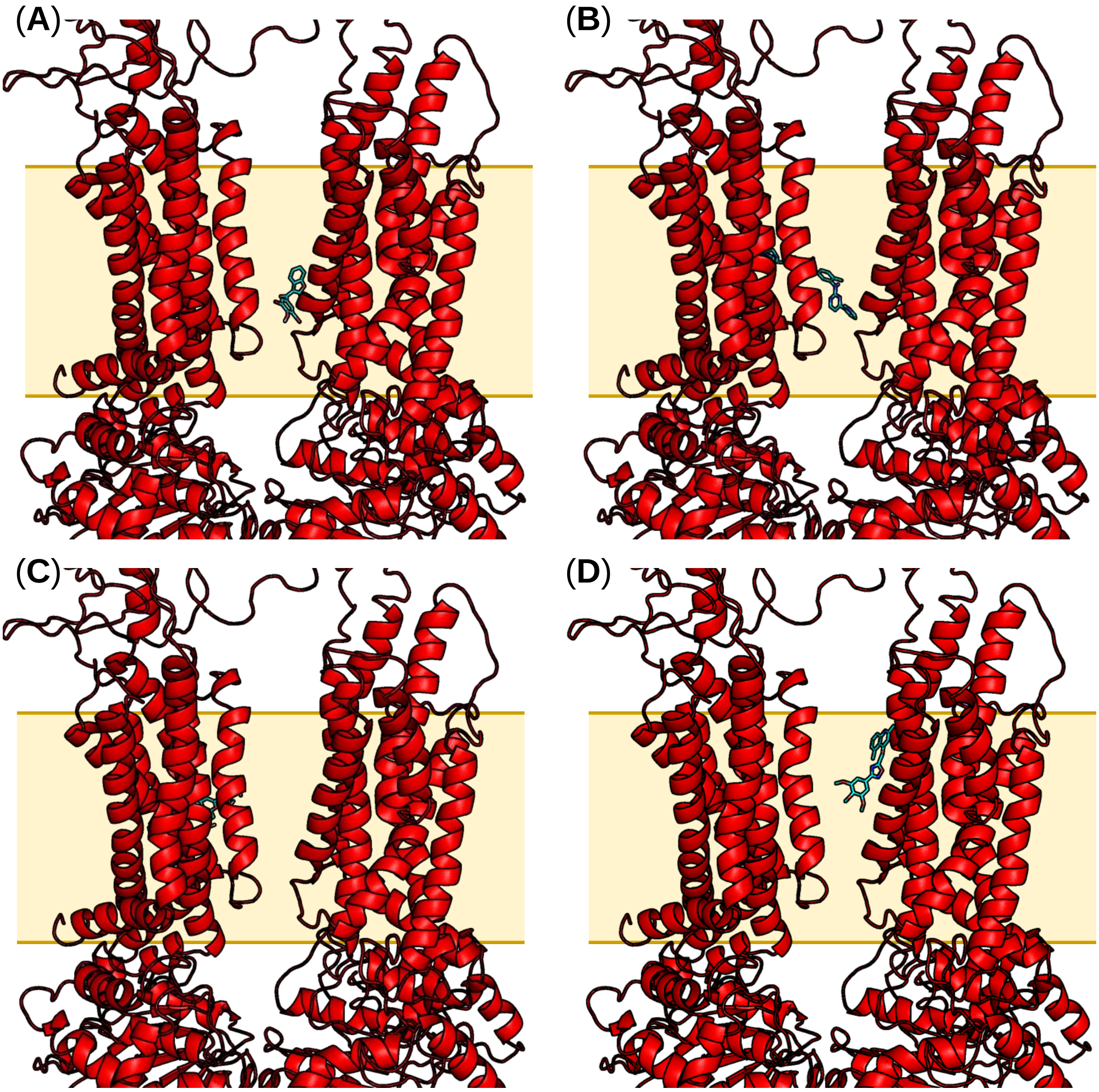
(A)
(B)
(C)
(D)

(E) (F)

(G) (H)

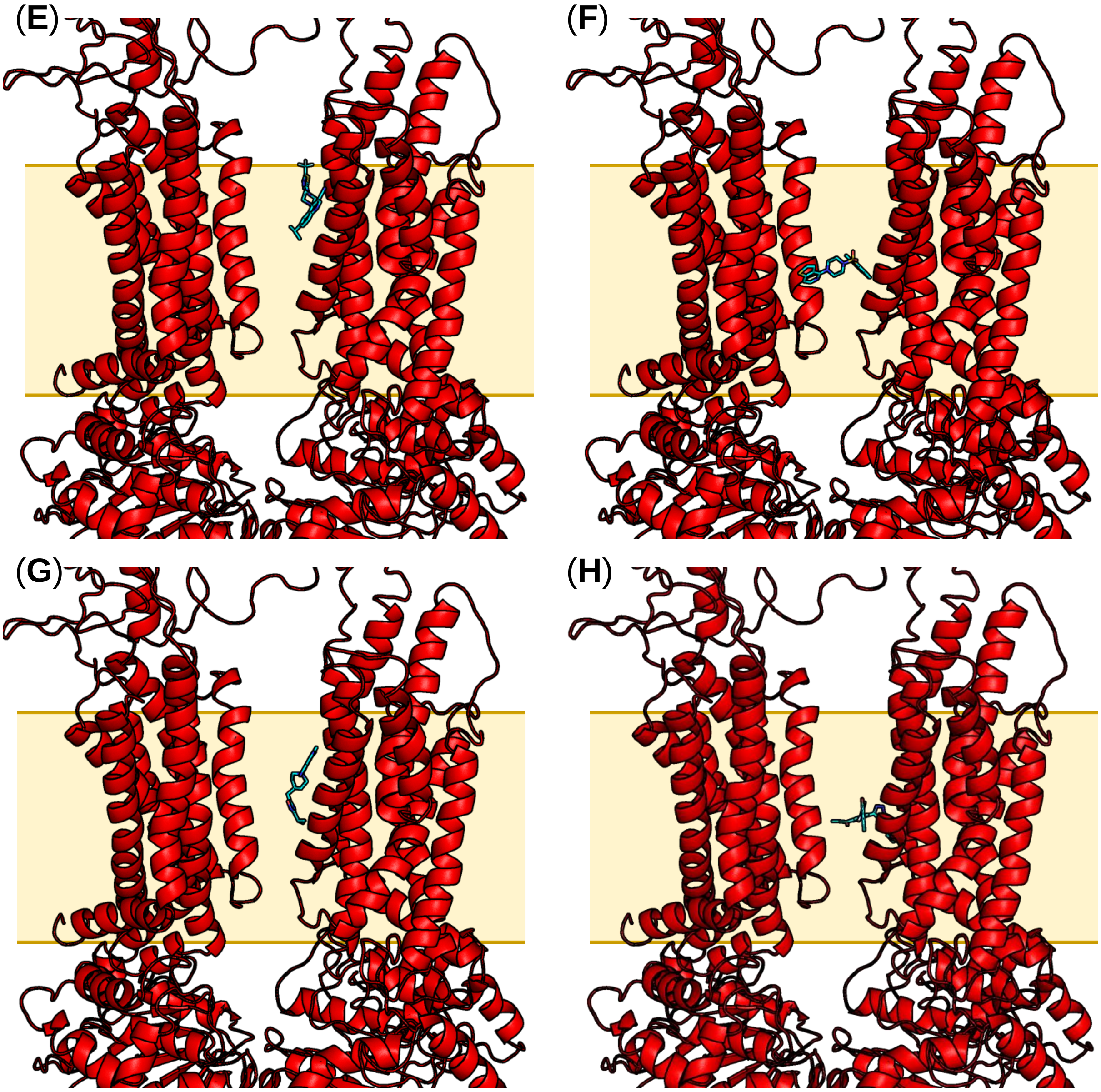

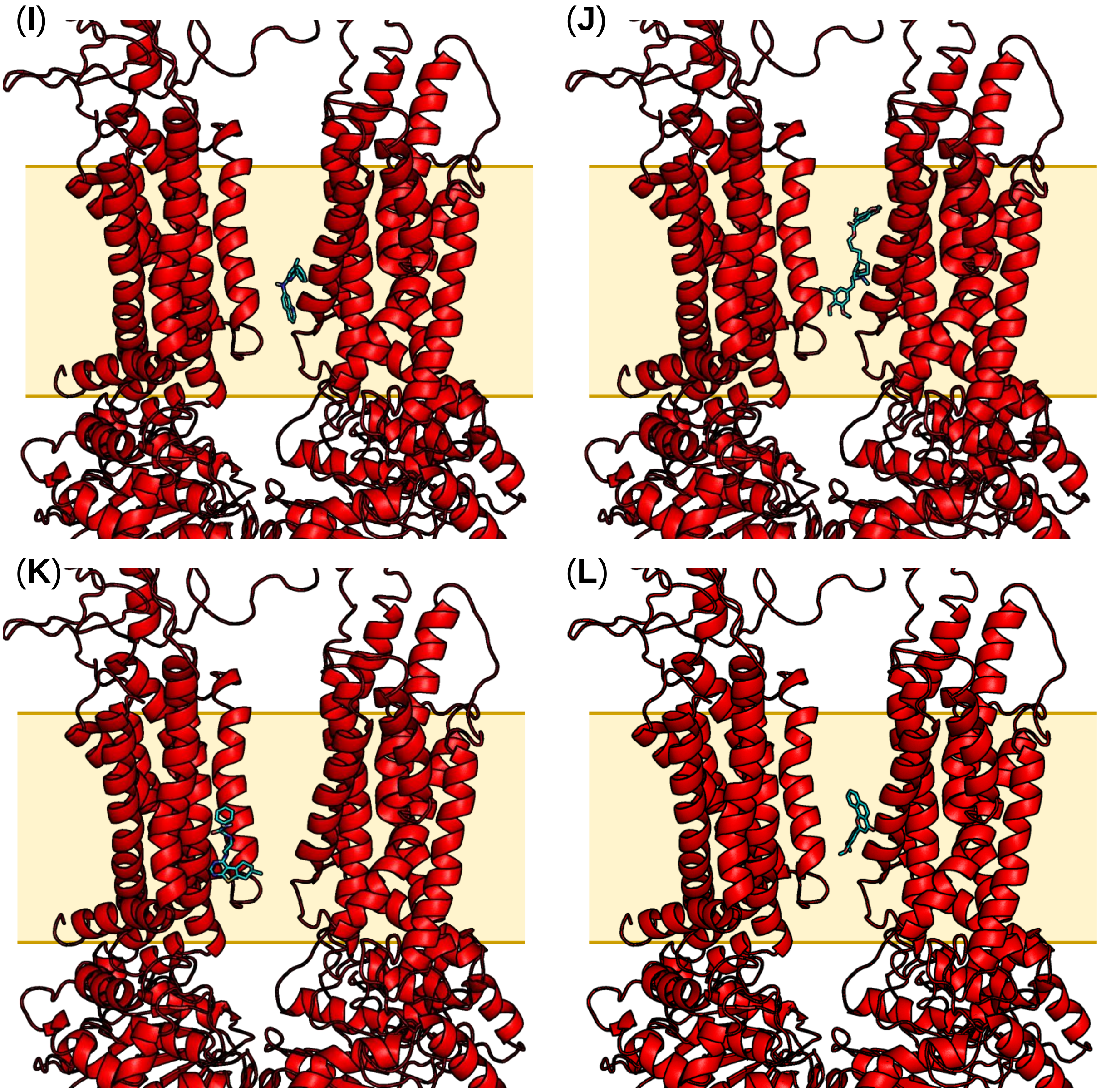
(I)
(J)
(K)
(L)

(M)

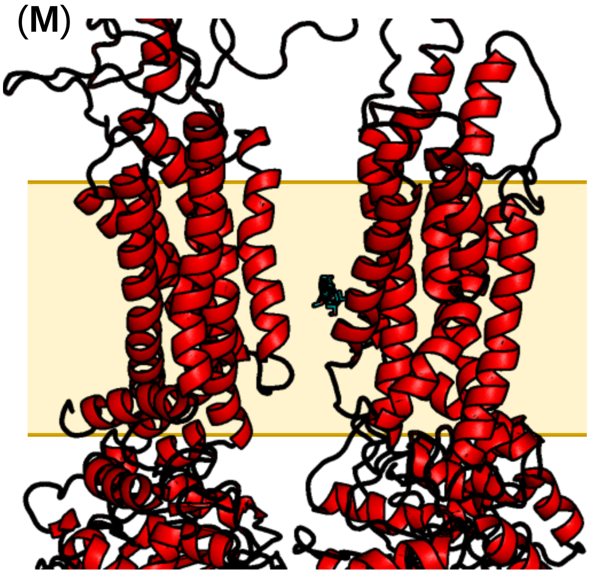

Supplementary Figure S10. The top ranking poses of the 13 docked pan-ABC transporter inhibitors **6** (**A**), **7** (**B**), **8** (**C**), **12** (**D**), **13** (**E**), **15** (**F**), **16** (**G**), **18** (**H**), **19** (**I**), **20** (**J**), **21** (**K**), **27** (**L**), and **28** (**M**) [45, 130-133, 138-140] (colored cyan, stick representation) in the homology model of ABCA7 (colored red, cartoon representation; the inter-membrane space is indicated as light brown area, and the border to the cytosol and lumen is indicated by brown lines) using Autodock [129]. Nonpolar hydrogen atoms were omitted, and polar hydrogen, carbon, nitrogen, oxygen, as well as sulfur atoms were colored in silver, cyan, blue, red, and dark yellow, respectively.

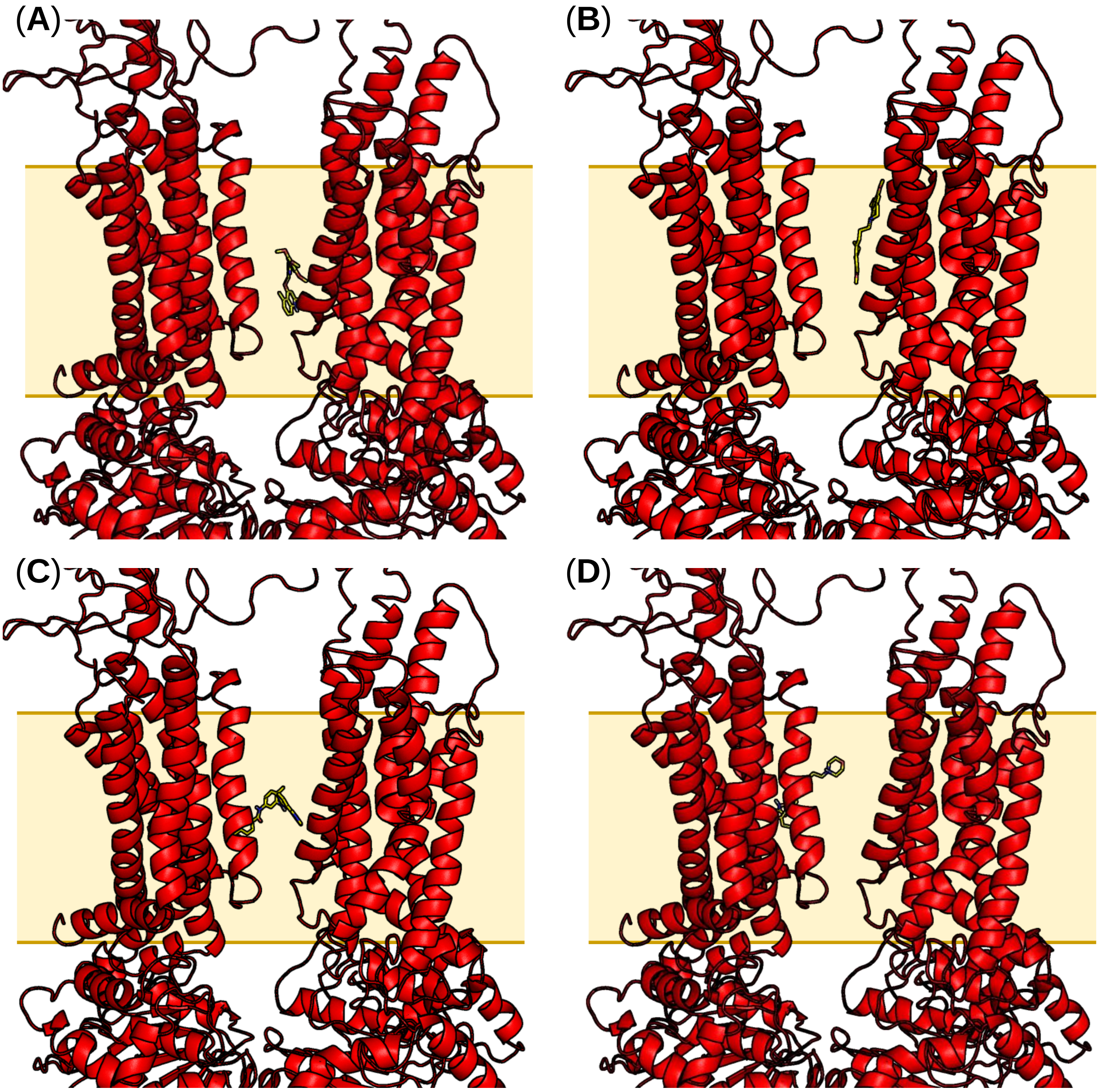
(A)
(B)
(C)
(D)

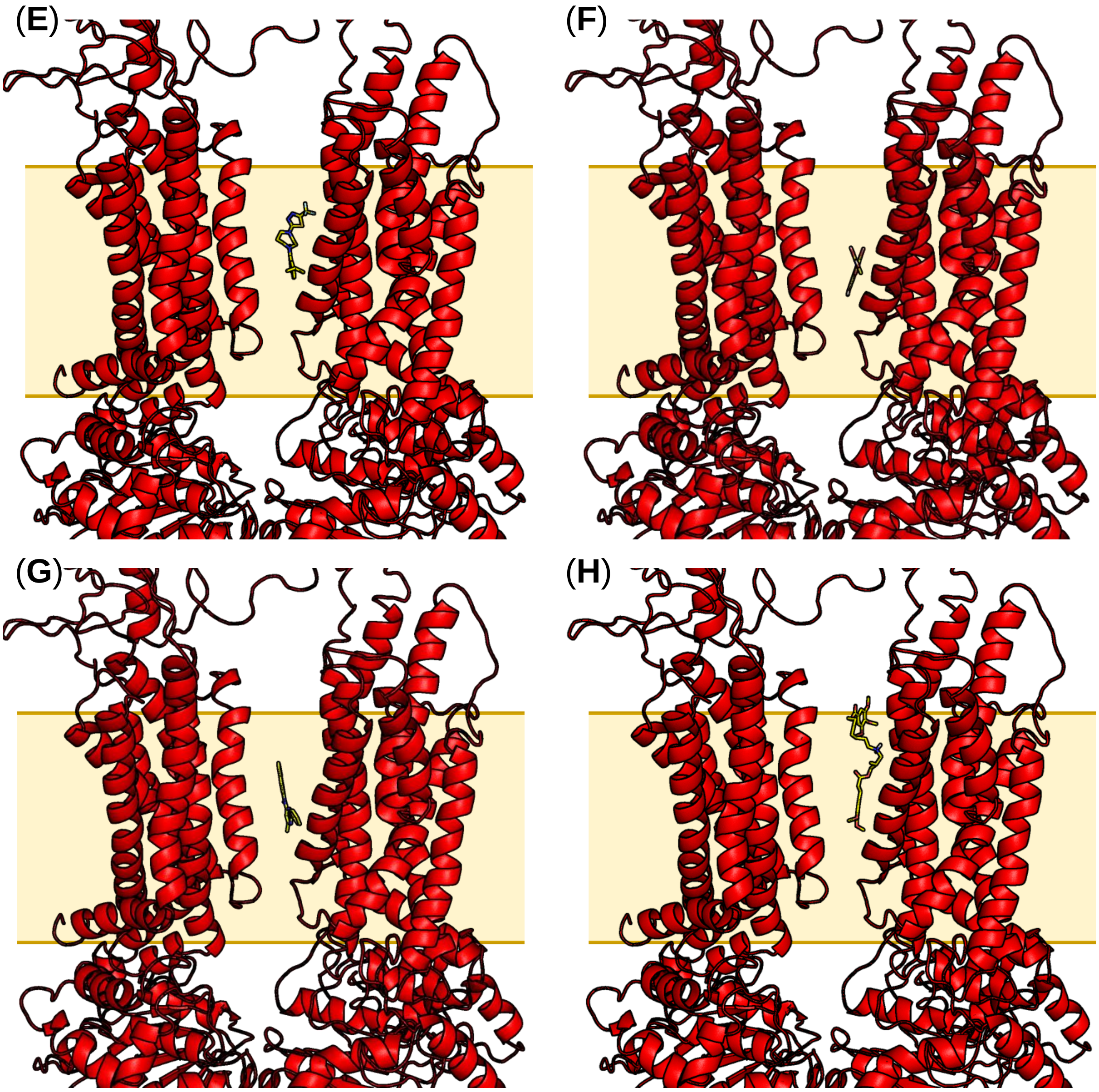
(E)
(F)
(G)
(H)

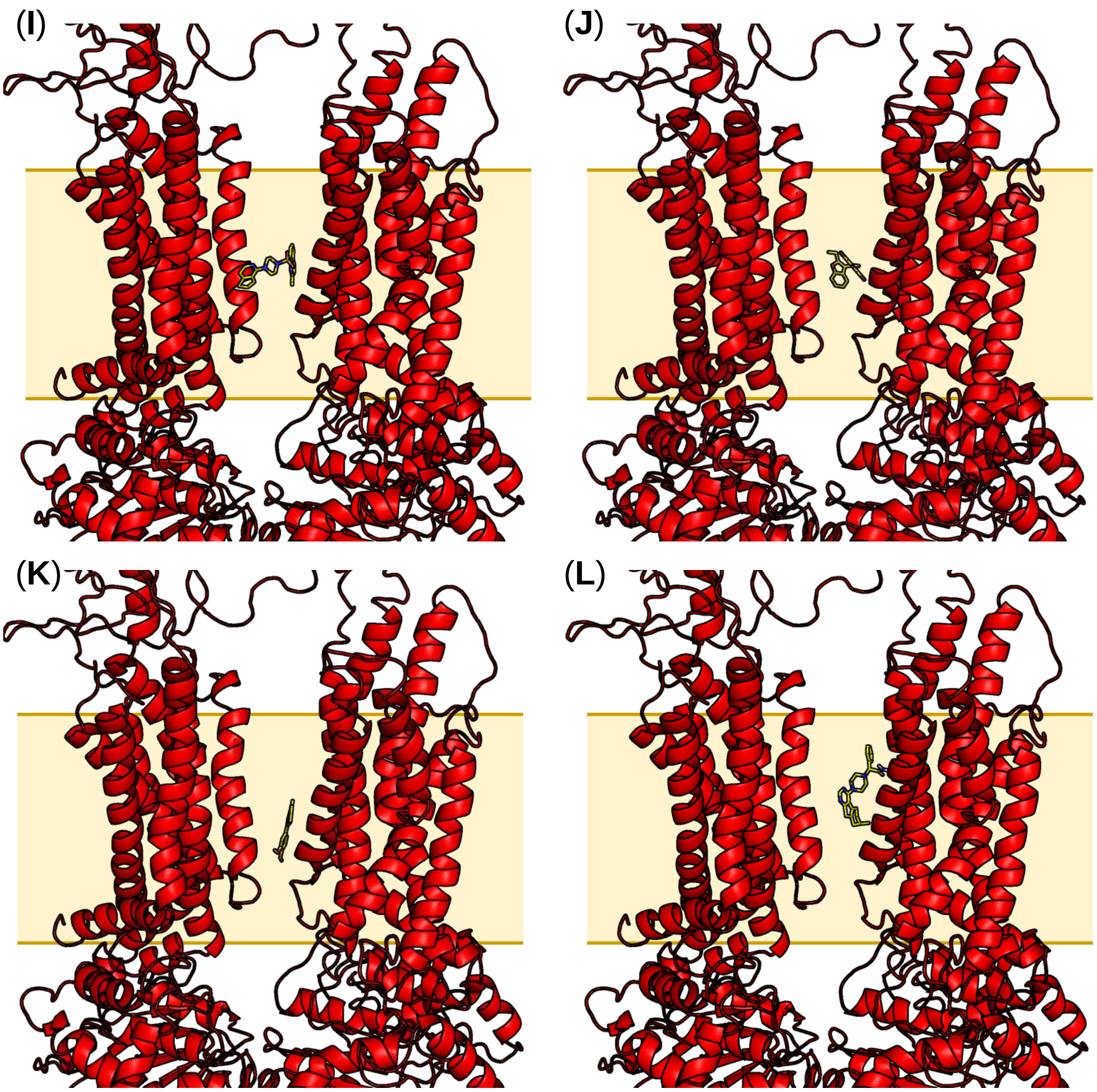
(I)
(J)
(K)
(L)

(M)

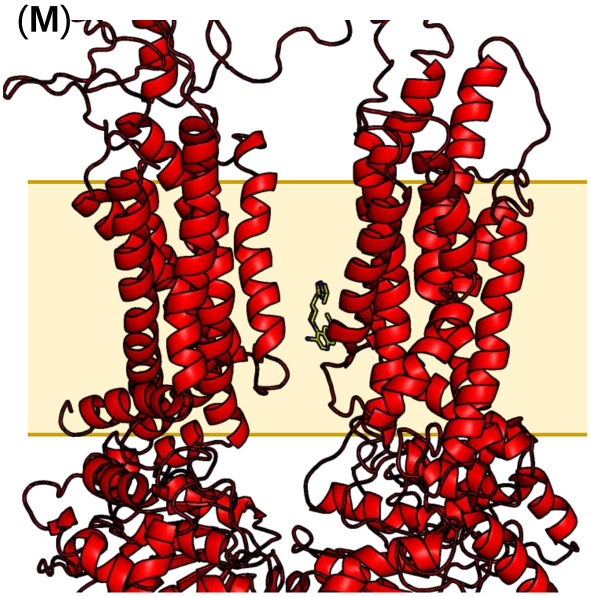

Supplementary Figure S11. The top ranking poses of the 13 docked pan-ABC transporter inhibitors **6** (**A**), **7** (**B**), **8** (**C**), **12** (**D**), **13** (**E**), **15** (**F**), **16** (**G**), **18** (**H**), **19** (**I**), **20** (**J**), **21** (**K**), **27** (**L**), and **28** (**M**) [45, 130-133, 138-140] (colored yellow, stick representation) in the homology model of ABCA7 (colored red, cartoon representation; the inter-membrane space is indicated as light brown area, and the border to the cytosol and lumen is indicated by brown lines) using Glide [141, 142]. Nonpolar hydrogen atoms were omitted, and polar hydrogen, carbon, nitrogen, oxygen, as well as sulfur atoms were colored in silver, yellow, blue, red, and dark yellow, respectively.

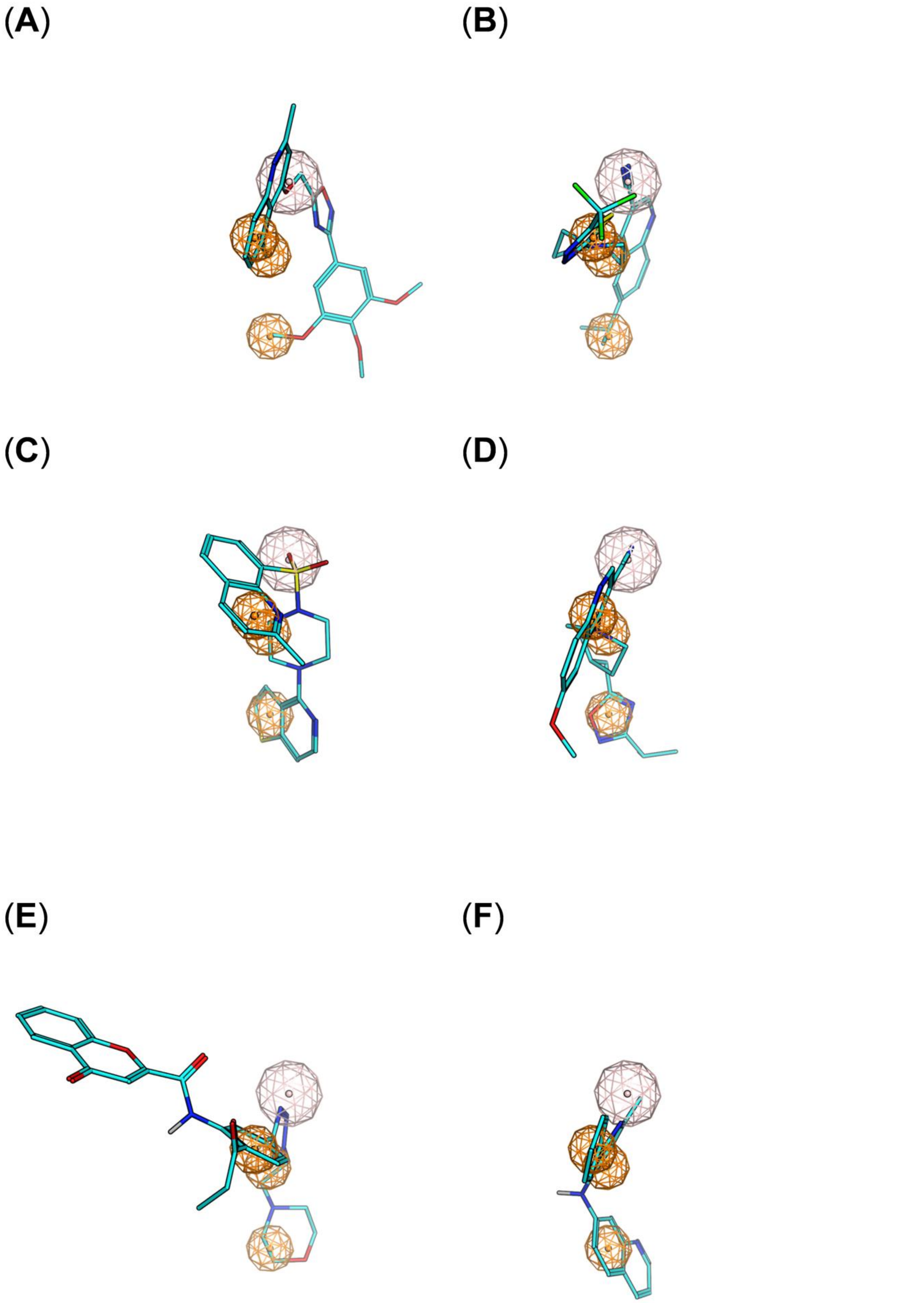
(A)
(B)
(C)
(D)
(E)
(F)

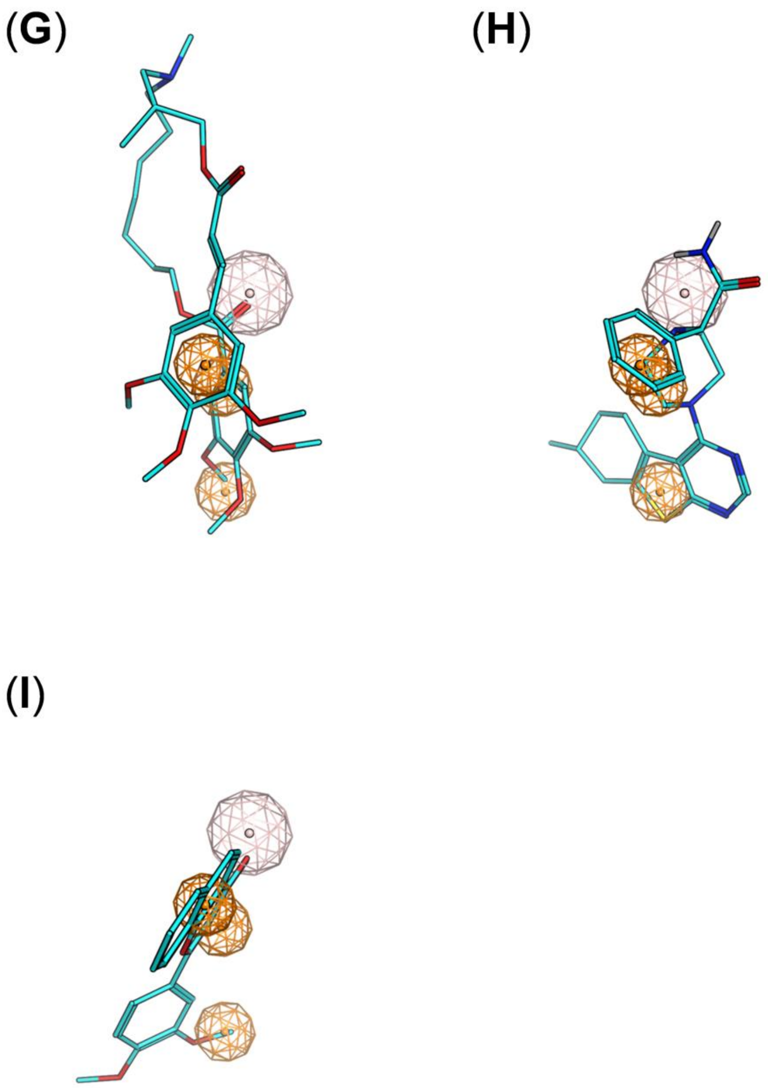

Supplementary Figure S12. The binding poses of 9 of the 13 pan-ABC transporter inhibitors [**12** (**A**), **13** (**B**), **15** (**C**), **16** (**D**), **18** (**E**), **19** (**F**), **20** (**G**), **21** (**H**), **27** (**I**)] [45, 130-133, 138] (colored cyan, stick representation) obtained from Autodock [129] screened against the developed pharmacophore model generated from the top ranking docking poses as obtained from Autodock [129]. The best fitting individual molecule is shown superimposed with the four pharmacophore features F1–F2 (aromatic/hydrophobic; orange), F3 (aromatic, orange), and F4 (acceptor/donor; silver). Nonpolar hydrogen atoms were omitted, and polar hydrogen, carbon, nitrogen, oxygen, as well as sulfur atoms were colored in silver, cyan, blue, red, and dark yellow, respectively.

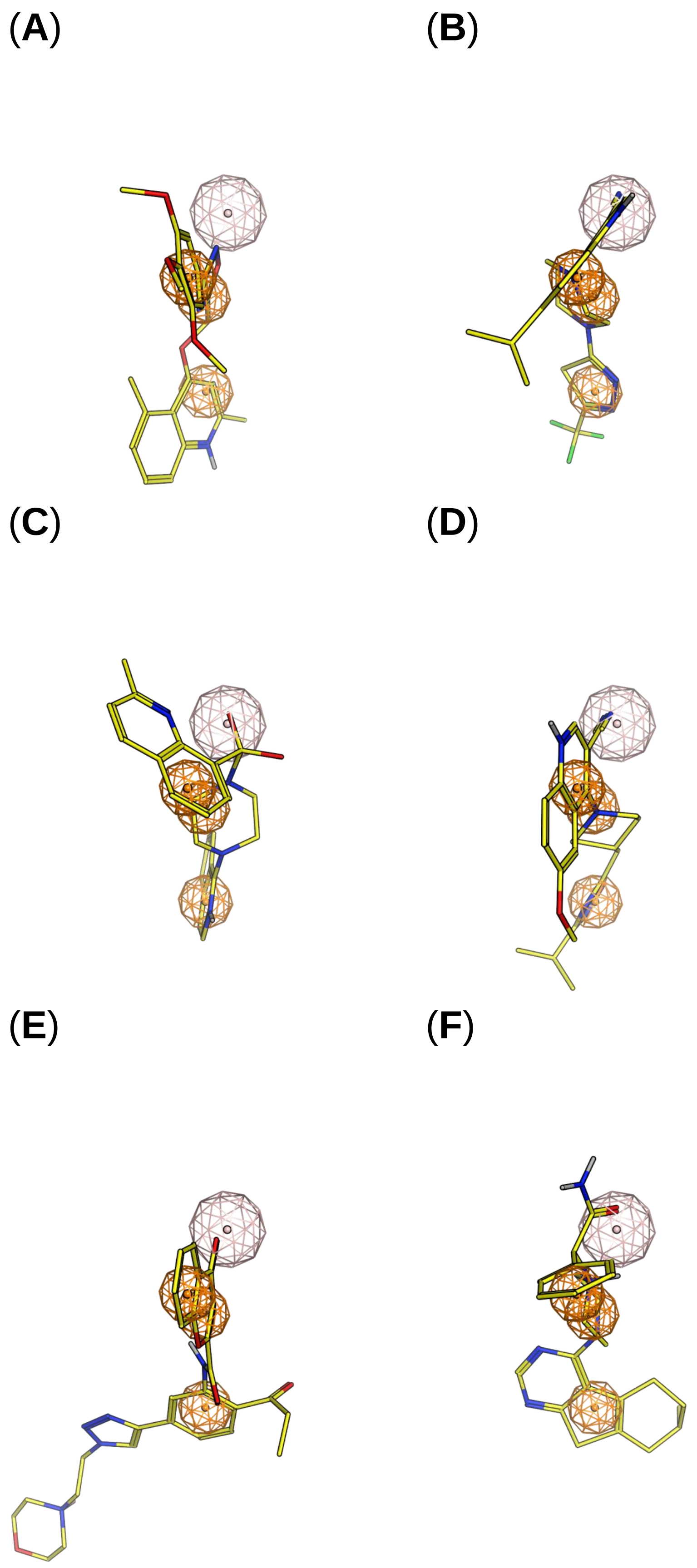
(A)
(B)
(C)
(D)
(E)
(F)

(**G**)

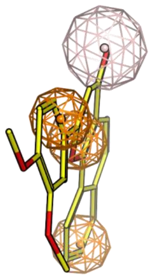

Supplementary Figure S13. The binding poses of 7 of the 13 pan-ABC transporter inhibitors [**12** (**A**), **13** (**B**), **15** (**C**), **16** (**D**), **18** (**E**), **21** (**F**), **27** (**G**)] [45, 130, 133, 138] (colored yellow, stick representation) obtained from Glide [141, 142] screened against the developed pharmacophore model generated from the top ranking docking poses as obtained from Autodock [129]. The best fitting individual molecule is shown superimposed with the four pharmacophore features F1–F2 (aromatic/hydrophobic; orange), F3 (aromatic, orange), and F4 (acceptor/donor; silver). Nonpolar hydrogen atoms were omitted, and polar hydrogen, carbon, nitrogen, oxygen, as well as sulfur atoms were colored in silver, yellow, blue, red, and dark yellow, respectively.

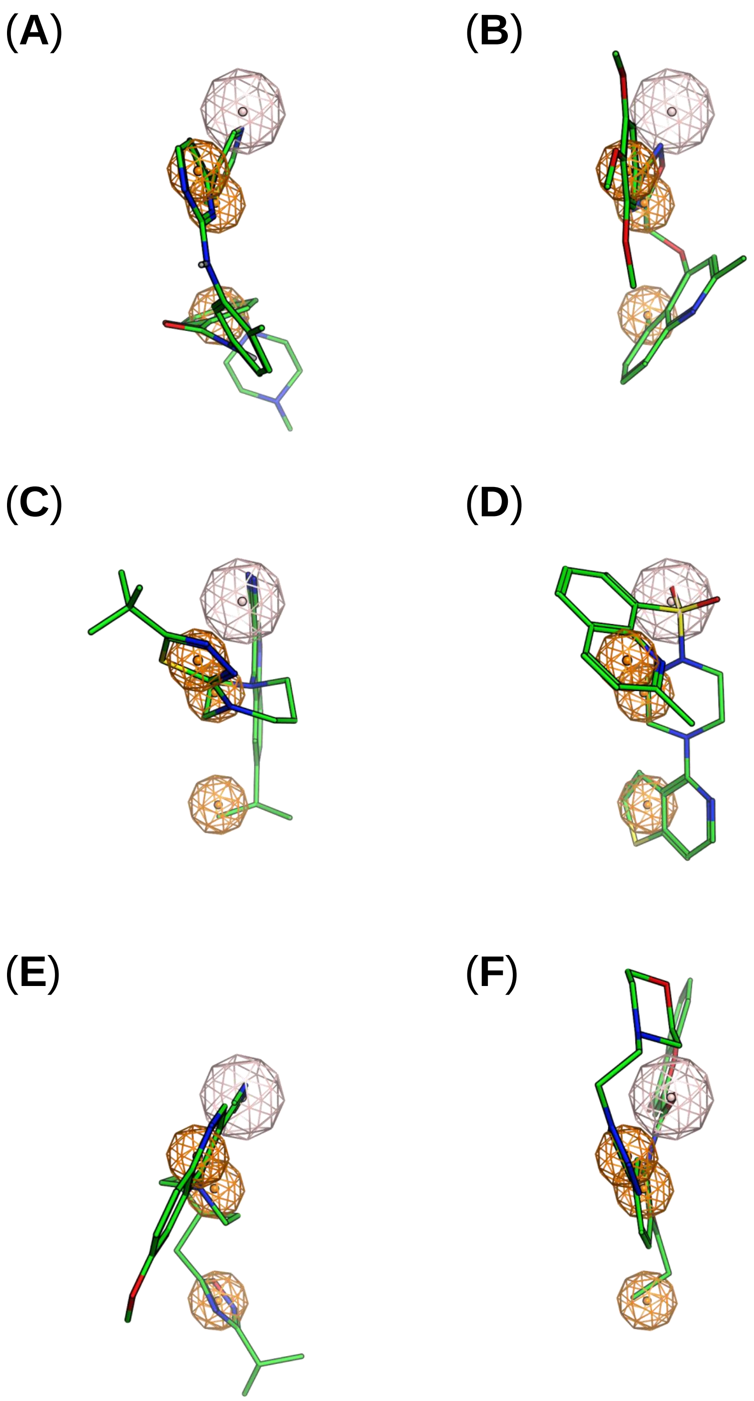
(A)
(B)
(C)
(D)
(E)
(F)

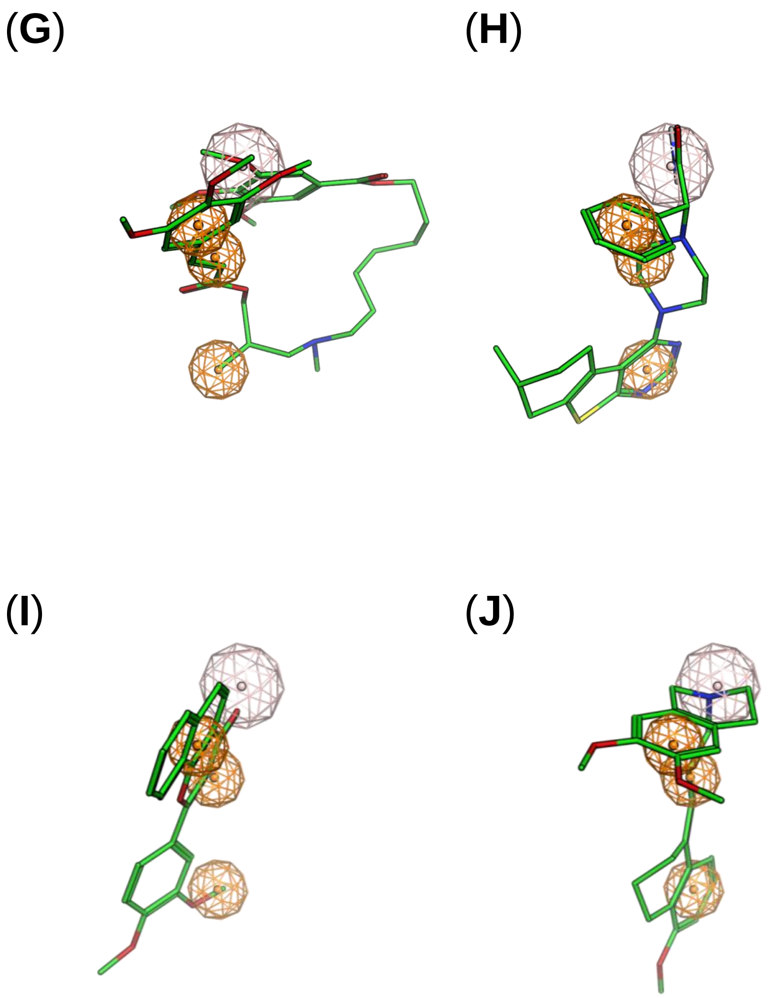

Supplementary Figure S14. The binding poses of 10 of the 13 pan-ABC transporter inhibitors [**7** (**A**), **12** (**B**), **13** (**C**), **15** (**D**), **16** (**E**), **18** (**F**), **20** (**G**), **21** (**H**), **27** (**I**), and **28** (**J**)] [45, 130-133, 138-140] (colored green, stick representation) obtained from the conformer generation tool implemented in MOE 2019.01 [118] screened against the developed pharmacophore model generated from the top ranking docking poses as obtained from Autodock [149]. The best fitting individual molecule is shown superimposed with the four pharmacophore features F1–F2 (aromatic/hydrophobic; orange), F3 (aromatic, orange), and F4 (acceptor/donor; silver). Nonpolar hydrogen atoms were omitted, and polar hydrogen, carbon, nitrogen, oxygen, as well as sulfur atoms were colored in silver, cyan, blue, red, and dark yellow, respectively.

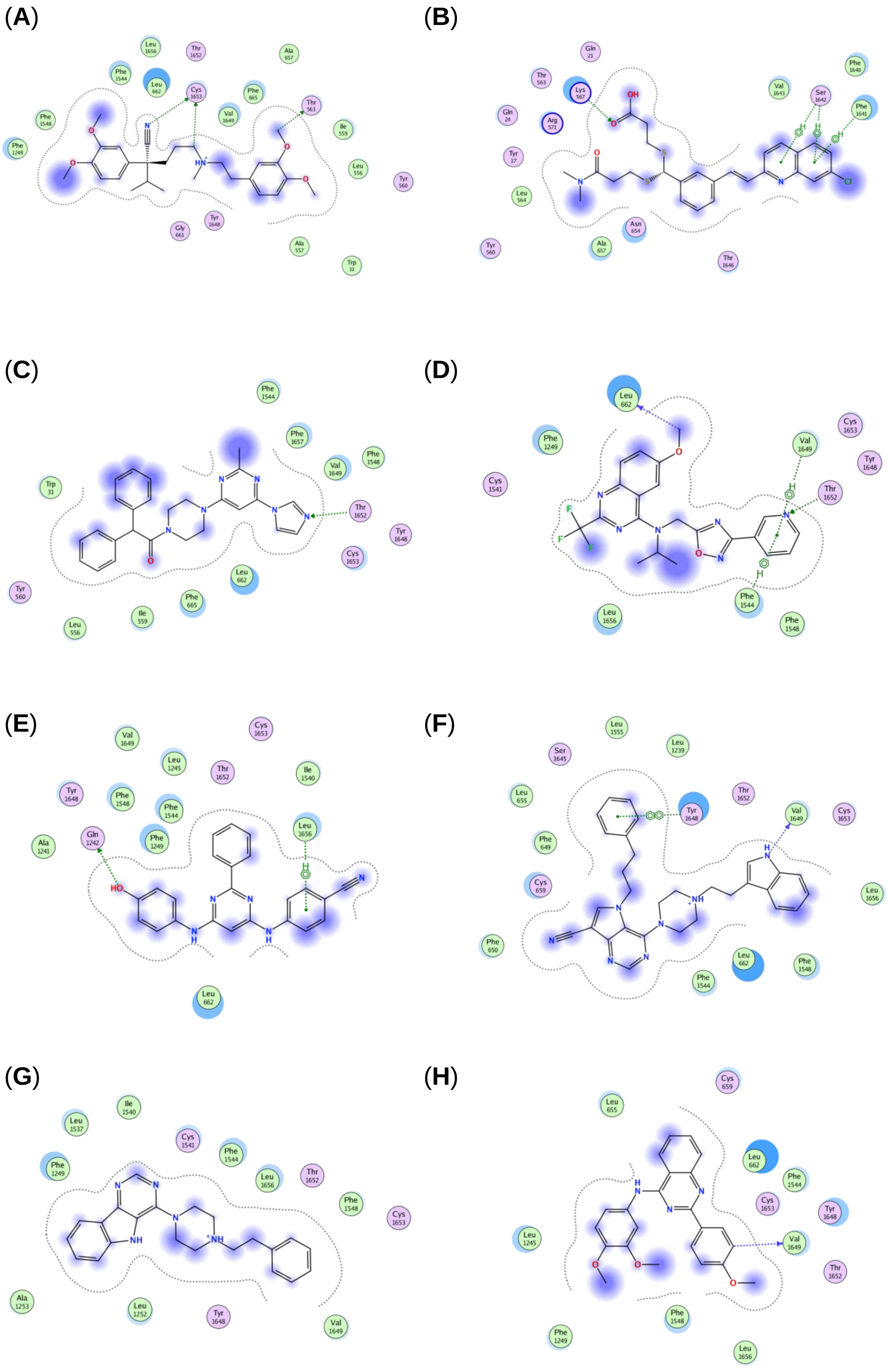
(A)
Leu 1656
Thr 1652
Ala 657
Phe 1544
Leu 662
Cys 1653
Phe 665
Val 1649
Thr 563
Phe 1548
Ile 559
Phe 1249
Leu 556
Tyr 560
Gly 661
Tyr 1648
Ala 557
Trp 31
(B)
Gln 21
Thr 563
Lys 567
Phe 1640
Val 1643
Ser 1642
Phe 1641
Gln 24
Arg 571
Tyr 17
Leu 564
Asn 654
Tyr 560
Ala 657
Thr 1646
(C)
Phe 1544
Phe 1657
Phe 1548
Val 1649
Trp 31
Thr 1652
Tyr 1648
Cys 1653
Leu 662
Tyr 560
Phe 665
Ile 559
Leu 556
(D)
Leu 662
Cys 1653
Phe 1249
Val 1649
Tyr 1648
Cys 1541
Thr 1652
Phe 1544
Leu 1656
Phe 1548
(E)
Val 1649
Cys 1653
Leu 1245
Thr 1652
Ile 1540
Tyr 1648
Phe 1548
Phe 1544
Leu 1656
Ala 1241
Gln 1242
Phe 1249
Leu 662
(F)
Leu 1555
Leu 1239
Ser 1645
Thr 1652
Leu 655
Tyr 1648
Val 1649
Cys 1653
Phe 649
Cys 659
Leu 1656
Phe 650
Leu 662
Phe 1548
Phe 1544
(G)
Ile 1540
Leu 1537
Cys 1541
Phe 1544
Phe 1249
Leu 1656
Thr 1652
Phe 1548
Cys 1653
Ala 1253
Leu 1252
Tyr 1648
Val 1649
(H)
Cys 659
Leu 655
Leu 662
Phe 1544
Cys 1653
Tyr 1648
Leu 1245
Val 1649
Thr 1652
Phe 1548
Phe 1249
Leu 1656

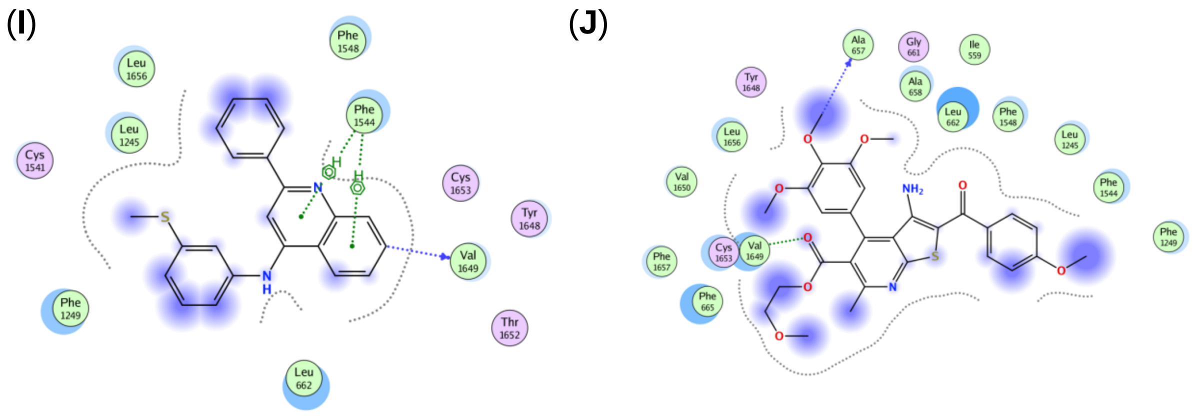

Supplementary Figure S15. 2D interaction diagram of the top ranking poses of the 10 docked pan-ABC transporter inhibitors **9** (**A**), **10** (**B**), **11** (**C**), **14** (**D**), **17** (**E**), **22** (**F**), **23** (**G**), **24** (**H**), **25** (**I**), and **26** (**J**) [38, 45, 101, 134-137] in the homology model of ABCA7 using AutoDock [129]. Nitrogen, oxygen, sulfur, fluorine, chlorine, and bromine atoms were colored in blue, red, dark yellow, light green, green, and dark red, respectively.

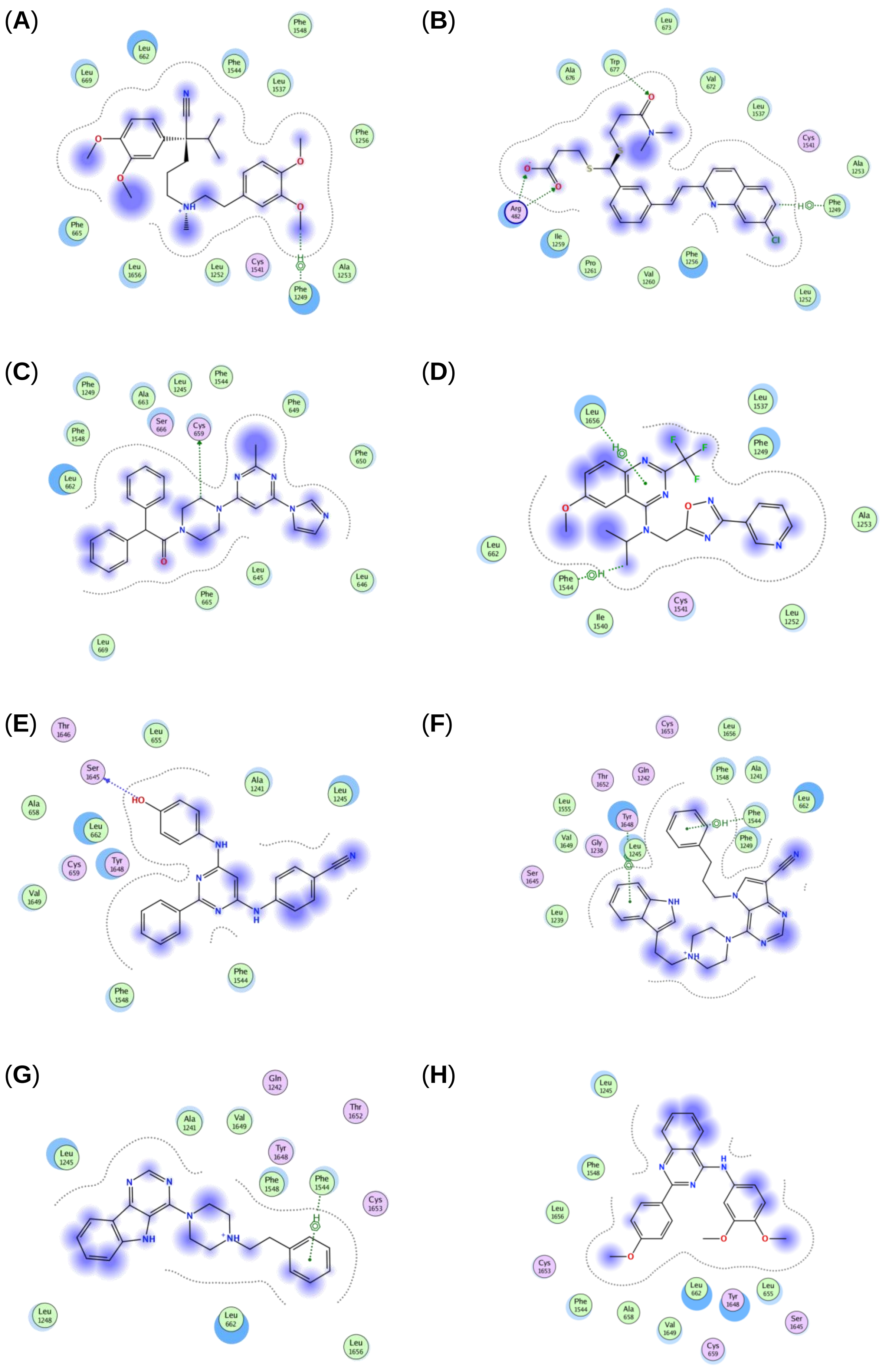
(A)
Phe 1548
Leu 662
Leu 669
Phe 1544
Leu 1537
Phe 1256
Phe 665
Leu 1656
Leu 1252
Cys 1541
Ala 1253
Phe 1249
(B)
Leu 673
Trp 677
Ala 676
Val 672
Leu 1537
Cys 1541
Ala 1253
Arg 482
Phe 1249
Ile 1259
Pro 1261
Val 1260
Phe 1256
Leu 1252
(C)
Phe 1249
Ala 663
Leu 1245
Phe 1544
Phe 649
Ser 666
Cys 659
Phe 1548
Leu 662
Phe 650
Leu 645
Leu 646
Phe 665
Leu 669
(D)
Leu 1656
Leu 1537
Phe 1249
Leu 662
Ala 1253
Phe 1544
Cys 1541
Ile 1540
Leu 1252
(E)
Thr 1646
Leu 655
Ser 1645
Ala 1241
Leu 1245
Ala 658
Leu 662
Cys 659
Tyr 1648
Val 1649
Phe 1544
Phe 1548
(F)
Cys 1653
Leu 1656
Thr 1652
Gln 1242
Phe 1548
Ala 1241
Leu 662
Leu 1555
Tyr 1648
Phe 1544
Val 1649
Gly 1238
Leu 1245
Phe 1249
Ser 1645
Leu 1239
(G)
Gln 1242
Thr 1652
Ala 1241
Val 1649
Leu 1245
Tyr 1648
Phe 1548
Phe 1544
Cys 1653
Leu 1248
Leu 662
Leu 1656
(H)
Leu 1245
Phe 1548
Leu 1656
Cys 1653
Leu 662
Tyr 1648
Leu 655
Phe 1544
Ala 658
Val 1649
Ser 1645
Cys 659

(I)

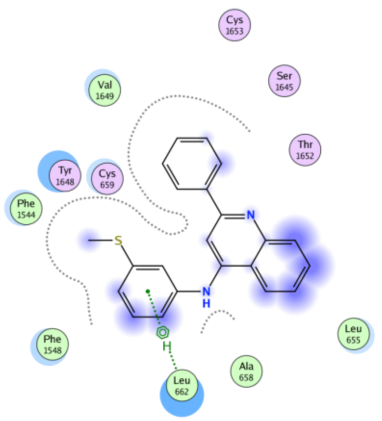

(J)

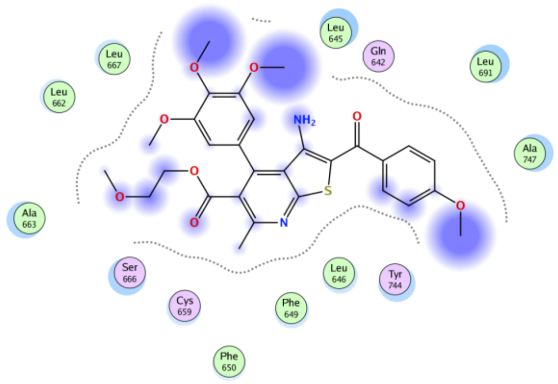

Supplementary Figure S16. 2D interaction diagram of the top ranking poses of the 10 docked pan-ABC transporter inhibitors **9** (**A**), **10** (**B**), **11** (**C**), **14** (**D**), **17** (**E**), **22** (**F**), **23** (**G**), **24** (**H**), **25** (**I**), and **26** (**J**) [38, 45, 101, 134-137] in the homology model of ABCA7 using Glide [141, 142]. Nitrogen, oxygen, sulfur, fluorine, chlorine, and bromine atoms were colored in blue, red, dark yellow, light green, green, and dark red, respectively.

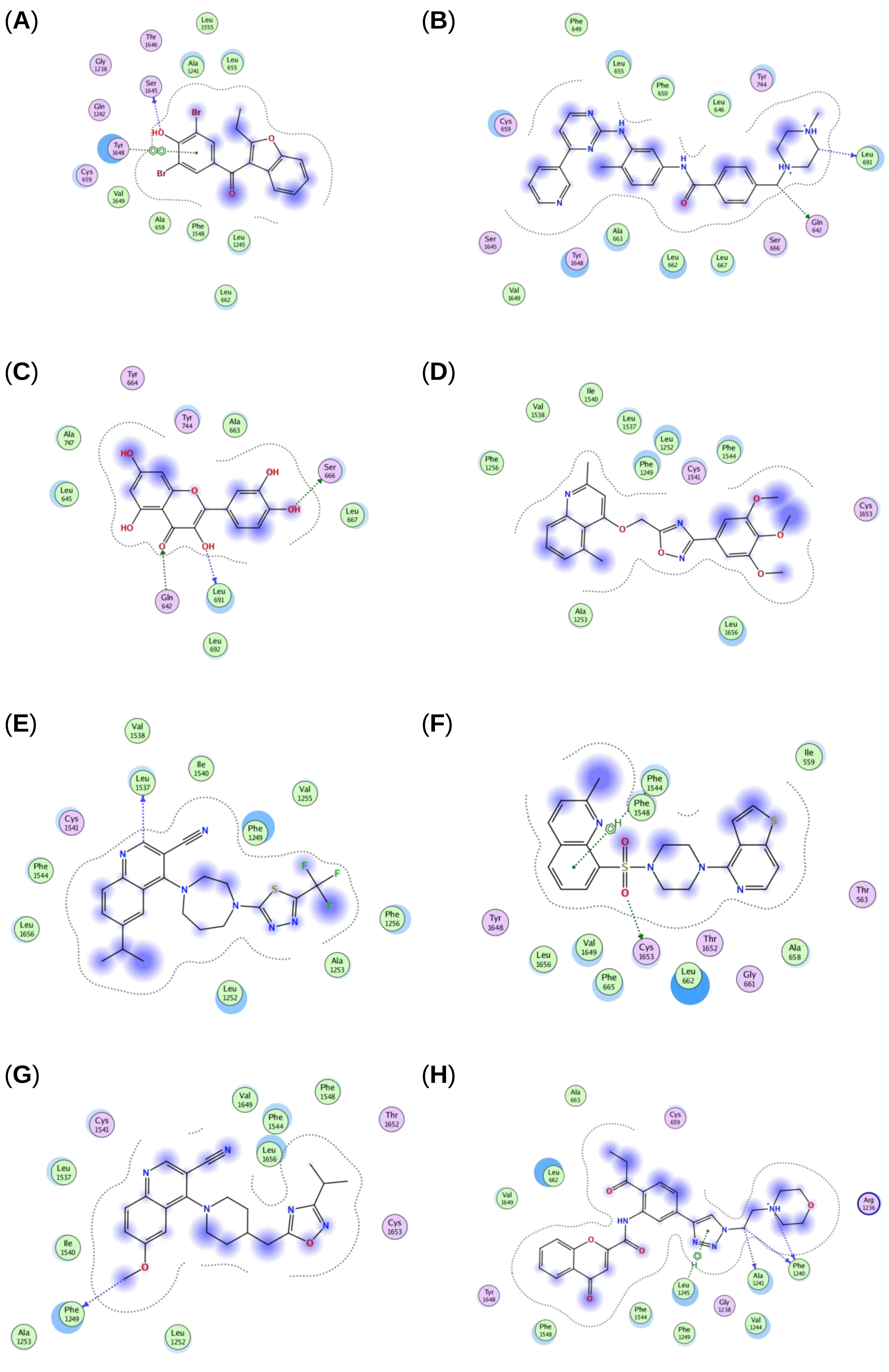
(A)
Leu 1555
Thr 1646
Gly 1238
Ala 1241
Leu 655
Ser 1645
Gln 1242
Tyr 1648
Cys 659
Val 1649
Ala 658
Phe 1548
Leu 1245
Leu 662
(B)
Phe 649
Leu 655
Tyr 744
Phe 650
Leu 646
Cys 659
Leu 691
Gln 642
Ser 1645
Ala 663
Ser 666
Tyr 1648
Leu 662
Leu 667
Val 1649
(C)
Tyr 664
Tyr 744
Ala 663
Ala 747
Ser 666
Leu 645
Leu 667
Gln 642
Leu 691
Leu 692
(D)
Ile 1540
Val 1538
Leu 1537
Leu 1252
Phe 1544
Phe 1256
Phe 1249
Cys 1541
Cys 1653
Ala 1253
Leu 1656
(E)
Val 1538
Ile 1540
Leu 1537
Val 1255
Cys 1541
Phe 1249
Phe 1544
Phe 1256
Leu 1656
Ala 1253
Leu 1252
(F)
Ile 559
Phe 1544
Phe 1548
Thr 563
Tyr 1648
Thr 1652
Val 1649
Cys 1653
Ala 658
Leu 1656
Leu 662
Gly 661
Phe 665
(G)
Val 1649
Phe 1548
Thr 1652
Cys 1541
Phe 1544
Leu 1656
Leu 1537
Cys 1653
Ile 1540
Phe 1249
Ala 1253
Leu 1252
(H)
Ala 663
Cys 659
Leu 662
Val 1649
Arg 1236
Phe 1240
Ala 1241
Tyr 1648
Leu 1245
Gly 1238
Phe 1544
Val 1244
Phe 1548
Phe 1249

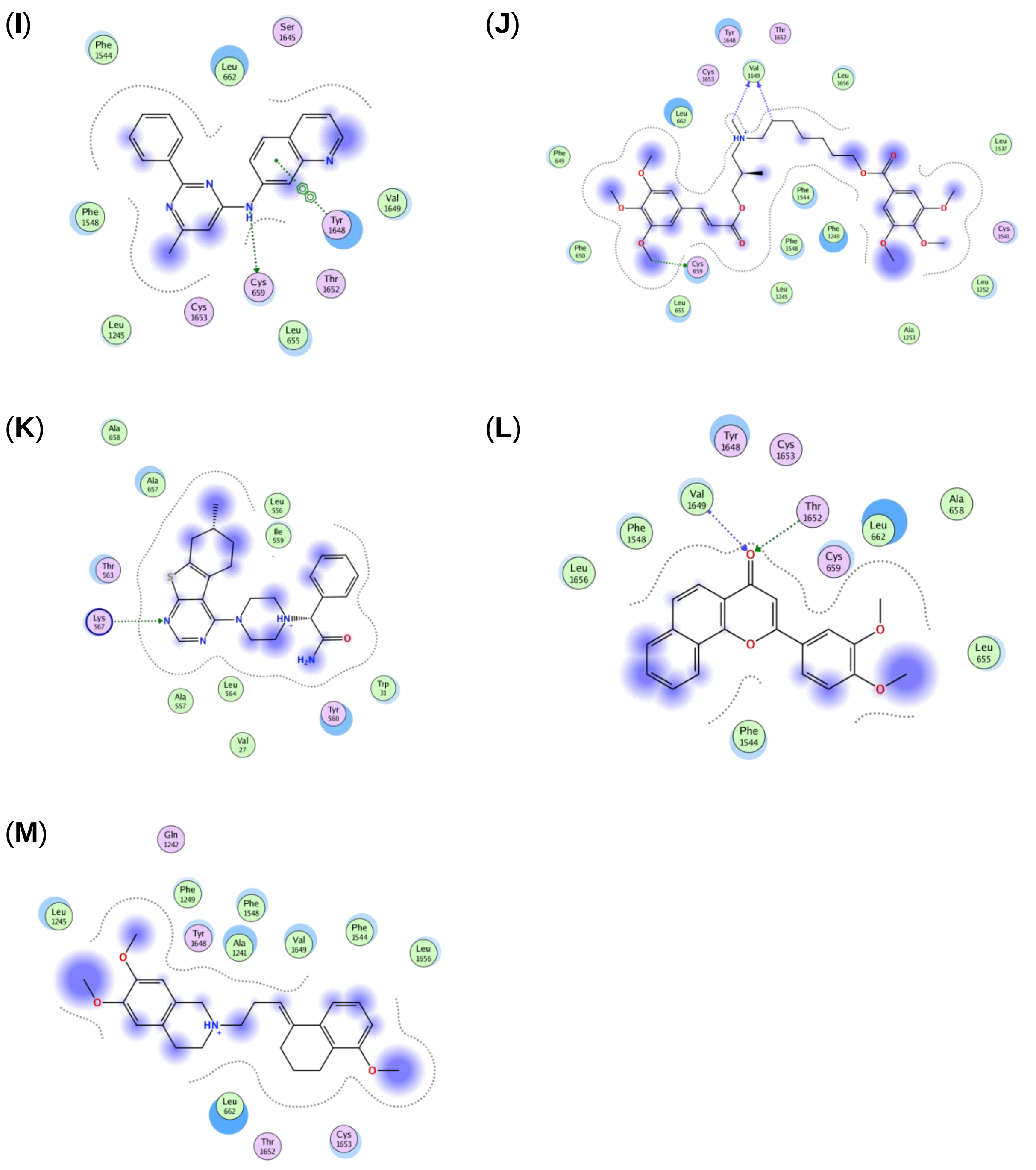

Supplementary Figure S17. 2D interaction diagram of the top ranking poses of the 13 docked pan-ABC transporter inhibitors **6** (**A**), **7** (**B**), **8** (**C**), **12** (**D**), **13** (**E**), **15** (**F**), **16** (**G**), **18** (**H**), **19** (**I**), **20** (**J**), **21** (**K**), **27** (**L**), and **28** (**M**) [45, 130-133, 138-140] in the homology model of ABCA7 using AutoDock [129]. Nitrogen, oxygen, sulfur, fluorine, chlorine, and bromine atoms were colored in blue, red, dark yellow, light green, green, and dark red, respectively.

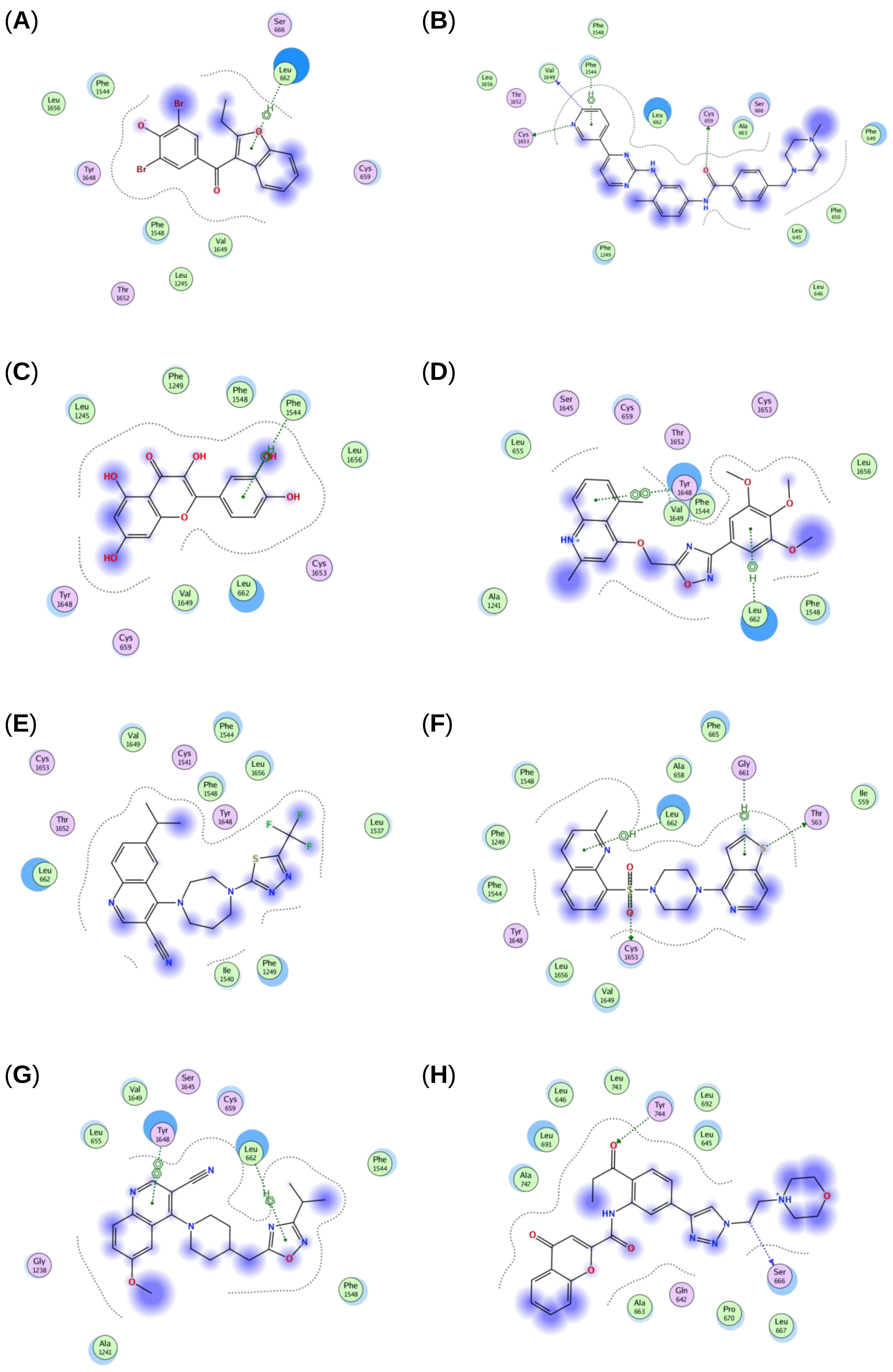
(A)
Ser 666
Leu 662
Phe 1544
Leu 1656
Br
O
Br
O
Tyr 1648
Cys 659
Phe 1548
Val 1649
Leu 1245
Thr 1652
(B)
Phe 1548
Val 1649
Phe 1544
Leu 1656
Thr 1652
Leu 662
Cys 659
Ser 666
Cys 1653
Ala 663
Phe 649
Phe 650
Leu 645
Phe 1249
Leu 646
(C)
Phe 1249
Phe 1548
Phe 1544
Leu 1245
Leu 1656
HO
O
OH
OH
OH
HO
Cys 1653
Tyr 1648
Val 1649
Leu 662
Cys 659
(D)
Ser 1645
Cys 659
Cys 1653
Leu 655
Thr 1652
Leu 1656
Tyr 1648
Val 1649
Phe 1544
HN+
Ala 1241
Leu 662
Phe 1548
(E)
Cys 1653
Val 1649
Phe 1544
Cys 1541
Leu 1656
Phe 1548
Thr 1652
Tyr 1648
Leu 1537
Leu 662
F
F
F
S
N
N
Ile 1540
Phe 1249
(F)
Phe 665
Phe 1548
Ala 658
Gly 661
Ile 559
Leu 662
Thr 563
Phe 1249
Phe 1544
Tyr 1648
Cys 1653
Leu 1656
Val 1649
(G)
Val 1649
Ser 1645
Cys 659
Leu 655
Tyr 1648
Leu 662
Phe 1544
Gly 1238
Phe 1548
Ala 1241
(H)
Leu 646
Leu 743
Tyr 744
Leu 692
Leu 691
Leu 645
Ala 747
HN
NH
Ser 666
Ala 663
Gln 642
Pro 670
Leu 667

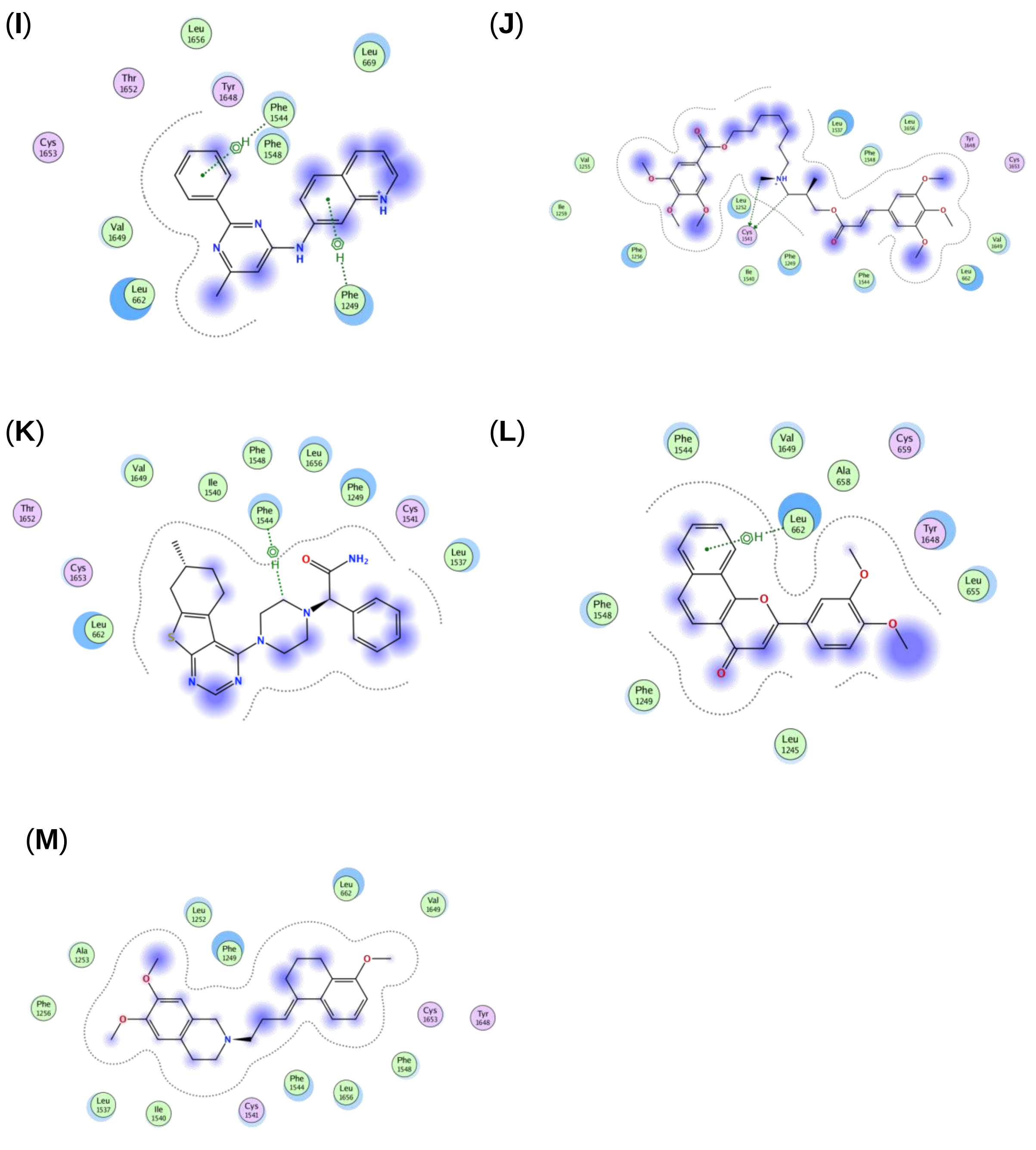

Supplementary Figure S18. 2D interaction diagram of the top ranking poses of the 13 docked pan-ABC transporter inhibitors **6** (**A**), **7** (**B**), **8** (**C**), **12** (**D**), **13** (**E**), **15** (**F**), **16** (**G**), **18** (**H**), **19** (**I**), **20** (**J**), **21** (**K**), **27** (**L**), and **28** (**M**) [45, 130-133, 138-140] in the homology model of ABCA7 using Glide [141, 142]. Nitrogen, oxygen, sulfur, fluorine, chlorine, and bromine atoms were colored in blue, red, dark yellow, light green, green, and dark red, respectively.

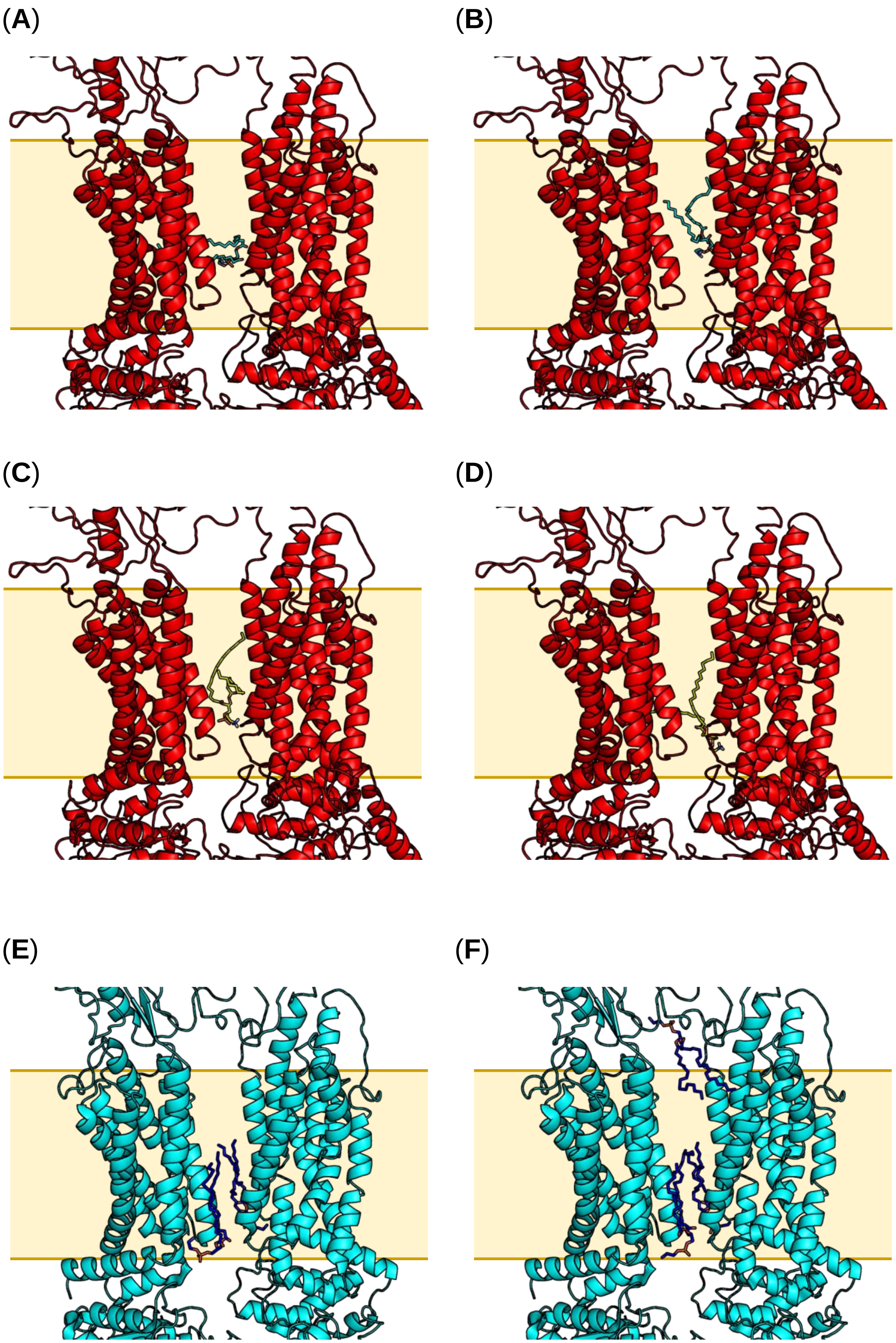
(A)
(B)
(C)
(D)
(E)
(F)

Supplementary Figure S19. The top ranking poses of the phospholipids PL1 (**A**, **C**) and PL2 (**B**, **D**) using AutoDock [129] (colored cyan, stick representation; **A**, **B**), and Glide [141, 142] (colored yellow, stick representation; **C**, **D**), in the homology model of ABCA7 (colored red, cartoon representation; the inter-membrane space is indicated as light brown area, and the border to the cytosol and lumen is indicated by brown lines). Discovered phospholipids (colored dark blue, stick representation; **E**, **F**) obtained from the cryo-EM structures of ABCA4 (colored cyan, cartoon representation, PDB IDs 7E7I [98] and 7E7O) are shown for comparison reasons. Nonpolar hydrogen atoms were omitted, and polar hydrogen, carbon, nitrogen, oxygen as well as phosphorus atoms were colored in silver, cyan, blue, red, and orange, respectively.
